# Harnessing MDM2‐Mediated Targeted Degradation of Transcriptional and Epigenetic Machinery to Disrupt Oncogenic Addictions in Pediatric Sarcoma

**DOI:** 10.1002/advs.202523088

**Published:** 2026-06-19

**Authors:** Jiawei Zhou, Xian Guan, Nan Li, Ying Zhang, Long Xie, Xingze Huang, Zhipeng Zhu, Zhuolin Ren, Xiaoyan Yu, Hanjun Guo, Yuanfang Wu, Lin Ma, Suya Zheng, Jingyao Zhang, Jiyang Liu, Victor Kuanmin Lee, Wenhao Chen, H. Phillip Koeffler, Jinhu Wang, Xin Han, Ye Chen, Liang Xu

**Affiliations:** ^1^ Department of Surgical Oncology Children's Hospital Zhejiang University School of Medicine Hangzhou China; ^2^ Institute of Biochemistry College of Life Sciences Zhejiang University Hangzhou China; ^3^ Pediatric Cancer Research Center National Clinical Research Center for Children and Adolescents’ Health and Diseases Hangzhou China; ^4^ Cancer Institute (Key Laboratory of Cancer Prevention and Intervention China National Ministry of Education) of the Second Affiliated Hospital and Institute of Translational Medicine Zhejiang University School of Medicine Hangzhou China; ^5^ Department of Orthopedic Surgery Children's Hospital Zhejiang University School of Medicine Hangzhou China; ^6^ Department of Pathology Yong Loo Lin School of Medicine National University of Singapore Singapore Singapore; ^7^ Department of Pathology National University Hospital Singapore Singapore; ^8^ Division of Hematology/Oncology Cedars‐Sinai Medical Center Los Angeles California USA; ^9^ Cancer Center of Zhejiang University Hangzhou China; ^10^ Department of Radiation Oncology (Key Laboratory of Cancer Prevention and Intervention China National Ministry of Education), The Second Affiliated Hospital Zhejiang University School of Medicine Hangzhou China

**Keywords:** CDK9, MDM2, PROTAC, P‐TEFb, targeted protein degradation, transcriptional addiction

## Abstract

Despite considerable pathological diversity, pediatric sarcomas lack molecularly targeted treatments, demanding deeper pathobiological insights and innovative therapeutic strategies. Here, we demonstrate that overexpressed MDM2 functions as an important pathogenic driver in these malignancies, rewiring oncogenic programs through both p53‐independent chromatin occupancy to regulate active transcription and conventional proteasome‐mediated p53 degradation leading to pathway suppression. To leverage this dependency for targeted eradication of pediatric sarcomas with MDM2 overexpression, we develop MDM2‐recruiting proteolysis‐targeting chimeras that selectively degrade the CDK9/Cyclin T complex (P‐TEFb). Among the lead compounds, dCDK9‐010 demonstrates superior activity compared to its parental CDK9 inhibitor or MDM2 antagonist either alone or in combination, by coordinatedly disrupting the MDM2‐p53 axis and super‐enhancer‐driven transcription. Remarkably, the transcriptional effects of P‐TEFb degradation by dCDK9‐010 are phenocopied by MDM2‐mediated BET degradation, resulting in potent anti‐sarcoma efficacy alongside a favorable therapeutic index and minimal toxicity in nonmalignant cells. Moreover, these MDM2‐recruiting transcriptional/epigenetic machinery degraders (termed MDM2‐TEMADs) consistently impair the homologous recombination repair pathway and confer synthetic lethality with PARP inhibitors. Together, this work elucidates MDM2's central role in pediatric sarcoma pathogenesis and presents dCDK9‐010 as a first‐in‐class, MDM2‐recruiting P‐TEFb degrader and an exemplary MDM2‐TEMAD that enables precise targeting of MDM2‐dependent oncogenic transcriptional addiction.

## Graphical abstract

MDM2 dependency in pediatric sarcomas is driven by a novel p53‐independent oncogenic cistrome alongside canonical p53 pathway suppression. This study introduces MDM2‐recruiting transcriptional and epigenetic machinery degraders (MDM2‐TEMADs) as a novel precision oncology modality. The newly developed MDM2‐recruiting P‐TEFb degrader, dCDK9‐010, coordinately disrupts oncogenic transcription and induces homologous recombination deficiency, achieving potent, tumor‐selective cytotoxicity in MDM2‐driven pediatric sarcomas.

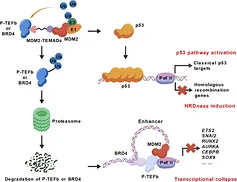

## 1 Introduction

Pediatric sarcomas comprise 10%–15% of malignant childhood cancers [1]. Major subtypes include osteosarcoma (OS), Ewing sarcoma (EWS), and rhabdomyosarcoma, which are often driven by genetic alterations involving transcriptional dysregulation, such as *TP53* inactivation, *MYC*/*MYCN* amplification, and characteristic oncofusions like *EWSR*::*FLI1* [2, 3, 4, 5]. Current treatment relies on surgery, chemotherapy, and radiotherapy, but is often limited by tumor location, toxicity, and resistance. Targeted therapies remain scarce, with most still under clinical investigation [6], highlighting an urgent need for more effective and selective therapeutics.

A hallmark of pediatric sarcomas is transcriptional addiction, a dependency on dysregulated transcription for maintaining malignant identity and phenotypes. This addiction is mediated through hyperactivated transcription factors (TFs), super‐enhancer (SE) engagement, and aberrant chromatin remodeling [7, 8]. Key mediators of this process include transcription‐associated cyclin‐dependent protein kinases (CDKs, e.g., CDK7, CDK9) and transcriptional co‐activators such as BET (bromodomain and extraterminal domain) family proteins. Both transcription‐associated CDKs and BET proteins are frequently co‐opted by cancer cells to facilitate proficient transcription [9]. CDK9 and Cyclin T form the positive transcription elongation factor b (P‐TEFb) complex, which interacts with various chromatin regulators (e.g., BRD4, AFF1/4) and TFs (e.g., MYC) to enforce enhancer activity and promote productive transcription elongation [10]. While P‐TEFb is recruited to promoters by BET proteins and phosphorylates the C‐terminal domain (CTD) of RNA polymerase II (Pol II) Rpb1 subunit at Ser2, BRD4 can also directly phosphorylate Rpb1 through its atypical kinase activity, indicating a P‐TEFb‐independent role in transcription elongation [11]. Although the pathobiological roles of P‐TEFb and BRD4 dependency in pediatric sarcomas remain incompletely characterized, inhibitors targeting CDK9 or BRD4 have shown antitumor potential by disrupting transcription and the basal transcriptional machinery [12, 13]. Nevertheless, their clinical advancement has been hampered by narrow therapeutic windows and dose‐limiting toxicities, emphasizing the need for more precise targeting strategies.

Proteolysis‐targeting chimeras (PROTACs) represent an innovative therapeutic approach. These bifunctional molecules recruit intracellular E3 ubiquitin ligases to induce targeted protein degradation via the ubiquitin‐proteasome system [14]. By catalytically eliminating both enzymatic and scaffolding functions of proteins, PROTACs offer a mechanistic advantage over conventional inhibition [15]. We and others have reported several CRBN‐ or VHL‐dependent PROTACs targeting CDK9 or BET proteins, demonstrating strong antitumor efficacy [16, 17, 18, 19]. However, since CRBN and VHL are broadly expressed, these PROTACs often lack tumor selectivity. Additionally, CRBN‐recruiting ligands often retain undesirable molecular glue activity, leading to unintended degradation of IKZF1/3 and/or GSPT1 [14]. The development of tumor‐selective PROTACs with enhanced specificity thus remains a continuing challenge and a driving interest in the fields of pharmacology and targeted cancer therapy.

MDM2 is an oncogenic E3 ubiquitin ligase best known for targeting the tumor suppressor p53 for degradation. The MDM2‐p53 pathway is frequently dysregulated in pediatric sarcomas, often through *TP53* inactivation or *MDM2* copy number gain/amplification [20]. MDM2 is an attractive therapeutic target in *TP53*‐wildtype sarcomas [21]. Small‐molecule inhibitors (e.g., Idasanutlin, Siremadlin, and DS‐3032) disrupt the MDM2‐p53 interaction, leading to p53 stabilization and subsequent activation of p53‐mediated transcriptional programs, and induction of cell cycle arrest and apoptosis [20]. Beyond inhibition, innovative strategies have been developed to redirect MDM2's E3 ligase activity toward neo‐substrates. For example, hybrid peptides and small molecule conjugates have been designed to recruit MDM2 to degrade proteins such as BRD4 [22, 23]. However, early attempts to repurpose MDM2 for degrading CDK proteins (e.g., CDK6) have proven unsuccessful [24]. Hence, while MDM2's E3 ligase activity can be reprogrammed, its substrate permissiveness remains restricted and requires further expansion.

In this study, we establish that MDM2 drives malignant transcriptional programs in pediatric sarcomas through both a p53‐independent cistrome and canonical p53 suppression. To therapeutically exploit this MDM2 dependency and its sarcoma‐specific overexpression, we developed first‐in‐class MDM2‐recruiting P‐TEFb PROTACs that deplete all CDK9 and Cyclin T isoforms while stabilizing p53. Our lead compound, dCDK9‐010, exhibits superior anti‐sarcoma efficacy by coordinately disrupting enhancer‐driven transcription, p53‐independent MDM2 cistrome, the MDM2‐p53 axis, and the homologous recombination (HR) repair pathway. Remarkably, the transcriptional and cellular effects induced by dCDK9‐010 are closely recapitulated by an MDM2‐recruiting BET PROTAC (A1874), revealing a convergent mechanism among the MDM2‐recruiting transcriptional/epigenetic machinery degraders (MDM2‐TEMADs) in targeting oncogenic transcriptional addiction.

## 2 Results

### 2.1 MDM2 Promotes Sarcomagenesis Through Both p53‐Dependent and p53‐Independent Transcriptional Regulatory Mechanisms

**FIGURE 1:**
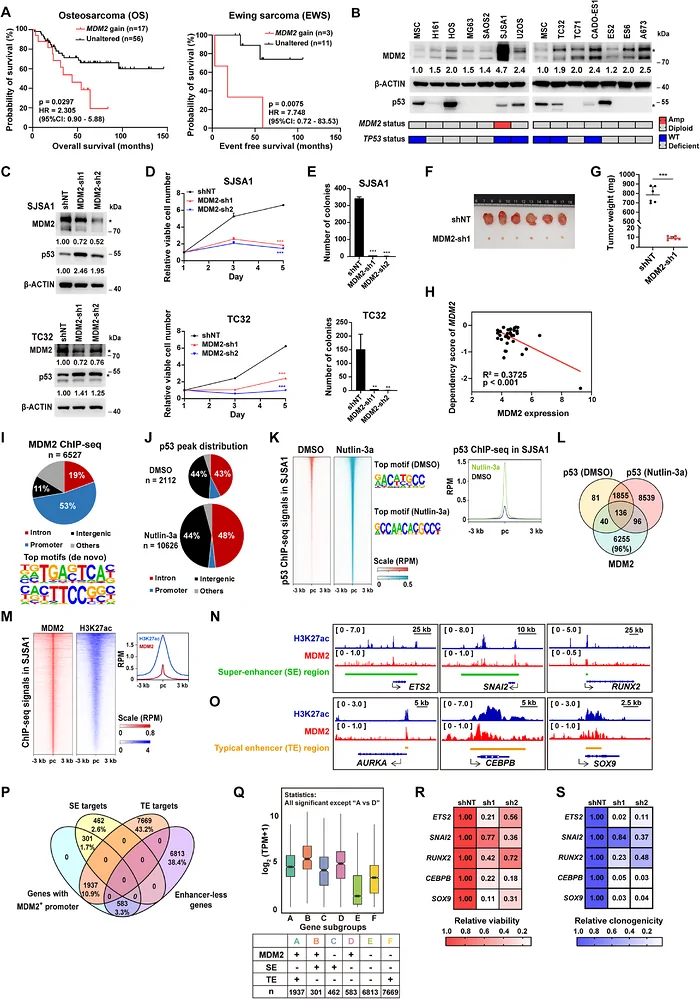
MDM2 promotes pediatric sarcoma malignancy through both p53‐dependent and p53‐independent mechanisms. (A) Survival stratification of OS (TARGET cohort, n = 73) and EWS (OpenPedCan Project v15 cohort, n = 14) patients based on *MDM2* copy numbers in sarcoma genomes. B) MDM2 protein levels across mesenchymal stem cell (MSC), OS, and EWS cell lines. The relative expression of MDM2 in each sarcoma cell line was normalized to β‐actin and subsequently compared to its expression level in MSC. * indicates the primary band at the expected molecular weight. (C–E) Effects of shRNA‐mediated MDM2 knockdown in sarcoma cells on (C) p53 expression, (D) cell viability, and (E) soft‐agar colony‐formation capability. (F, G) Effect of MDM2 knockdown on the subcutaneous tumorigenicity of SJSA1 cells in immunocompromised mice. (H) Correlation analysis of *MDM2* mRNA level and functional dependency (DepMap score derived from CRISPR perturbation effects) across a panel of OS (n = 17) and EWS (n = 17) cell lines. (I) Genomic occupancy of MDM2 in SJSA1 cells. (J, K) Genomic occupancy of p53 in SJSA1 cells treated with either DMSO or Nutlin‐3a (10 µm, 12 h). RPM, reads per million mapped reads; pc, peak center. (L) Venn diagram comparing genomic binding sites of p53 and MDM2. (M) Co‐occupancy of MDM2 with H3K27ac across the SJSA1 genome. (N, O) Co‐occupancy of H3K27ac and MDM2 across the enhancers and promoters of representative sarcoma‐promoting genes. (P, Q) Classification and expression analysis of gene clusters based on enhancer status and MDM2 occupancy at promoters in SJSA1 cells. TPM, transcripts per kilobase of exon model per million mapped reads. (R, S) Effects of shRNA‐mediated gene knockdown on (R) viability and (S) clonogenicity of SJSA1 cells. See related data in Figure S1M,N. Data of (D, E, G) are presented as mean±SEM; (D, E), n = 3; (G), n = 6. Statistics: (A) Log‐rank, (D, E) one‐way ANOVA, (G) Student's t‐test (two tailed), (H) Spearman test, (Q) Kruskal–Wallis test with post‐hoc Dunn's test. **p < 0.01; ***p < 0.001.

Based on genomic analysis of pediatric sarcomas [25, 26, 27], we revealed significant gains or amplifications in *MDM2* copy numbers, occurring in approximately 8%–20% of OS and 20% of EWS specimens. Remarkably, OS and EWS patients with *MDM2* copy number gains (including amplifications) had a worse prognosis (Figure 1A). Consistent with these frequent genomic alterations in clinical samples, higher *MDM2* mRNA expression also predicts shorter overall survival in EWS patients (Figure S1A). Importantly, MDM2 protein was abundantly expressed in OS and EWS cell lines relative to mesenchymal stem cells (MSCs) (Figure 1B). Based on *MDM2* status and steady‑state protein abundance, we selected SJSA1 (*MDM2*‐amplified, *TP53*‐wildtype OS cell line) as a genome‐driven MDM2‑overexpressing model, and TC32 (*MDM2*‐diploid, *TP53*‐wildtype EWS cell line with intermediate MDM2 overexpression relative to MSCs) as an *MDM2*‐proficient model for investigating MDM2 function in pediatric sarcoma.

Next, we performed loss‐of‐function studies using shRNA‐mediated knockdown of MDM2 in sarcoma cell lines. Silencing MDM2 resulted in elevated p53 level in both SJSA1 and TC32 cells, and abrogated their viability and soft agar colony‐formation capability (Figure 1C–E, Figure S1B). Importantly, *MDM2*‐deficient SJSA1 cells lost the capacity for xenograft tumor formation in vivo (Figure 1F,G), supporting the critical role of MDM2 in sarcomagenesis. Further analysis of a functional screening dataset revealed a strong anti‐correlation between CRISPR‐based perturbation effects and *MDM2* mRNA levels across the OS and EWS cell line panel (Figure 1H), suggesting MDM2 as a broad functional dependency in these malignancies.

Analysis of MDM2 subcellular distribution in SJSA1 cells revealed that a proportion of MDM2 co‐fractionated with JUN and Histone H3 (Figure S1C), indicating its recruitment to chromatin. To gain molecular insights into the chromatin‐bound MDM2 in pediatric sarcomas, we performed chromatin immunoprecipitation sequencing (ChIP‐seq) of MDM2 in SJSA1 cells. We identified 6527 high‐confidence MDM2 peaks, of which 53%, 19%, and 11% were located in promoter, intronic, and intergenic regions, respectively (Figure 1I and Table S1). Given the well‐established interaction between MDM2 and p53 [28, 29], we next investigated whether these proteins co‐occupy across chromatin. Under basal conditions, p53 exhibited relatively weak chromatin binding (Figure 1J,K, Figure S1D and Table S2). MDM2 inhibitor treatment robustly stabilized p53 and enhanced its chromatin occupancy, leading to activation of the canonical p53 cistrome and anti‐viability responses (Figure 1J,K, Figure S1D,E and Table S3). However, 96% of MDM2 binding sites did not overlap with either steady‐state p53 sites or those acquired upon p53 stabilization (Figure 1L). Furthermore, the genomic distribution and motif enrichment profiles of p53 were clearly distinct from those of MDM2 (Figure 1I–K, Figure S1F). These findings strongly support a model in which MDM2 occupies sarcoma chromatin through a p53‐independent mechanism.

Instead, MDM2 peaks showed extensive overlap with H3K27ac signals and were strongly enriched for known motifs of Activator protein‐1 (AP‐1), E26 transformation specific (ETS), and Yin Yang 1 (YY1) TFs (Figure 1M, Figure S1F), suggesting cooperative roles in active transcription. Of note, YY1 and the AP‐1 transcription factor JUN have been reported as putative co‐factors for chromatin‐associated MDM2 in adult liposarcoma [28]. In the present study, we identified ETS2 as a binding partner of MDM2 (Figure S1G). Knockdown of ETS2 diminished both total level and chromatin‐bound MDM2 levels in SJSA1 cells (Figure S1H). Genome‐wide occupancy analysis revealed that 20% of MDM2‐binding sites are co‐occupied by ETS2 (Figure S1I,J), suggesting that ETS2 constitutes an additional putative cooperative factor of MDM2. Given the promoter‐centric distribution of MDM2 binding, we annotated genes with promoter‐proximal MDM2 peaks as “MDM2^+^ promoter targets”. Using H3K27ac ChIP‐seq data, we inferred SEs and typical enhancers (TEs), and classified *MDM2* along with several TFs (e.g., *RUNX2*, *ETS2*, and *SNAI2)* as SE‐driven genes in SJSA1 cells (Figure 1N, Figure S1K, and Table S4). *RUNX2*, *ETS2*, and *SNAI2* also showed MDM2 binding at their promoters. Of note, MDM2 binding was also evident at the loci of TE‐associated prominent mitotic genes (e.g., *AURKA*) and lineage‐specific genes (e.g., *SOX9* and *CEBPB*) (Figure 1O, and Tables S1 and S5), suggesting the active transcription of MDM2 cistrome. To examine the functional impact of MDM2 occupancy on transcription, we categorized genes by MDM2 promoter binding and enhancer status (Figure 1P). MDM2^+^ promoter targets were among the most highly expressed genes, with the subset of SE‐driven, MDM2^+^ promoter targets showing the highest expression levels (Figure 1Q). Functional assays confirmed strong growth dependency of SJSA1 cells on these MDM2‐bound TF genes (*ETS2*, *RUNX2*, *SNAI2*, *SOX9*, and *CEBPB*) (Figure 1R,S, Figure S1L–N), and *ETS2* was further validated as essential in the EWS cell line TC32 (Figure S1O–Q). Together, these results establish that MDM2 promotes sarcomagenesis through both p53‐dependent and p53‐independent transcriptional regulatory mechanisms.

### 2.2 Essential Roles of P‐TEFb and BRD4 in Maintaining p53‐Independent MDM2 Cistrome

Consistent with the established reliance of oncogenic transcription on P‐TEFb and BET function [30, 31], pharmacological inhibition of CDK9 kinase activity or BET bromodomains (BDs) reduced expression of enhancer‐driven MDM2 targets, including SE‐associated *RUNX2*, *ETS2*, and *SNAI2*, and TE‐associated *SOX9*, *CEBPB*, and *AURKA* (Figure S2A,B). These findings indicate that the active MDM2 cistrome is transcriptionally addicted to core components of transcriptional machinery. Parallel genetic and functional studies further validated the essential roles of CDK9 and BRD4 in sustaining sarcoma cell growth both in vitro and in vivo (Figure S2C–J). Additionally, CRISPR/Cas9‐mediated silencing of *CCNT1* and *CCNT2* attenuated cellular viability and clonogenicity (Figure S2K–P), though to a lesser degree than *CDK9* knockdown. These results reveal a profound transcriptional and growth dependency on P‐TEFb and BET proteins in MDM2‐driven pediatric sarcomas, highlighting a promising therapeutic vulnerability amenable to targeted intervention.

### 2.3 Development of Novel MDM2‐Recruiting P‐TEFb Degraders With Selective and Superior Cytotoxic Activities in Pediatric Sarcomas

**FIGURE 2:**
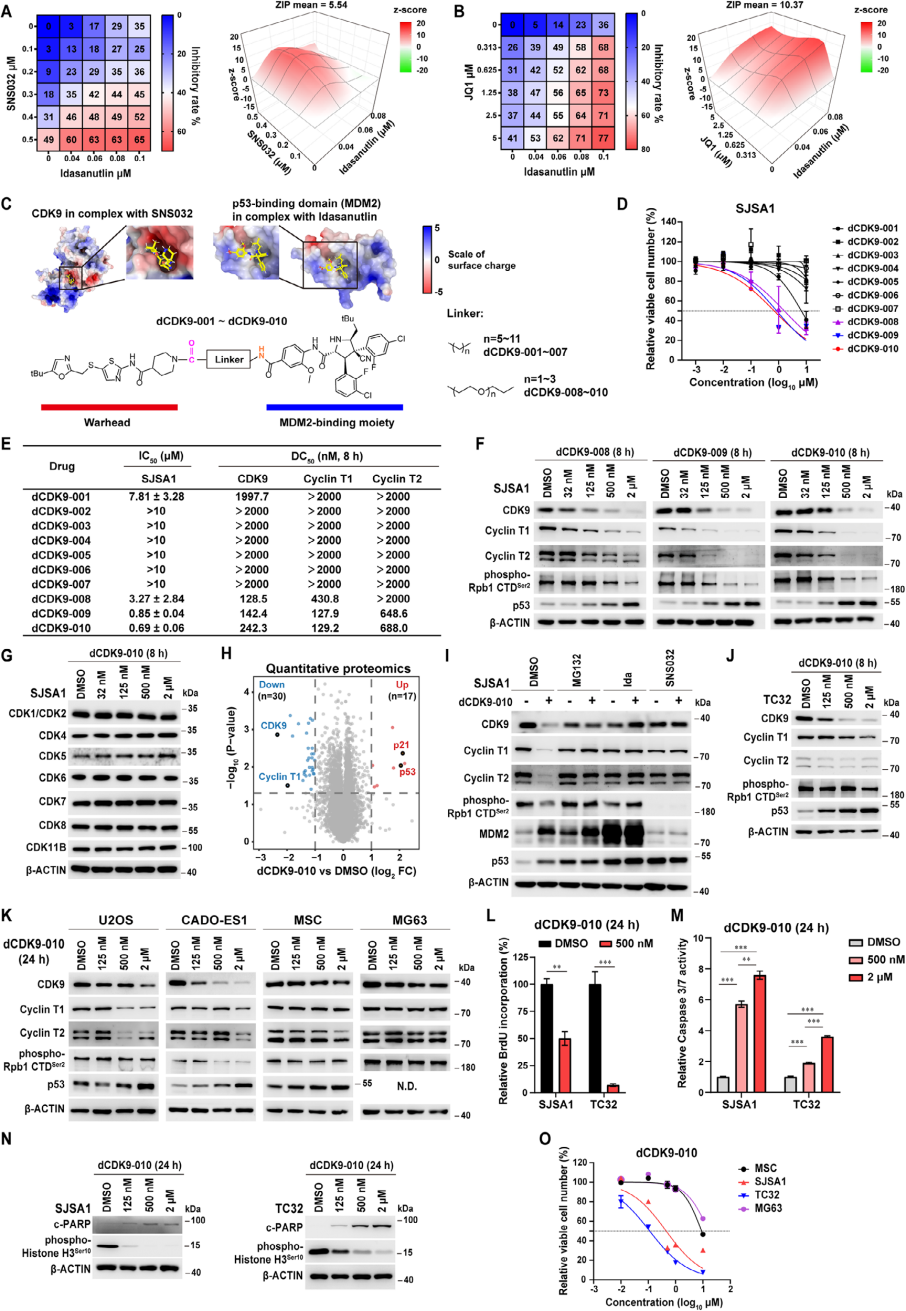
Design and evaluation of novel MDM2‐recruiting P‐TEFb degraders. (A, B) Effects of combination treatment with the MDM2 inhibitor Idasanutlin and transcriptional inhibitors (SNS032 and JQ1) on cell viability in SJSA1 cells. Drug interactions were evaluated using the Zero Interaction Potency (ZIP) synergy model. (C) Molecular docking analysis of SNS032 bound to the CDK9 kinase domain (PDB ID: 8K5R) and Idasanutlin complexed with the p53‐binding domain of MDM2 (PDB ID: 4JRG). Solvent‐exposed functional groups on both inhibitors were selected as tethering points for designing dCDK9‐001 to dCDK9‐010 compounds. See related Table S6. (D) Dose‐response curves demonstrating the anti‐proliferative effects of the heterobifunctional compounds in SJSA1 cells. (E) The half maximal inhibitory concentrations (IC_50_) and half‐maximal CDK9/Cyclin T degradation concentrations (DC_50_) of the newly developed heterobifunctional compounds in SJSA1 cells. IC_50_ values are presented as mean±SEM; n = 3. (F) Dose‐dependent effects of dCDK9‐008∼010 on target protein expression. (G) Impact of 8‐h dCDK9‐010 treatment on intracellular CDK protein levels. (H) Quantitative proteomics analysis of SJSA1 cells treated with DMSO or dCDK9‐010 (500 nm, 8 h). (I) Effects of MG132 (10 µm), SNS032 (20 µm), and Idasanutlin (Ida, 20 µm) pretreatments on dCDK9‐010's CDK9/Cyclin T degrading activity in SJSA1 cells. Cells were pretreated with indicated compounds for 2 h, followed by 4‐h treatment with dCDK9‐010 at 1 µm. (J, K) Effects of dCDK9‐010 treatment on target proteins in indicated cells. (L) Inhibition of cellular BrdU incorporation by dCDK9‐010. (M) Induction of intracellular Caspase 3/7 activity by dCDK9‐010 in sarcoma cells. (N) Effects of dCDK9‐010 treatment (24 h) on cleaved‐PARP1 and phospho‐Histone H3 levels in sarcoma cells. (O) Dose‐response curves showing the effects of dCDK9‐010 on the viability of MSC and indicated sarcoma cells. Data in (D, L, M, O) are presented as mean±SEM; n = 3. Statistics: (L) Student's t‐test (two‐tailed), (M) one‐way ANOVA. **p < 0.01; ***p < 0.001.

Given MDM2's potent sarcoma‐promoting functions through both p53‐dependent and p53‐independent mechanisms, we hypothesized that simultaneously targeting both pathways would yield optimal therapeutic efficacy. Initial support arose from combining the MDM2 inhibitor Idasanutlin with transcriptional inhibitors (SNS032 or JQ1), which produced either additive or synergistic anti‐sarcoma activity in vitro (Figure 2A,B).

To more strategically target MDM2's multifaceted oncogenic functions, we leveraged its intrinsic E3 ubiquitin ligase activity for targeted protein degradation. We designed and synthesized a series of heterobifunctional molecules (dCDK9‐001∼dCDK9‐010) by conjugating the MDM2 ligand Idasanutlin to the CDK9 inhibitor SNS032 through diverse linker architectures (Figure 2C). Structure‐activity relationship analysis in SJSA1 cells, based on anti‐proliferative activity (IC_50_) and P‐TEFb (CDK9, Cyclin T1, Cyclin T2) degradation efficiency (DC_50_), revealed that polyethylene glycol (PEG) linkers generally conferred better efficacy than alkyl chains (Figure 2D,E, Figure S3A,B, and Table S6). Remarkably, PEG‐linked conjugates (dCDK9‐008∼dCDK9‐010) induced dose‐dependent depletion of the P‐TEFb complex and its substrate phospho‐Rpb1 CTD^Ser2^, while concurrently stabilizing p53 (Figure 2F). Among the series, dCDK9‐010 emerged as the lead compound, demonstrating optimal anti‐proliferative activity and submicromolar DC_50_ values against P‐TEFb, with high selectivity over other CDKs including known SNS032 targets (CDK2 and CDK7) (Figure 2D–H). To systematically profile dCDK9‐010 targets, we performed a quantitative data‐independent acquisition mass spectrometry analysis of SJSA1 cells. Among the 47 differentially expressed proteins, CDK9 and Cyclin T1 were identified as the top down‑regulated targets following 8 h of dCDK9‑010 treatment, whereas p53 and p21 were induced (Figure 2H, and Table S7). Notably, dCDK9‑010 exhibited minimal off‑target effects on other detected CDK family members or cyclins, highlighting its superior selectivity. Although dCDK9‐010 and dCDK9‐009 share similar structures but differ in PEG linker length, this structural variation influenced their target degradation kinetics and cell‐inhibitory efficacy. At early time points (8 h), dCDK9‐009 exhibited relatively stronger target degradation efficacy than dCDK9‐010 (Figure 2F). However, starting from 16 h of treatment, dCDK9‐010 exhibited moderately higher efficacy than dCDK9‐009 in degrading CDK9 and Cyclin T1, as well as inducing p53 and p21 (Figure S3C). Consistently, dCDK9‐010 also displayed slightly better anti‑proliferative activity than dCDK9‐009. Its degradation mechanism was confirmed as proteasome‐ and MDM2‐dependent, as activity was abolished by proteasome inhibition, or competition with excess SNS032 or Idasanutlin (Figure 2I).

Remarkably, dCDK9‐010 consistently induced P‐TEFb degradation and p53 stabilization across multiple pediatric sarcoma lines (TC32, U2OS, and CADO‐ES1), while showing minimal activity in human mesenchymal stem cells (MSCs) and the *TP53*‐null OS line MG63 (Figure 2J,K). In sensitive SJSA1 and TC32 cells, this on‐target activity translated into potent cytotoxicity, characterized by compromised BrdU incorporation and phospho‐Histone H3 levels, activation of apoptotic Caspase 3/7, and PARP1 cleavage (Figure 2L–N). This compound's cell selectivity was further reflected in IC_50_ values that were at least 10‐fold higher in resistant MSCs and MG63 cells compared to sensitive sarcoma cells (Figure 2O), establishing dCDK9‐010 as a potent and selective MDM2‐recruiting P‐TEFb degrader with a favorable therapeutic index.

### 2.4 MDM2‐Recruiting P‐TEFb Degrader Preferentially Disrupts Enhancer‐Driven MDM2 Cistrome

**FIGURE 3:**
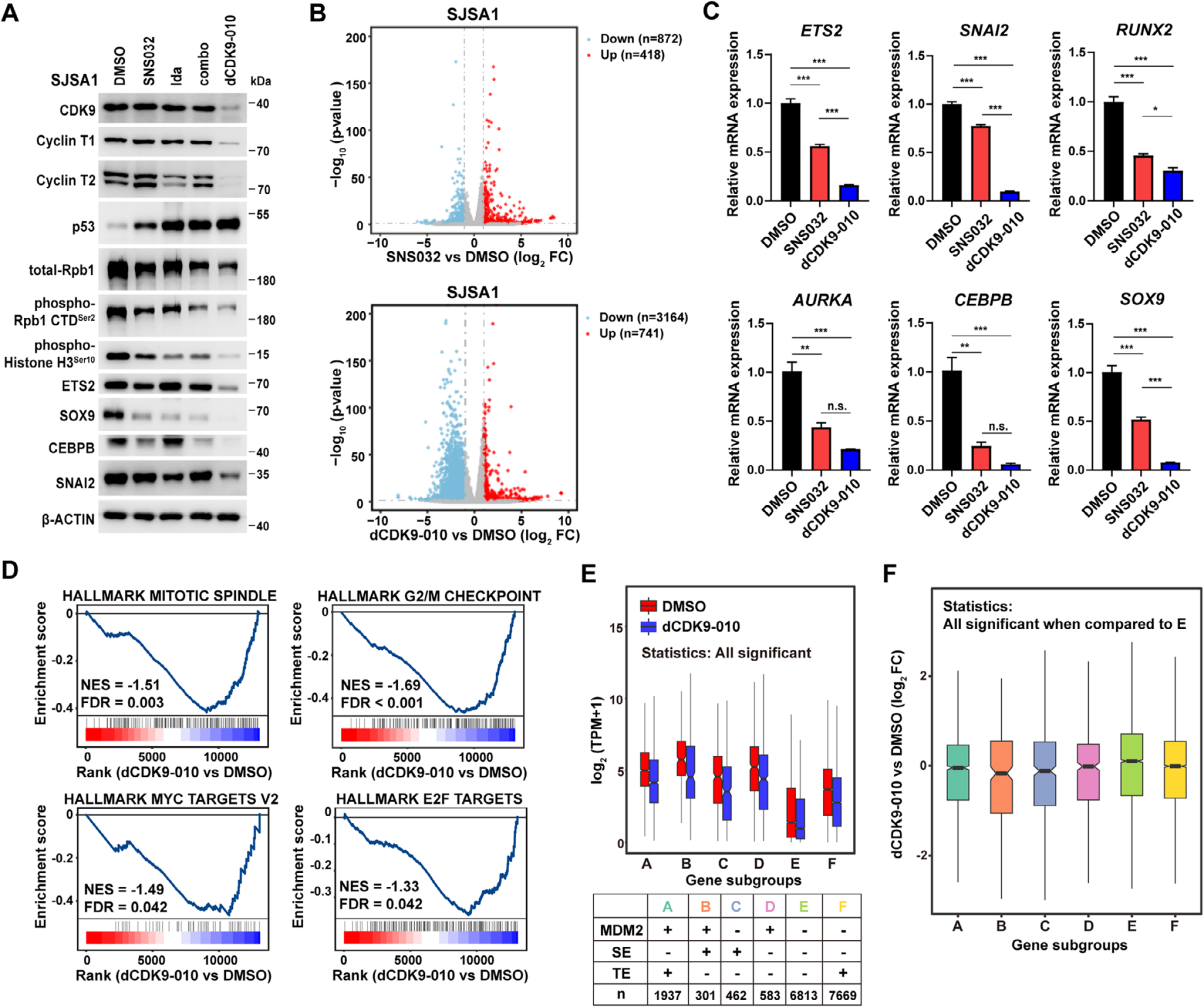
dCDK9‐010 simultaneously disrupts the enhancer‐driven transcriptome and the MDM2‐associated cistrome in pediatric sarcoma cells. (A) Impacts of SNS032, Idasanutlin (Ida), SNS032/Idasanutlin combo (SNS032+Ida), and dCDK9‐010 treatments (500 nm, 24 h) on indicated proteins in SJSA1 cells. (B) Differentially expressed genes in SJSA1 cells receiving SNS032 and dCDK9‐010 treatments (500 nm, 8 h). (C) qPCR analysis of MDM2 targets in response to either SNS032 or dCDK9‐010 treatment (500 nm, 8 h) in SJSA1 cells. Data are presented as mean±SEM; n = 3. One‐way ANOVA was applied. *p < 0.05; **p < 0.01; ***p < 0.001. (D) Negative enrichment of the mitotic spindle genes, G2/M checkpoint genes, and MYC/E2F targets in dCDK9‐010‐treated SJSA1 cells. NES, normalized enrichment score. (E, F) Robust suppression of gene clusters driven by active enhancers or marked by MDM2 occupancy at promoter regions following dCDK9‐010 treatment (500 nm, 8 h) in SJSA1 cells. Statistics: (E) Wilcoxon signed‐rank test, (F) Kruskal–Wallis test with post‐hoc Dunn's test. FC, fold change.

Beyond its dual capacity for P‐TEFb degradation and p53 stabilization, dCDK9‐010 more potently suppressed phospho‐Rpb1 CTD^Ser2^, total‐Rpb1, phospho‐Histone H3, and core disease‐promoting TFs (e.g., ETS2, SOX9, CEBPB, and SNAI2), when compared to SNS032, Idasanutlin, or their combination (Figure 3A). These results demonstrate the superior efficacy of dCDK9‐010‐mediated P‐TEFb degradation over conventional catalytic inhibition.

Comparative transcriptomic analyses revealed that dCDK9‐010 induced more potent perturbation of global gene expression than SNS032 (Figure 3B). Specifically, qPCR demonstrated a greater impairment of key MDM2 targets, including mitotic regulators (e.g., *AURKA*) and pro‐tumorigenic TFs (e.g., *ETS2*, *RUNX2*, *SNAI2*, *SOX9*, and *CEBPB*) (Figure 3C). Corroborating these observations, gene set enrichment analysis (GSEA) further indicated significant negative enrichment of gene signatures related to the mitotic spindle, G2/M checkpoint, and MYC/E2F targets in dCDK9‐010‐treated sarcoma cells (Figure 3D). In addition to broadly suppressing active transcription, dCDK9‐010 exerted greater inhibitory impacts on both enhancer‐driven genes and MDM2^+^ promoter targets than on enhancer‐less non‐MDM2 targets (Figure 3E,F). Remarkably, SE‐associated, MDM2^+^ promoter targets exhibited the most pronounced transcriptional downregulation following dCDK9‐010 treatment. Within the MDM2 cistrome, MDM2^+^ promoter targets linked to TEs also responded more strongly to treatment than those without enhancer association (Figure 3E,F). These results demonstrate that the newly developed MDM2‐recruiting P‐TEFb degrader efficiently disrupts both enhancer‐driven oncogenic programs and the p53‐independent MDM2 cistrome.

### 2.5 MDM2‐Recruiting BET Degrader Recapitulates the Core Therapeutic Merits of P‐TEFb Degraders

**FIGURE 4:**
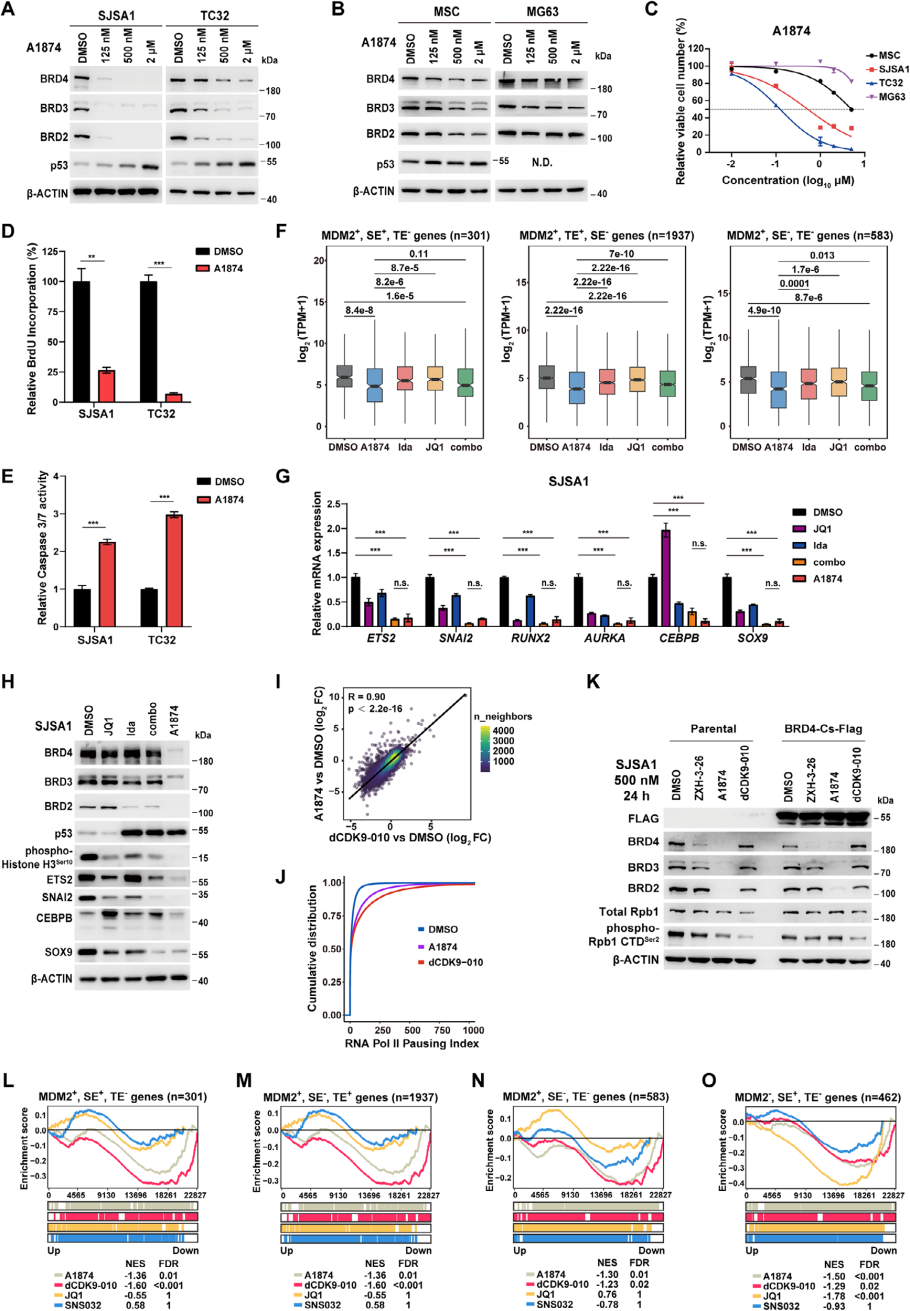
The MDM2‐recruiting BET degrader A1874 induces transcriptional changes closely mirroring those observed with dCDK9‐010 treatment. (A, B) Dose‐dependent effects of A1874 (8 h) on target protein expression in indicated cells. (C) Dose‐response curves showing the effects of A1874 on viability of MSC and indicated sarcoma cells. (D) Inhibition of cellular BrdU incorporation by A1874 (500 nm, 24 h). (E) Induction of intracellular Caspase 3/7 activity by A1874 (500 nm, 24 h) in sarcoma cells. (F) Expression of gene clusters driven by active enhancers or marked by MDM2 occupancy at promoter regions in response to JQ1, Idasanutlin (Ida), JQ1/Idasanutlin combo (JQ1+Ida), and A1874 treatments (500 nm, 8 h) in SJSA1 cells. (G) qPCR analysis of MDM2 targets in response to JQ1, Idasanutlin, JQ1/Idasanutlin combo, and A1874 treatments (500 nm, 8 h) in SJSA1 cells. (H) Impacts of JQ1, Idasanutlin, JQ1/Idasanutlin combo, and A1874 treatments (500 nm, 24 h) on BET proteins, p53, phospho‐Histone H3, and sarcoma‐promoting TFs in SJSA1 cells. (I) Transcriptomic correlation analysis between SJSA1 cells treated with dCDK9‐010 versus A1874. (J) Effect of dCDK9‐010 and A1874 treatment (500 nm, 8 h) on Pol II pausing indexes in SJSA1 cells. (K) Effects of ectopic BRD4‐Cs on the molecular response to ZXH‐3‐26, A1874, or dCDK9‐010 in SJSA1 cells. (L–O) Enrichment analyses of gene sets including (L) SE^+^, MDM2^+^ promoter targets, (M) TE^+^, MDM2^+^ promoter targets, (N) SE^−^, TE^−^, MDM2^+^ promoter targets, and (O) SE^+^, MDM2^−^ promoter targets in SJSA1 cells following dCDK9‐010, A1874, SNS032, or JQ1 treatment. Data in (C–E, G) are presented as mean±SEM; n = 3. Statistics: (D, E) Student's t test (two tailed), (F) Kruskal–Wallis test with post‐hoc Dunn's test, (G) one‐way ANOVA, (I) Spearman test. n.s., not significant; **p < 0.01; ***p < 0.001.

Based on the aforementioned dependency of MDM2‐driven pediatric sarcomas on P‐TEFb and BET proteins, we sought to examine whether MDM2‐recruiting BET degrader would mirror the selectivity and potency of our P‐TEFb degrader. To this end, we investigated A1874, an Idasanutlin‐JQ1 conjugate, for its cellular activity and anti‐sarcoma potential. Intriguingly, in SJSA1 and TC32 cells, A1874 induced dose‐dependent BET protein (BRD2, BRD3, BRD4) degradation and p53 stabilization (Figure 4A). However, this effect was absent in either MSCs or MG63 cells (Figure 4B). Consistently, A1874 more potently suppressed viability in these sensitive lines (Figure 4C), coupled with potent suppression of proliferation and induction of apoptosis via Caspase‐3/7 activation (Figure 4D,E). RNA‐seq analysis further demonstrated that A1874 exacerbated transcriptomic perturbation over either JQ1 or Idasanutlin alone (Figure S4A–C), while maintaining a highly correlated transcriptional profile with the JQ1/Idasanutlin combo (Figure S4D). Overall, A1874 produced the most potent suppression of MDM2^+^ promoter targets, especially those associated with SEs or TEs, when compared to inhibitor monotherapy or JQ1/Idasanutlin combo (Figure 4F). Specifically, A1874 reduced mRNA expression of the key enhancer‐driven MDM2^+^ promoter targets including mitotic regulator *AURKA* and pro‐tumorigenic TFs (e.g., *ETS2*, *SOX9*, *CEBPB*, and *SNAI2*) to an extent similar to combined inhibition (Figure 4G). Consequently, A1874 induced stronger suppression of phospho‐Histone H3 and key oncogenic proteins than JQ1 or Idasanutlin monotherapy, achieving efficacy that either matched or exceeded the JQ1/Idasanutlin combination (Figure 4H). These data reinforce the therapeutic advantage of MDM2‐mediated degradation of transcriptional/epigenetic machinery over conventional inhibition.

### 2.6 MDM2‐Recruiting Transcriptional/Epigenetic Machinery Degraders (MDM2‐TEMADs) Confer Convergent Inhibitory Effects on Oncogenic Transcriptional Programs

Furthermore, comparative transcriptomic analysis of SJSA1 cells treated with either dCDK9‐010 or A1874 revealed a strong positive correlation (Figure 4I, R = 0.9), indicating that MDM2‐TEMADs exert convergent downstream effects on the sarcoma transcriptional program. Mechanistically, the two MDM2‐TEMADs exerted similar inhibitory effects on Pol II elongation, as evidenced by increased Pol II pausing indexes within 8 h in SJSA1 cells (Figure 4J). Consistent with previous reports of BRD4‐promoted P‐TEFb chromatin recruitment, BET degradation (by A1874) reduced global CDK9 chromatin occupancy intensity (Figure S4E). In contrast, P‐TEFb depletion by dCDK9‐010 did not globally displace BRD4 (Figure S4F). To probe the functional contribution of BRD4 to P‐TEFb function, we designed a rescue assay by introducing an exogenous BRD4‐Cs variant (residues 1192–1362) that retains the ability to interact with P‐TEFb and drive Pol II pause release [32]. Importantly, the BRD4‐Cs variant lacks the N‐terminal BD regions, making it an ideal tool to rule out the intrinsic kinase activity associated with BRD4 BD2 and to evade recognition and destruction by A1874 or the BRD4‐selective PROTAC ZXH‐3‐26 [11, 33, 34]. Selective depletion of BRD4 by ZXH‐3‐26, or concurrent depletion of BET proteins by A1874, consistently reduced phospho‐Rpb1 CTD^Ser2^ (Figure 4K). Strikingly, expression of BRD4‐Cs conferred resistance of phospho‐Rpb1 CTD^Ser2^ to both ZXH‐3‐26 and A1874. However, BRD4‐Cs had no impact on dCDK9‐010 activity, confirming that P‐TEFb acts downstream of BRD4. These observations establish that the BRD4 C‐terminus is critically involved in Pol II Rpb1 CTD^Ser2^ phosphorylation targeted by BET protein degraders. Hence, MDM2‐TEMADs exert convergent inhibitory effects on Pol II elongation and transcription by disrupting the BRD4/P‐TEFb‐mediated phosphorylation of Pol II Rpb1 CTD^Ser2^.

To identify shared downstream transcriptional responses to MDM2‐TEMADs, we performed GSEA and found that both dCDK9‐010 and A1874 consistently activated pathways related to myogenesis, fatty acid metabolism, and oxidative phosphorylation (Figure S4G). Conversely, the MDM2‐associated cistrome showed consistent negative enrichment following treatment with either dCDK9‐010 or A1874, but not with inhibitor monotherapies (Figure 4L–O). Notably, SE‐driven, MDM2^+^ promoter targets showed a lower normalized enrichment score (NES) with dCDK9‐010 than with A1874 treatment (Figure 4L), suggesting enhanced sensitivity of this gene cluster to P‐TEFb degradation. Additionally, SE‐driven, MDM2^−^ promoter targets responded to MDM2‐TEMADs and JQ1, but not SNS032 (Figure 4O). Together, these findings indicate that MDM2‐TEMADs trigger a common transcriptomic remodeling mechanism by coordinately collapsing MDM2‐ and SE‐associated oncogenic transcriptional addiction and activating differentiation and metabolic reprogramming pathways.

### 2.7 MDM2‐TEMADs Impair Homologous Recombination Repair Pathway

Complementing the GSEA results, we performed clustering analysis of transcriptomic profiles based on gene expression patterns across inhibitor and degrader treatments. This identified a distinct gene cluster (Cluster 2) that was hyper‐sensitive to both JQ1/Idasanutlin combination treatment and MDM2‐TEMADs (Figure S4H,I). Ontological analysis revealed significant overrepresentation of biological processes including DNA replication, RNA processing, mitosis, and chromatin/transcription regulation within Cluster 2 (Figure S4J, and Table S8). The cluster contained several validated MDM2^+^ promoter targets (e.g., *ETS2*, *SOX9*, *SNAI2*, and *AURKA*) as well as prominent DNA damage response and repair genes (e.g., *BRCA1*, *PALB2*, and *RFWD3*). These data suggest that MDM2‐TEMADs repress these genes through a compound mechanism involving transcriptional interference and p53 activation.

**FIGURE 5:**
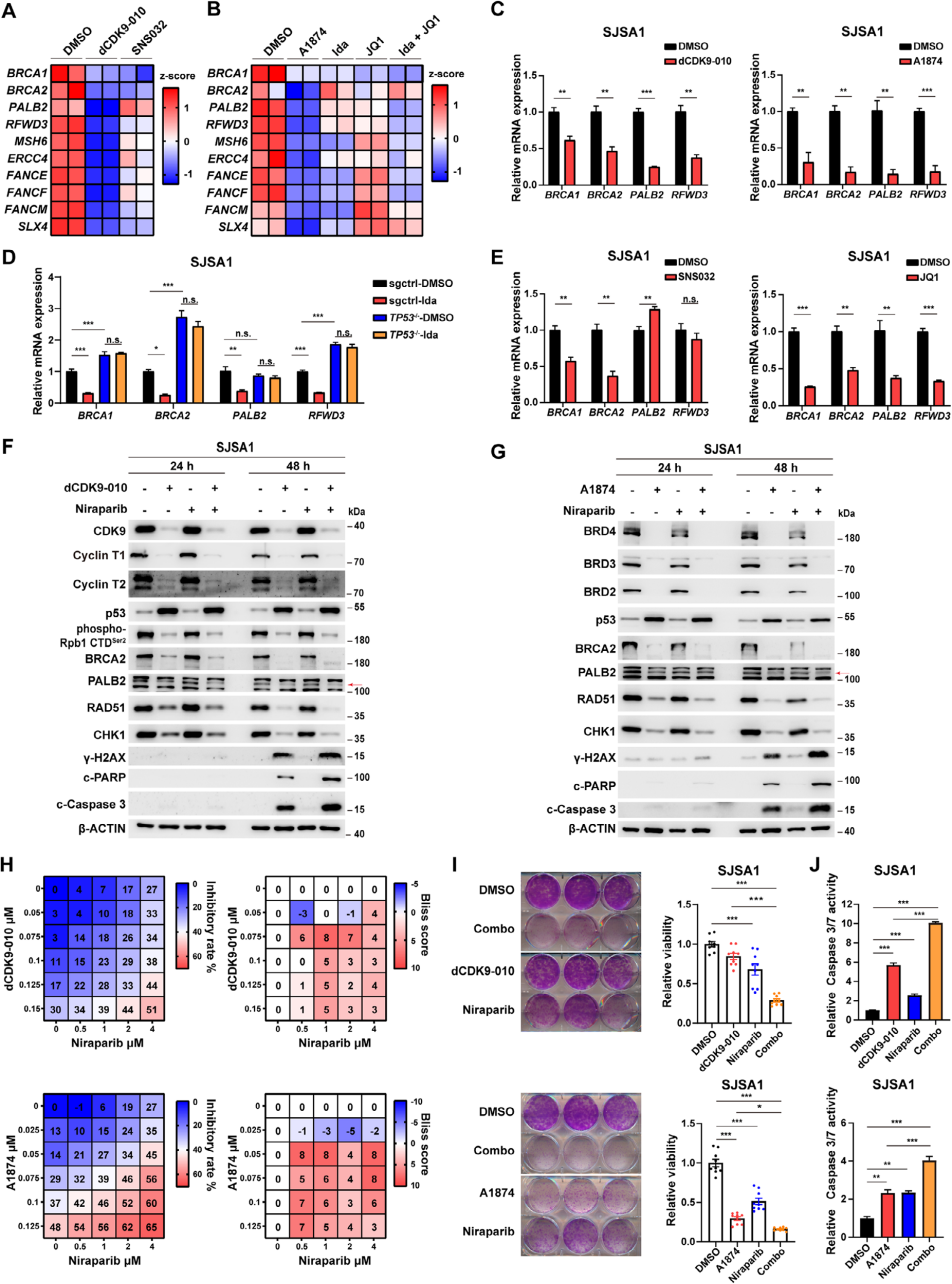
MDM2‐TEMADs impair homologous recombination gene expression and sensitize sarcoma cells to Niraparib. (A, B) Heatmaps showing the expression of DNA damage repair genes including key homologous recombination (HR) genes in response to various drug treatments in RNA‐seq datasets (SJSA1, 500 nm, 8 h). (C) qPCR analysis of 4 HR genes in response to dCDK9‐010 or A1874 treatments (500 nm, 8 h) in SJSA1 cells. (D) Effects of p53 activation by Idasanutlin (500 nm, 8 h) or *TP53* knockout on mRNA levels of 4 HR genes in SJSA1 cells. (E) qPCR analysis of 4 HR genes in response SNS032 or JQ1 treatments (500 nm, 8 h) in SJSA1 cells. (F, G) Effects of MDM2‐TEMADs (500 nm), Niraparib (5 µm), and their combination on downstream protein expression in SJSA cells. The red arrow indicates the target protein band. (H) Effects of MDM2‐TEMADs and Niraparib combination on SJSA1 cell viability. Positive Bliss Score indicates a synergistic effect. (I) Effects of MDM2‐TEMADs (dCDK9‐010, 75 nm; A1874, 100 nm), Niraparib (500 nm), and their combination on SJSA1 survival in foci formation assays. Cells were stained with crystal violet, solubilized, and subjected to absorbance measurement for quantification. (J) Induction of intracellular Caspase 3/7 activity by MDM2‐TEMADs (500 nm, 8 h), Niraparib (5 µm, 8 h), and their combination. Data of (C–E, I, J) are presented as mean±SEM; (C–E, J), n = 3; (I), n = 9. Statistics: (C, E) Student's t‐test (two‐tailed), (D, I, J) one‐way ANOVA. n.s., not significant; *p < 0.05; **p < 0.01; ***p < 0.001.

Aligning with the Cluster 2 findings, analysis of commonly repressed genes following dCDK9‐010 and A1874 treatment revealed profound suppression of DNA damage repair pathways, particularly key homologous recombination (HR) components including *BRCA1*, *BRCA2*, *FANCM*, *PALB2*, and *RFWD3* (Figure 5A,B). qPCR independently validated the downregulation of *BRCA1*, *BRCA2*, *PALB2*, and *RFWD3* (Figure 5C), indicating that MDM2‐TEMADs induce a homologous recombination deficiency (HRD) phenotype resembling HRD*ness* in HR‐proficient sarcoma cells [35]. Notably, Idasanutlin alone suppressed *BRCA2*, *PALB2*, and *RFWD3* transcription in a p53‐dependent manner, as *TP53* knockout abolished this effect (Figure 5D, Figure S5A). While JQ1 similarly reduced expression of these HR genes, SNS032 only attenuated *BRCA1* and *BRCA2* (Figure 5E). Hence, the superior suppression of HR genes by MDM2‐TEMADs stems from their dual capacity to activate the p53 pathway while simultaneously inactivating transcriptional/epigenetic machinery.

Given the established synthetic lethality between HRD and PARP inhibition, we investigated the synergy between MDM2‐TEMADs and the PARP inhibitor Niraparib. Indeed, while MDM2‐TEMADs alone impaired key DNA damage repair proteins including BRCA2, PALB2, RAD51, and CHK1, their combination with Niraparib further exacerbated these defects, leading to progressive accumulation of γ‐H2AX (indicator of DNA damage) and elevated levels of cleaved‐PARP and cleaved‐Caspase 3 (markers of apoptosis) (Figure 5F,G, Figure S5B,C). Consequently, both dCDK9‐010 and A1874 synergized with Niraparib, reducing the viability, suppressing clonogenic survival, and enhancing Caspase 3/7 activity in pediatric sarcoma cells (Figure 5H–J). These results demonstrate that pharmacologic induction of HRD*ness* by MDM2‐TEMADs sensitizes pediatric sarcoma to PARP inhibition.

### 2.8 MDM2‐TEMADs Exhibit Selective Anti‐Sarcoma Efficacy and Favorable In Vivo Tolerability

**FIGURE 6:**
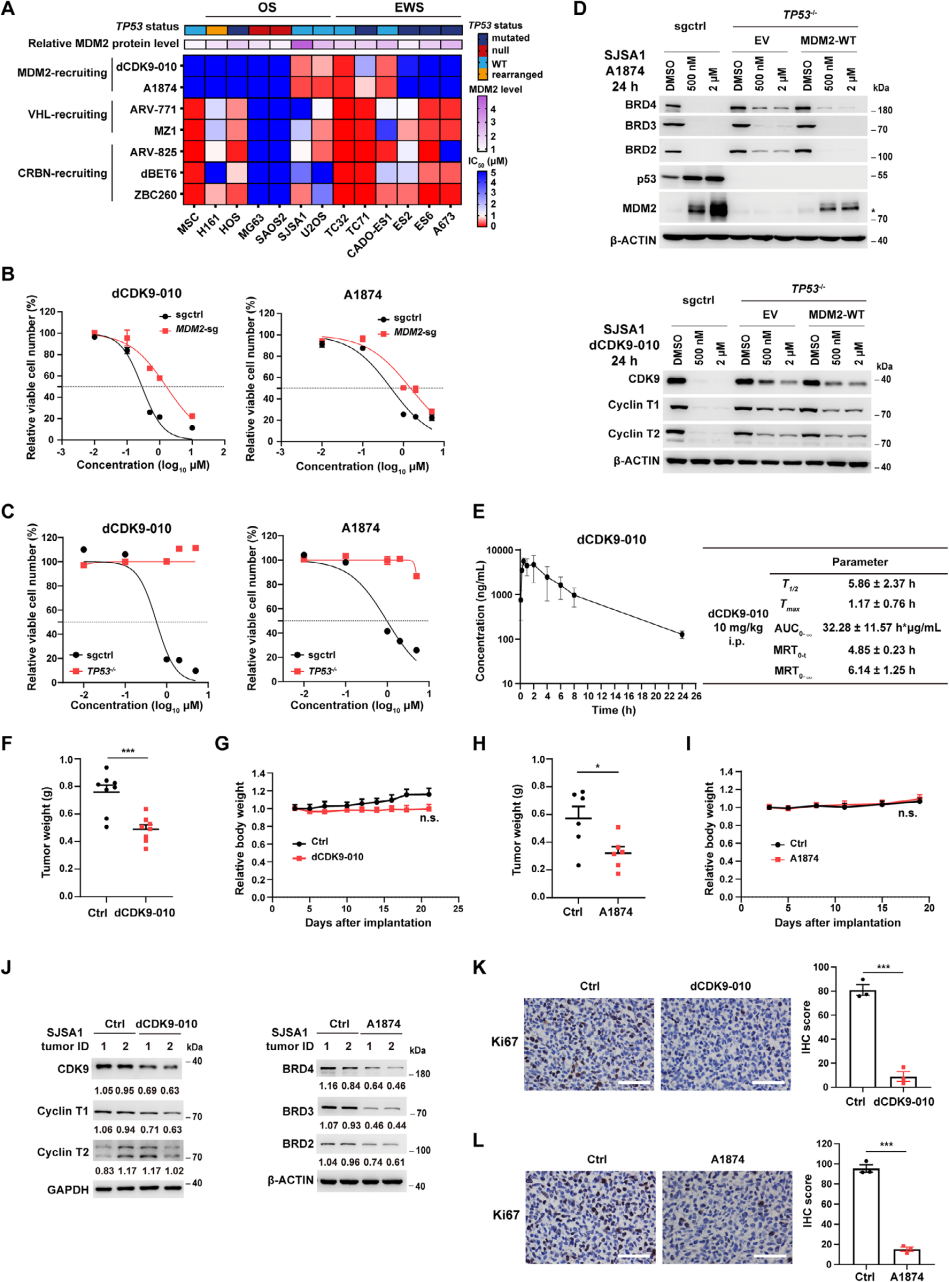
MDM2‐TEMADs exhibit selective anti‐sarcoma activities. (A) Heatmap visualization of the IC_50_ values of dCDK9‐010 and various BET protein degraders across a panel of MSC, EWS, and OS cell lines. Relative MDM2 level was derived from Figure 1B. WT, wildtype; null, *TP53* deletion. (B, C) Effect of *MDM2* and *TP53* silencing on the response of SJSA1 cells to MDM2‐TEMADs. (D) Effect of TP53 knockout and MDM2 restoration on the target degradation efficacy of dCDK9‐010 and A1874 in SJSA1 cells. (E) Pharmacokinetics of dCDK9‐010 in mice following a single intraperitoneal injection. (F, G) Effect of dCDK9‐010 (20 mg/kg, i.p., qod) on (F) the subcutaneous tumor growth of SJSA1 cells in immunocompromised mice and (G) mouse body weight. (H, I) Effect of A1874 (20 mg/kg, i.v., qod) on (H) the subcutaneous tumor growth of SJSA1 cells in immunocompromised mice and (I) mouse body weight. (J) Expression levels of the indicated proteins in tumor lysates collected at the end of in vivo efficacy study. (K, L) Immunohistochemistry (IHC) analysis of Ki67 expression in xenografts harvested at the experimental endpoint. Scale bars represent 50 µm. Data of (B, C, F–I, K, L) are presented as mean±SEM; data of (E) are presented as mean±SD; (B, C, E, I, K, L), n = 3; (F), n = 8; (G), n = 4; (H), n = 6. Statistics: (F–I, K, L) Student's t‐test (two‐tailed). n.s., not significant; *p < 0.05; ***p < 0.001.

To assess the translational potentials of MDM2‐TEMADs, we evaluated their anti‐proliferative efficacy alongside various VHL‐ and CRBN‐recruiting BET protein degraders across a panel of OS, EWS, and MSC cells (Figure 6A). While MSCs and most sarcoma cells showed similar sensitivity to VHL‐ and CRBN‐based BET PROTACs, MDM2‐TEMADs elicited potent and selective cytotoxicity in a subset of OS (SJSA1, U2OS) and EWS (TC32, CADO‐ES1) cell lines, sparing MSCs. Further examination of cellular responsiveness to CRBN‐recruiting BET or CDK9 PROTACs indicated that transcriptional machinery degradation alone is cytotoxic, regardless of cellular *MDM2* and *TP53* status (Figure S6A). These data highlight the targeted nature of MDM2‐TEMADs and their potential for a wider therapeutic window. Remarkably, the selectivity of MDM2‐TEMADs was associated with high MDM2 expression and wildtype *TP53* status in sensitive cells (Figure 6A). In line with this, an MDM2‐overexpressing primary OS explant line ZCH002M also showed desirable responses to MDM2‐TEMADs (Figure S6B). Importantly, CRISPR/Cas9‐mediated *MDM2* silencing in SJSA1 cells attenuated their efficacy (Figure 6B, Figure S6C), confirming an MDM2‐dependent mechanism of action. *TP53* knockout in SJSA1 cells severely impaired the target degradation capacity of MDM2‐TEMADs and abolished treatment‐induced MDM2 upregulation, leading to attenuated cellular responsiveness (Figure 6C,D). Re‐introducing exogenous MDM2 into *TP53*‐deficient SJSA1 cells can restore the target degradation capacity of A1874 and partially restored the CDK9/Cyclin T1 degradation ability of dCDK9‐010 (Figure 6D). These data indicate that the functional dependency of MDM2‐TEMADs on p53 is primarily mediated through p53‐driven MDM2 expression and its further induction upon treatment.

The selective in vitro efficacy of MDM2‐TEMADs against MDM2‐driven pediatric sarcoma cells prompted an evaluation of their in vivo antitumor potential. Pharmacokinetic (PK) analysis in mice following a single intraperitoneal dose of dCDK9‐010 (10 mg/kg) revealed favorable parameters for in vivo application, including a plasma half‐life (T_1/2_) of 5.86 ± 2.37 h, time to reach maximum concentration (T_max_) of 1.17 ± 0.76 h, and an area under the curve (AUC_0‐∞_) of 32.3 ± 11.6 h·µg/mL (Figure 6E). Based on this promising PK profile, we examined the therapeutic efficacy of dCDK9‐010 using an SJSA1 subcutaneous xenograft model. Intraperitoneal injection of dCDK9‐010 (20 mg/kg, qod) significantly retarded xenograft growth without causing body weight loss (Figure 6F,G, Figure S6D). A1874 (20 mg/kg, i.v., qod) produced similar antitumor efficacy (Figure 6H,I, Figure S6E). In line with the intratumoral target degradation in excised tumors, subsequent histopathological analysis of corresponding xenografts further confirmed that both dCDK9‐010 and A1874 treatments strongly reduced Ki67 staining intensity and positive cell frequency (Figure 6J–L), indicating potent suppression of sarcoma cell proliferation in vivo. Finally, we assessed the in vivo safety profiles of dCDK9‐010 and A1874. NCG mice received A1874 (20 mg/kg, i.v.) or dCDK9‐010 (20 mg/kg, i.p.) following the same schedule as the in vivo efficacy experiment for three weeks. At the end of treatment, vital organs (heart, liver, spleen, lung, and kidney) were collected and examined for drug‐induced injury. No obvious morphological or structural changes were observed in any of these organs (Figure S6F,G), indicating a low risk of subacute toxicity. Together, these findings demonstrate promising anti‐sarcoma efficacy and excellent tolerability of MDM2‐TEMADs in vivo, encouraging their further translational development.

## 3 Discussion

**FIGURE 7:**
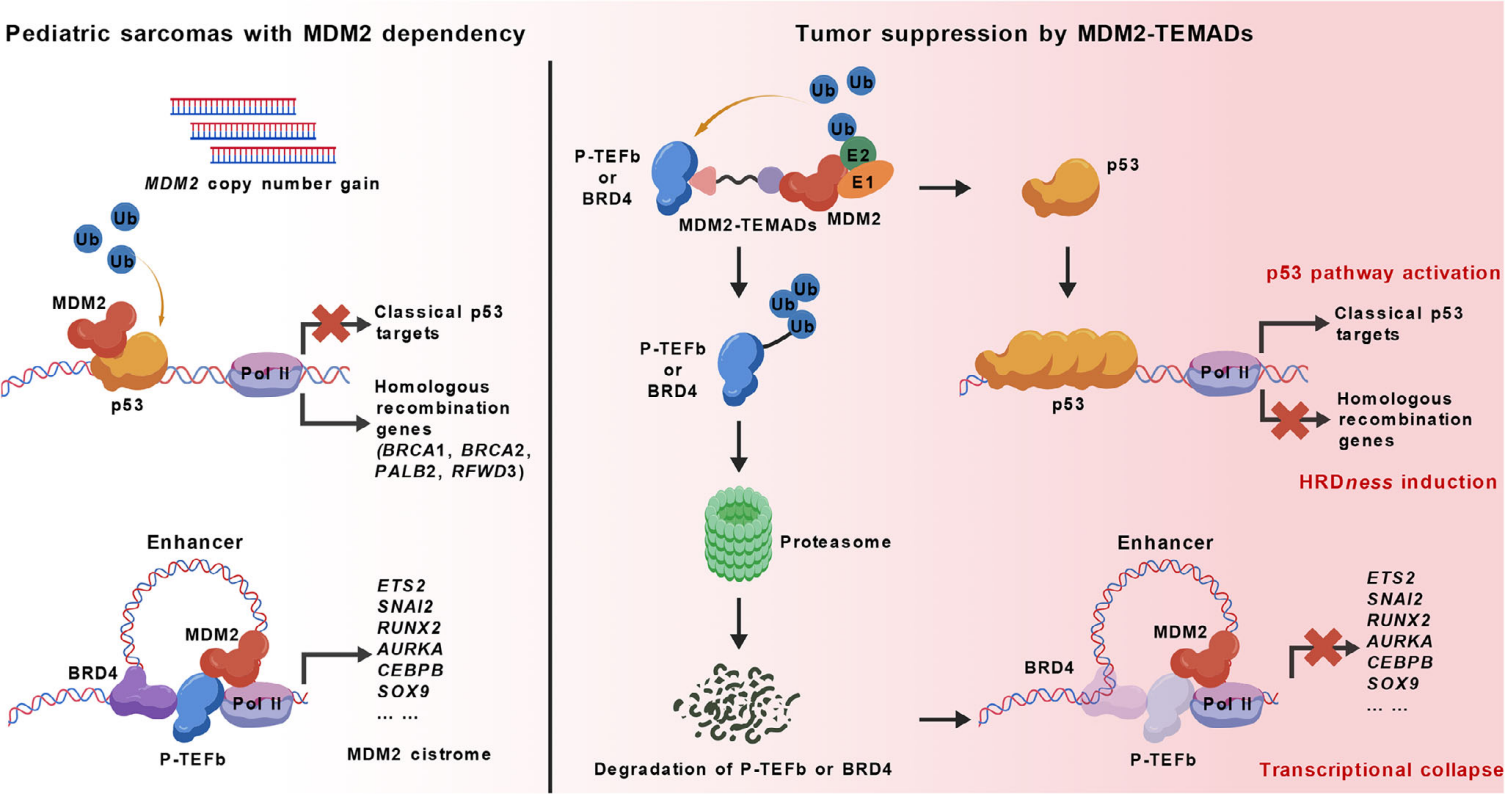
Schematic model depicting MDM2 dependency in pediatric sarcomas and the therapeutic rationale for MDM2‐TEMADs.

In this study, we reveal a critical role of MDM2 in configuring sarcoma‐promoting transcriptome through both canonical p53 suppression and its newly characterized p53‐independent cistrome (Figure 7). To target this oncogenic MDM2 dependency, we introduce a novel strategy employing MDM2‐TEMADs. This strategy is exemplified by the newly developed P‐TEFb degrader dCDK9‐010 and further validated by the BET degrader A1874. These MDM2‐TEMADs demonstrate a convergent mechanism to simultaneously disrupt MDM2 cistrome and enhancer‐driven gene expression while activating p53 and inducing HRD. Leveraging sarcoma's dependency on aberrantly expressed MDM2, MDM2‐TEMADs represent a promising class of targeted therapeutics with favorable selectivity and tolerability, and potent anti‐sarcoma efficacy in preclinical models.

During tumorigenesis, genetic and epigenetic alterations invariably reprogram transcriptional networks, creating dependencies that sustain malignant identity and phenotypes. Elucidating and therapeutically targeting these dependencies provides a basis for innovative cancer treatment. In this study, we reveal frequent *MDM2* copy number gains or amplifications in OS and EWS, and functionally establish MDM2 as a common genetic dependency operating through two primary mechanisms: (1) canonical suppression of p53 and its pathway, and (2) p53‐independent chromatin occupancy that regulates transcription of MDM2 cistrome. Interestingly, MDM2‐bound chromatin in pediatric sarcoma cells is enriched for active histone marks and transcriptional activators (e.g., ETS2, AP‐1, YY1). This pattern is consistent with a recent report in adult liposarcoma but contrasts with an earlier study showing that MDM2 enhanced H3K27me3 deposition via interaction with the polycomb repressor complex 2 in murine embryonic fibroblasts [28, 36]. It is likely that genetic background and sarcoma‐specific co‐factors may shape the genomic distribution and functional cistrome of MDM2. Although this study identifies a role for ETS2 in maintaining total and chromatin‐bound MDM2, additional investigations are warranted to fully elucidate the functional crosstalk between MDM2 and ETS2 and to uncover other factors governing MDM2 chromatin recruitment. Importantly, our analysis further reveals that MDM2 preferentially occupies enhancers and active promoters of prominent oncogenes and mitotic genes, rather than p53 binding sites, highlighting a p53‐independent role of MDM2 in driving oncogenic transcription. Further supporting a p53‐independent pro‐tumorigenic function for MDM2, its depletion has been shown to kill p53‐inactivated triple‐negative breast cancer cells [37]. Therefore, effective targeting of MDM2‐driven pediatric sarcoma requires concurrent disruption of both the MDM2‐p53 axis and MDM2's p53‐independent oncogenic functions. Furthermore, consistent with previous reports [38, 39], MDM2 expresses multiple isoforms that exhibit differential p53 dependency and treatment responsiveness in sarcoma cells. The isoform‐specific expression regulation and functions in sarcomagenesis remain to be further elucidated.

Although targeting transcriptional regulators like CDK9 can disrupt oncogenic transcription, the clinical utility of conventional inhibitors (e.g., CDK9 inhibitors; Figure S7) is limited by poor selectivity. Similarly, current CDK9 degraders (e.g., CRBN‐recruiting PROTACs [18, 19, 40], autophagy‐tethering compound [41], and hydrophobic tags [42, 43, 44]) also lack tumor‐cell selectivity (Figure S7). To address this limitation, we developed MDM2‐recruiting PROTACs that hijack tumor‐specific MDM2 to selectively degrade all P‐TEFb components (CDK9, Cyclin T1, and Cyclin T2). The rationale behind this design lies in the heterobifunctional compound‐induced proximity of target proteins, through which SNS032 and Idasanutlin analogs recruit CDK9 (together with Cyclin T) and the MDM2 E3 ligase complex to form a ternary complex permissive for ubiquitin conjugation. Although the relative expression levels and stability of two Cyclin T2 isoforms appear context‐dependent, varying among cell lines and treatment conditions, both isoforms can be effectively depleted by MDM2‐recruiting PROTACs. Our lead compound, dCDK9‐010, exerts potent, MDM2‐dependent anti‐sarcoma efficacy with a superior therapeutic index. dCDK9‐010 undermines oncogenic transcription through multiple convergent mechanisms: (1) disrupting enhancer‐driven transcriptional network; (2) inhibiting the p53‐independent MDM2 cistrome (especially MDM2^+^ promoter targets); (3) suppressing Rpb1 activity and expression; (4) activating p53, and (5) inducing DNA damage repair deficiency (HRD*ness*). Intriguingly, these effects were recapitulated by an MDM2‐recruiting BET degrader (A1874), revealing a shared pharmacological mechanism for MDM2‐dependent degradation of transcriptional/epigenetic machinery and highlighting the essential collaboration between BRD4 and P‐TEFb in driving oncogenic transcription. Indeed, the BRD4 C‐terminus alone is sufficient to counteract the inhibitory effect of BET degradation on P‐TEFb‐driven Pol II Rpb1 CTD^Ser2^ phosphorylation. Thus, in addition to their shared ability to activate p53, dCDK9‐010 and A1874 converge to impair BRD4/P‐TEFb‐mediated Pol II phosphorylation and transcription elongation. We term this class of compounds “MDM2‐TEMADs” and provide a compelling rationale for their clinical translation.

Both MDM2 expression and *TP53* status serve as crucial biomarkers for this strategy, with MDM2‐high, *TP53*‐wildtype tumors representing the primary target population. MDM2‐TEMADs exhibit desirable cell selectivity, unlike CRBN‐recruiting transcriptional machinery degraders whose cytotoxicity was independent of *MDM2* and *TP53* status (Figure S6A). *TP53*‐null cells (e.g., SJSA1*^TP53^* ^‐/−^ and a frequently used *TP53* knockout colon cancer cell line model HCT116*^TP53^* ^‐/−^) remained sensitive to CRBN‐recruiting transcriptional machinery degraders, except for a slight decrease of ZBC260 sensitivity in SJSA1*^TP53^* ^−/Ȓ^ cells. Hence, building upon effective transcriptional machinery degradation that generally elicits cytotoxicity, MDM2‐TEMADs have the merits to exert selective cytotoxicity against cancer cells with proficient *MDM2* and *TP53*. Interestingly, A1874 and dCDK9‐010 exhibited slightly different dependencies on MDM2 and p53 availability. In the absence of functional p53, MDM2 overexpression conferred A1874 target degradation capability, whereas it only partially restored dCDK9‐010 activity. It is likely that full activity of dCDK9‐010 requires the presence of wildtype p53 and additional cooperating factors. Of note, MSCs exhibit reduced sensitivity to these MDM2‐TEMADs, primarily due to their low basal MDM2 expression combined with intrinsic tolerance of p53 activation. Supporting this notion, MDM2 overexpression in MSCs enhanced the target degradation efficacy of MDM2‐TEMADs, and MSCs also showed greater tolerance to conventional MDM2 inhibitor (Figure S8). Additionally, based on the drug sensitivity profiles of MG63, SAOS2, and H161 cells, *TP53*‐null sarcomas appear unsuitable for BRD4‐ or P‐TEFb‐targeted therapies. Future efforts will focus on advancing MDM2‐TEMADs toward clinical trials.

MDM2‐TEMADs transcriptionally reconfigure sarcoma cells from HR‐proficient to HR‐deficient states by preferentially suppressing HR‐related gene expression. This effect is mediated, at least partially, through MDM2‐TEMAD‐dependent transcriptional interference and p53 activation. Additionally, dCDK9‐010‐mediated degradation of the long CDK9 isoform may further impair HR function by inhibiting CDC23 phosphorylation [45]. Consistent with the robust suppression of HR genes (e.g., *BRCA2*, *PALB2*), we observed synthetic lethality between MDM2‐TEMADs and PARP inhibitors in MDM2‐driven sarcoma cells. This finding aligns with a previous report showing encouraging antitumor efficacy through sequential inhibition of PARP and BET proteins [46]. The in vivo efficacy and translational potential of combining MDM2‐TEMADs with PARP inhibitors warrant further investigation.

Taken together, our findings identify oncogenic transcriptional addiction to MDM2 as a critical therapeutic vulnerability in pediatric sarcoma, establishing the rationale for targeting this dependency with MDM2‐recruiting degraders of transcriptional/epigenetic machinery.

## 4 Experimental Section/Methods

### 4.1 Cell Culture

All cell lines (Table S9) tested negative for mycoplasma and are not listed in the ICLAC database. Human embryonic kidney cells 293T (HEK293T, ATCC), HCT116 (ATCC), TC32 (COG Repository), TC71 (DSMZ), CADO‐ES1 (DSMZ), A673 (ATCC), HTLA161 (H161, kindly provided by Dr. Emil Bogenmann), MNNG/HOS Cl #5 (HOS, ATCC), MG63 (ATCC), SAOS2 (ATCC), SJSA1 (ATCC), U2OS (ATCC), and two additional EWS cell lines ES2 and ES6 (kindly provided by Dr. Peter Houghton) were cultured in Dulbecco's Modified Eagle Medium (DMEM, Meilunbio). MDM2‐overexpressing primary OS explant line ZCH002M was established from a consented donor under the ethical approval of the Ethics Committee of Children's Hospital, Zhejiang University School of Medicine (Protocol No. 2024‐IRB‐0191), and was maintained in DMEM with GlutaMAX. All aforementioned cells were supplemented with 10% fetal bovine serum (FBS, Vazyme) and 1% penicillin‐streptomycin (Beyotime). Human mesenchymal stem cells (MSCs) were cultured in ncMission hMSC medium (Nuwacell). All cells were cultured in a humidified incubator with 5% CO_2_ at 37°C. All sarcoma cell lines were authenticated by short tandem repeat analysis.

### 4.2 Plasmids and Chemicals

All lentiviral vector‐based overexpression constructs, pLKO.1‐based short hairpin RNA (shRNA) vectors and lentiCRISPRv2‐based sgRNA vectors were listed in Tables S10 and S11. SHC002 was used as a non‐targeting control (shNT). Stable cell lines were generated by lentiviral transduction followed by selection with puromycin or blasticidin S. Chemical synthesis of the Idasanutlin‐SNS032 conjugates was performed as described in the Supporting Information Note. The sources of A1874, SNS032, Idasanutlin, JQ1, and other commercially available reagents used in this study are listed in Table S12.

### 4.3 Cell Viability Assay

Cell viability was measured by an MTT (3‐(4,5‐dimethylthiazol‐2‐yl)‐2,5‐diphenylte‐trazolium bromide) colorimetric assay [47]. Depending on the proliferative potentials of each cell line, cells were seeded in 96‐well plates at densities ranging from 1200 to 5000 cells per well. Then these cells were cultured for 72 h under the specified treatments to assess the dose‐response relationship. MTT substrate (Coolaber) was added to each well and incubated for 3 h before addition of 100 µL of solubilization buffer. The plates were then read on a microplate reader (Molecular Devices) with absorbance measured at 570 nm. SynergyFinder Plus was employed for analyzing dose‐response data derived from the combinations between Idasanutlin and transcriptional machinery inhibitors [48]. Based on ZIP Synergy scores calculated by SynergyFinder, combination effects with a score greater than 10 were interpreted as synergistic, while those between −10 and 10 were defined as additive. Alternatively, the Bliss model was used to evaluate combination effect between Niraparib and MDM2‐TEMADs. Bliss Expectation was calculated using the formula (A + B)−A × B, where A and B represent the growth inhibition rates of Compound A and Compound B at a given dose, respectively.

### 4.4 BrdU Incorporation Assay

Sarcoma cells were seeded into a 96‐well plate at a density of 5000 cells per well. After overnight culture, cells were incubated with indicated compounds for another 24 h, followed by addition of BrdU and incubation at 37°C for 4 h. Cells were then fixed by formaldehyde (PFA). BrdU incorporated by proliferating cells was detected by an enzyme‐linked immunosorbent assay protocol provided with the BrdU Cell Proliferation Assay Kit (BioVision).

### 4.5 Caspase‐Glo Assay

Cells were seeded into a white‐walled 96‐well plate at a density of 3000–10 000 cells per well. After overnight culture to allow cell attachment, cells were treated with the indicated compounds for an additional 24 h. A Caspase‐Glo 3/7 assay (Promega) was conducted according to the manufacturer's instruction, to monitor the cellular Caspase 3/7 activity that serves as an indicator of apoptosis. The luminescence of each sample was detected by a FlexStation 3 multimode microplate reader (Molecular Devices). Culture media without cells were included as a background control.

### 4.6 Focus Formation Assay

Sarcoma cells were seeded in 6‐well plates at a density of 1000 cells per well. Following overnight culture, cells were treated with the indicated compounds and maintained under standard cell culture conditions for 10–12 days. Foci were fixed and stained with 0.01% crystal violet in 4% paraformaldehyde (PFA)/PBS solution for 15 min. After staining, wells were washed and imaged. For quantitative analysis, stained foci were dissolved in 1 mL of MTT stop solution per well, and absorbance was measured at 570 nm using a microplate reader (Molecular Devices).

### 4.7 Soft‐Agar Colony Formation Assay

A basal layer of agar was prepared by mixing 2× DMEM medium (supplemented with 2× penicillin–streptomycin and 20% FBS) with an equal volume of 0.8% agarose (Invitrogen). Then, 500 µL of this mixture was added to each well of a 12‐well plate and solidified at 4°C. For the top layer, about 750–1000 sarcoma cells were suspended in 500 µL of a mixture containing 2× culture medium and 0.8% low‐melting agarose (Bio‐Rad). The cell‐agar mixture was carefully overlaid onto the basal layer. Plates were placed at 4°C for 30 min to ensure complete solidification of the top layer. Then, 1 mL of complete medium was added to each well, and cells were cultured for 2 weeks. Colonies were stained with 0.01% crystal violet in 4% PFA/PBS for 3 h, and imaged using bright‐field microscopy.

### 4.8 Cell Lysis and Immunoblot Assay

Whole‐cell extracts were prepared by incubating cells in lysis buffer (50 mm Tris pH 8.0, 420 mm NaCl, 5% glycerol, 0.1% NP‐40, 0.1 mm EDTA) with fresh addition of 1 mm dithiothreitol, 1 mm phenylmethylsulfonyl fluoride (PMSF), 1× protease inhibitor cocktail, 1× phosphatase inhibitor cocktail, 1 mm MgCl_2_ and Benzonase (1:500, Novoprotein) for 20 min on ice. To extract chromatin‐bound proteins, cell pellet was lysed by CSK buffer (10 mm PIPES, pH 6.8, 300 mm sucrose, 100 mm NaCl, 1 mm MgCl_2_, 1 mM EDTA, 1 mm EGTA, 0.5% Triton X‐100) supplemented with protease and phosphatase inhibitors. After centrifugation, non‐chromatin soluble fraction was enriched in the supernatant. The insoluble pellet was washed twice with CSK buffer, re‐suspended in lysis buffer, sonicated, and digested with Benzonase for 20 min on ice. Clear supernatant after centrifugation (13000 *g*, 10 min) represented the chromatin fraction. Protein concentration was quantified by Bradford assay. Immunoblot analysis was conducted following the standard protocol with the indicated antibodies listed in Table S13.

### 4.9 Co‐Immunoprecipitation

Cells were lysed in immunoprecipitation buffer (50 mm Tris pH 7.6, 150 mm NaCl, 5% glycerol, 0.1% NP‐40, 1 mm EDTA) supplemented with protease and phosphatase inhibitors, 1 mm MgCl_2_, and Benzonuclease. For each immunoprecipitation reaction, 1 mg of cell lysate was incubated with either anti‐FLAG M2 affinity gel (A2220; Sigma‐Aldrich) or HA magnetic beads at 4°C for 2 h with gentle rotation. Following four washes with immunoprecipitation buffer, bound proteins were eluted and subsequently analyzed by sodium dodecyl sulfate (SDS)‐polyacrylamide gel electrophoresis and immunoblotting.

### 4.10 Chromatin Immunoprecipitation (ChIP)

SJSA1 cells were fixed by either 1% formaldehyde (for H3K27ac ChIP) or 2 mm disuccinimidyl glutarate and 1% formaldehyde two‐step cross‐linking strategy (for MDM2 ChIP) at room temperature, followed with three washes using cold PBS. The remaining chromatin immunoprecipitation (ChIP) steps were performed according to a standard protocol. Briefly, nuclei were extracted and resuspended in SDS lysis buffer, followed by incubation on ice for 10 min. Chromatin was sheared using a Bioruptor sonicator (Diagenode) to generate DNA fragments ranging from 200–500 bp. The sonicated chromatin supernatant was then incubated overnight with Dynabeads Protein A/G (Invitrogen), which had been pre‐conjugated with target‐specific antibodies (Table S13). After incubation, the beads were sequentially washed with cold low‐salt buffer, high‐salt buffer, LiCl buffer, and TE buffer. Bound chromatin complexes were eluted and reverse‐cross‐linked, and DNA was purified using the QIAquick PCR Purification Kit (Qiagen). Libraries for ChIP‐seq were prepared with the ThruPLEX DNA‐seq Kit (Takara) and sequenced on an Illumina platform.

### 4.11 ChIP‐Seq Analysis

For ChIP‐seq data analysis, raw sequencing reads were aligned to the human reference genome (UCSC hg38) using BWA‐MEM (v0.7.17‐r1188) (https://github.com/lh3/bwa.git) under default parameters. PCR duplicates were marked and removed using Picard MarkDuplicates (v2.27.4; –REMOVE_DUPLICATES true) to generate final, cleaned BAM files for downstream analysis. Peak calling was performed using MACS2 (v2.2.7.1, –nomodel). The predicted fragment size for read extension was determined by strand cross‐correlation analysis using phantompeakqualtools (v2.0). MACS2 was also used to generate input‐subtracted ChIP signal tracks (bedGraph) via the bdgcmp function, which were subsequently converted to bigWig format using the UCSC bedGraphTobigWig utility. Peaks were annotated to genomic features using HOMER (v4.11, annotatePeaks). SEs were modeled using the ROSE algorithm (https://bitbucket.org/young_computation/rose). Regions within ±1250 bp of a transcription start site were excluded from the SE analysis. Input‐subtracted ChIP‐seq signals from constituent enhancers were stitched together, ranked by signal intensity, and classified as SEs based on a geometric inflection point in the rank‐order plot. Motif enrichment analysis was performed using HOMER. Data visualization was conducted with Integrative Genomics Viewer (v2.14.0), and heatmaps were generated using DeepTools (v3.5.1). Statistical plots were created with ggplot2 (v3.4.4) in R.

### 4.12 Cleavage Under Targets and Tagmentation (CUT&Tag)

The CUT&Tag assay was conducted to profile genome‐wide binding sites of target proteins in SJSA1 cells, following the manufacturer's instructions for the NovoNGS CUT&Tag 4.0 High‐Sensitivity Kit (N259‐YH01; Novoprotein). In brief, 100 000 cells were cross‐linked with formaldehyde, and nuclei were subsequently isolated. The isolated nuclei were then immobilized using Concanavalin A‐coated magnetic beads. After permeabilization, the samples were sequentially incubated with target‐specific primary antibodies (Total‐Rpb1, Cell Signaling Technology; HA, Cell Signaling Technology), a secondary antibody, and pAG‐Tn5 fusion proteins. Tagmentation was initiated by adding MgCl_2_ to generate adaptor‐ligated DNA fragments. The resulting DNA fragments were then purified, amplified by PCR, and sequenced on an Illumina platform.

### 4.13 CUT&Tag‐Seq Analysis

Paired‐end reads were aligned to the UCSC hg38 reference genome and a synthetic spike‐in sequence using Bowtie2 (v2.3.5.1; –very‐sensitive –no‐unal –no‐mixed –no‐discordant ‐I 10 ‐X 700). SAMtools (v1.16.1) was utilized to filter alignments, retaining only high‐quality, uniquely mapped reads (MAPQ≥30). For absolute quantification, a sample‐specific scale factor was computed as 10^6^ divided by the total count of properly paired, uniquely mapped spike‐in fragments. Genomic binding sites were identified using MACS2 (v2.2.7.1; ‐f BAMPE ‐q 0.01 –SPMR –keep‐dup all), followed by the removal of ENCODE blacklist regions via bedtools. Finally, absolute spike‐in normalized signal tracks were generated with deepTools bamCoverage (v3.5.1; –extendReads –binSize 10 –normalizeUsing None) by directly applying the pre‐calculated scale factors to avoid double‐normalization artifacts.

### 4.14 RNA Extraction, qRT‐PCR, and RNA‐Seq

Total RNA from cell samples was extracted using either FastPure Cell/tissue total RNA isolatoin kit (Vazyme) according to the manufacturer's instructions. RNA samples were treated with DNase and subjected to reverse transcription using HiScript III first Strand cDNA Synthesis Kit (Vazyme). qPCR was performed using ChamQ Universal SYBR qPCR Master Mix (Vazyme) on a CFX Opus 96 Real‐Time PCR System (Bio‐Rad). qPCR primers are listed in Table S14. For RNA‐seq, RNA integrity was confirmed by a Qsep 100 (BIOptic). Stranded mRNA library preparation and paired‐end RNA sequencing on a Novaseq 6000 sequencer were conducted by Seqhealth TechnologyCo., Ltd.

### 4.15 RNA‐Seq Analysis

The quality of reads was assessed with FastQC (v0.12.1) and MultiQC (v1.15). Paired‐end reads were aligned and quantified to Ensembl (GRCh38) transcripts using STAR (v2.7.11b) and RSEM (v1.3.3) program. Differential gene expression analysis was performed using DESeq2 (v1.44.0). GSEA was performed on all active genes using R packages clusterProfiler and fGSEA. C‐means‐based clustering of the transcriptomes was conducted using the R package Mfuzz (v2.56.0).

### 4.16 Animal Studies

All animal experiments were conducted in accordance with ethical approval from Zhejiang University Institutional Animal Care and Use Committees (IACUC Protocol ID: ZJU20210146, ZJU20241058). For the pharmacokinetic studies, 6‐week‐old CD‐1 (ICR) mice were intraperitoneally administrated with dCDK9‐010 (10 mg/kg). Approximately 50 µL of blood was collected from the cheek vein of each mouse at 5, 15, and 30 min, and at 1, 2, 4, 6, 8, and 24 h post‐injection. Plasma concentrations of dCDK9‐010 were quantified using liquid chromatography‐tandem mass spectrometry, and pharmacokinetic parameters were derived through non‐compartmental analysis with WinNonlin software. For subcutaneous xenograft studies, 6‐ to 8‐week‐old sex‐matched NCG mice (NOD/ShiLtJGpt‐*Prkdc^em26Cd52^Il2rg^em26Cd22^*/Gpt; GemPharmatech) were used as recipients. Human sarcoma cells were resuspended in 100 µL of Matrigel/PBS mixture and inoculated dorsally into shaved mice. For in vivo drug treatment, mice bearing palpable SJSA1 xenografts were randomized into two groups. Mice in experimental arms received dCDK9‐010 (20 mg/kg, i.p., qod) or A1874 (20 mg/kg, i.v., qod), while animals in the control arm received vehicle (5% Solutol HS‐15 in PBS, same volume and schedule). Tumor size was measured by caliper and tumor volume (mm^3^) was estimated according to formula 1/2(Length×Width^2^). At endpoint, tumors were dissected and weighed. No specific randomization method or blinding was applied.

### 4.17 Hematoxylin and Eosin (HE) Staining

Tissues dissected from mice were fixed in 4% formaldehyde for 24 h. Samples were then embedded in paraffin and sectioned at 5 µm thickness onto adhesive slides. After deparaffinization and rehydration through a graded ethanol series, tissue sections were stained with hematoxylin for 30 sec at room temperature, followed by a 15‐min rinse under running tap water. Sections were subsequently counterstained with eosin for 30 sec and rinsed again. Finally, sections were mounted with neutral mounting medium and imaged using bright‐field microscopy.

### 4.18 Immunohistochemical (IHC) Staining

Paraffin‐embedded tumors sections were deparaffinized in xylene and rehydrated through a graded ethanol series. Endogenous peroxidase activity was quenched by incubation in 3% hydrogen peroxide in methanol for 15 min. After antigen retrieval (citrate buffer, pH 6.0), sections were incubated with a primary antibody against Ki67 (1:50 dilution; DAKO, #M7240) overnight at 4°C, followed by incubation with an anti‐mouse IgG, horseradish peroxidase‐linked secondary antibody. Detection was performed using a DAB substrate chromogen system. Finally, sections were counterstained with hematoxylin (Beyotime), and mounted. Images were acquired using a high‐resolution digital slide scanner. The IHC score was calculated based on the proportion of positively stained nuclei per field using the formula: IHC score = (% Low Positive × 1 + % medium Positive × 2 + % High Positive × 3) × 100.

### 4.19 Molecular Docking

The 3D structures of the SNS032 (CID: 3025986) and Idasanutlin (CID: 53358942) were downloaded from the PubChem database. The crystal structures of CDK9 (PDB ID: 8K5R) and the p53‐binding domain of MDM2 (PDB ID: 4JRG) were retrieved from the Protein Data Bank (https://www.rcsb.org) [49, 50]. Structural preprocessing, including (1) removal of crystallographic water molecules, (2) elimination of native ligands, and (3) addition of polar hydrogen atoms was performed using PyMOL open source (v3.1.0). Binding pockets were characterized using the GetBox plugin (v20180204) in PyMOL, and spatial coordinates of the pockets were obtained. Molecular docking simulations were performed using AutoDock‐GPU (v1.6). The resulting complexes were processed for bond correction using Open Babel (v3.1.1). Protein surface electrostatic potentials were computed with APBS (v3.4.1), and structural visualization was performed using PyMOL.

### 4.20 Proteomic Analysis

SJSA1 cells were treated with DMSO or dCDK9‐010 (500 nm, 8 h) prior to harvest. Samples were lysed with DB lysis buffer (6 m urea, 100 mm triethylammonium bicarbonate buffer, pH 8.5), followed by 5 min of ultrasonication on ice. Lysates were centrifuged at 12 000 g for 15 min at 4°C; 1 m dithiothreitol was added to the supernatant and incubated at 56°C for 1 h, then alkylated with sufficient iodoacetamide for 1 h at room temperature in the dark, followed by 2‐min ice‐bath. Protein digestion and analysis were performed by Novogene Co., Ltd. (Beijing, China) using a Vanquish Neo UHPLC‐Astral liquid chromatography/mass spectrometry data‐independent acquisition (DIA) method. Raw files were analyzed using the DIA‐NN software, and results were further filtered by retaining only peptide spectrum matches with ≥99% confidence. Credible spectral peptides and proteins were retained. Differentially expressed proteins were defined as those with an absolute fold change >2 and *p*‐value < 0.05.

### 4.21 Statistical Analysis

Unless otherwise specified, statistical significance was determined using a two‐tailed Student's t‐test for comparisons between two groups or one‐way analysis of variance (ANOVA) for comparisons across multiple groups. Survival analyses were conducted using the log‐rank test, with Kaplan‐Meier curves used for visualization. Sample sizes were not pre‐determined statistically, but were chosen based on experimental feasibility and common practices. The exact sample size (n), measure of center (mean or median), measure of dispersion (SEM or SD), and number of independent replicates are provided in the respective figure legends. Replicates are defined as (i) separate tumors in xenograft assays and (ii) independent biological repeats for in vitro assays. For all notched box plots presented in this study: the lower and upper bounds of the box represent the 25_th_ and 75_th_ percentiles, respectively; the central line denotes the median; the notch displays the 95% confidence interval around the median; and the whiskers extend to 1.5 times the interquartile range from the box.

## Author Contributions

Conceptualization: L.X., Y.C., and X.H. Methodology: L.X., Y.C., X.H., X.G., J.Z., Y.Z., and X.H(uang). Investigation: L.X., Y.C., X.H., J.W., X.G., J.Z., Y.Z., N.L., L.X(ie), X.H(uang), Z.Z., Z.R., X.Y., H.G., Y.W., L.M., J.Z(hang)., S.Z., J.L., W.C., and H.P.K. Writing – original draft: L.X., Y.C., X.H, and J.Z. Writing – review and editing: L.X., Y.C., X.H, J.Z., L.X(ie), Y.Z., X.H(uang), V.K.L., W.C., J.W., and H.P.K. Visualization: L.X., Y.C., X.H., J.Z., Y.Z., and X.H(uang). Funding acquisition: L.X., Y.C., X.H., J.W., and W.C. Resources: L.X., Y.C., X.H., W.C., V.K.L., J.W., and H.P.K.

## Funding

This work was funded by the Fundamental Research Funds for the Central Universities (226‐2025‐00101), the National Natural Science Foundation of China (32270746, 82203247 to L.X., 82203415 to Y.C., 82272637 to X.H.), and the Natural Science Foundation of Zhejiang Province (LZ24H160004 to Y.C. and LZ23C060002 to L.X.). It is additionally supported by the special fund for Innovative Development of Hangzhou Chengxi Science and Technology Innovation Corridor (to L.X.), and the Medical Interdisciplinary Innovation Program 2024, Zhejiang University School of Medicine (to W.C. and L.X.).

## Conflicts of Interest

The Zhejiang University has filed a patent application on these CDK9 degraders (application number: 202510478984.2). X.H., L.X., Y.C., X.G., and J.Z. are co‐inventors on this patent application. The authors declare no other conflict of interest.

## Supporting information




**Supporting File 1**: advs75854‐sup‐0001‐SuppMat.docx.

**Supporting File 2**: advs75854‐sup‐0002‐TableS1‐S5andTableS7‐S8.xlsx.

**Supporting File 3**: advs75854‐sup‐0003‐SuppMat.docx.

## Data Availability

Raw ChIP‐seq, CUT&Tag‐seq and RNA‐seq data generated from in this study have been deposited in the Genome Sequence Archive in National Genomics Data Center, China National Center for Bioinformation (https://ngdc.cncb.ac.cn/gsa), and are available under accession HRA013377. Mass spectrometry data were deposited in OMIX (https://ngdc.cncb.ac.cn/omix; accession no. OMIX016548). Publicly available p53 ChIP‐seq data from SJSA1 cells (GSE86164) can be downloaded from GEO database (https://www.ncbi.nlm.nih.gov/geo/). Cancer dependency data were retrieved from DepMap. Copy number data and the associated survival information related to OS and EWS were retrieved from cBio Portal (https://www.cbioportal.org/) and PedcBioPortal (https://pedcbioportal.kidsfirstdrc.org/) by 2025‐Aug.

## References

[1] T. G. Grünewald , M. Alonso , S. Avnet , et al., “Sarcoma Treatment in the Era of Molecular Medicine,” EMBO Molecular Medicine 12 (2020): 11131, 10.15252/emmm.201911131. PMC7645378 33047515

[2] D. Chen , Z. Zhao , Z. Huang , et al., “Super Enhancer Inhibitors Suppress MYC Driven Transcriptional Amplification and Tumor Progression in Osteosarcoma,” Bone Research 6 (2018): 11, 10.1038/s41413-018-0009-8. 29644114 PMC5884797

[3] D. Y. Lu , J. M. Ellegast , K. N. Ross , et al., “The ETS Transcription Factor ETV6 Constrains the Transcriptional Activity of EWS‐FLI to Promote Ewing Sarcoma,” Nature Cell Biology 25 (2023): 285–297, 10.1038/s41556-022-01059-8. 36658220 PMC9928584

[4] Y. Y. Kim , B. E. Gryder , R. Sinniah , et al., “KDM3B Inhibitors Disrupt the Oncogenic Activity of PAX3‐FOXO1 in Fusion‐Positive Rhabdomyosarcoma,” Nature Communications 15 (2024): 1703, 10.1038/s41467-024-45902-y. PMC10894237 38402212

[5] X. Shi , Y. Zheng , L. Jiang , et al., “EWS‐FLI1 Regulates and Cooperates With Core Regulatory Circuitry in Ewing Sarcoma,” Nucleic Acids Research 48 (2020): 11434–11451, 10.1093/nar/gkaa901. 33080033 PMC7672457

[6] N. Corradini , N. Andre , and D. Orbach , “Maintenance Therapy for Pediatric Sarcoma: Full Throttle Ahead?,” Current Opinion in Oncology 37 (2025): 347–357, 10.1097/CCO.0000000000001148. 40421977

[7] Y. Wang , C. Yu , G. Pei , W. Jia , T. Li , and P. Li , “Dissolution of Oncofusion Transcription Factor Condensates for Cancer Therapy,” Nature Chemical Biology 19 (2023): 1223–1234, 10.1038/s41589-023-01376-5. 37400539

[8] Y. Chen , L. Xu , R. Y. Lin , M. Muschen , and H. P. Koeffler , “Core Transcriptional Regulatory Circuitries in Cancer,” Oncogene 39 (2020): 6633–6646, 10.1038/s41388-020-01459-w. 32943730 PMC7581508

[9] S. J. Vervoort , J. R. Devlin , N. Kwiatkowski , M. Teng , N. S. Gray , and R. W. Johnstone , “Targeting Transcription Cycles in Cancer,” Nature Reviews Cancer 22 (2022): 5–24, 10.1038/s41568-021-00411-8. 34675395

[10] K. Fujinaga , F. Huang , and B. M. Peterlin , “P‐TEFb: The Master Regulator of Transcription Elongation,” Molecular Cell 83 (2023): 393–403, 10.1016/j.molcel.2022.12.006. 36599353 PMC9898187

[11] B. N. Devaiah , B. A. Lewis , N. Cherman , et al., “BRD4 is an Atypical Kinase That Phosphorylates Serine2 of the RNA Polymerase II Carboxy‐Terminal Domain,” Proceedings of the National Academy of Sciences of the United States of America 109 (2012): 6927–6932, 10.1073/pnas.1120422109. 22509028 PMC3345009

[12] N. W. Harper , G. A. Birdsall , M. E. Honeywell , K. M. Ward , A. A. Pai , and M. J. Lee , “RNA Pol II Inhibition Activates Cell Death Independently From the Loss of Transcription,” Cell 188 (2025): 6301–6316, 10.1016/j.cell.2025.07.034. 40818455 PMC12406974

[13] D. Wu , H. Yin , C. Yang , et al., “The BET PROTAC Inhibitor GNE‐987 Displays Anti‐Tumor Effects by Targeting Super‐Enhancers Regulated Gene in Osteosarcoma,” BMC cancer 24 (2024): 928, 10.1186/s12885-024-12691-y. 39090568 PMC11292958

[14] S. Zheng , Y. Chen , Z. Zhu , et al., “Exploiting Targeted Degradation of Cyclins and Cyclin‐Dependent Kinases for Cancer Therapeutics: A Review,” Journal of Zhejiang University‐SCIENCE B 26 (2025): 713–739, 10.1631/jzus.B2500021. 40866321 PMC12390393

[15] M. Bekes , D. R. Langley , and C. M. Crews , “PROTAC Targeted Protein Degraders: The Past Is Prologue,” Nature Reviews Drug Discovery 21 (2022): 181–200, 10.1038/s41573-021-00371-6. 35042991 PMC8765495

[16] L. Ma , L. Xie , Y. Wang , et al., “Discovery of dCDK9‐202 as a Highly Potent and Selective PROTAC CDK9 Degrader With Strong in Vivo Antitumor Activity,” Journal of Medicinal Chemistry 68 (2025): 21172–21186, 10.1021/acs.jmedchem.5c01111. 41066447 PMC12557382

[17] L. Xu , Y. Chen , A. Mayakonda , et al., “Targetable BET Proteins‐ and E2F1‐Dependent Transcriptional Program Maintains the Malignancy of Glioblastoma,” Proceedings of the National Academy of Sciences of the United States of America 115 (2018): E5086–E5095, 10.1073/pnas.1712363115. 29764999 PMC5984485

[18] C.‐H. Luo , L.‐H. Hu , J.‐Y. Liu , et al., “CDK9 Recruits HUWE1 to Degrade RARα and Offers Therapeutic Opportunities for Cutaneous T‐Cell Lymphoma,” Nature Communications 15 (2024): 10594, 10.1038/s41467-024-54354-3. PMC11618697 39632829

[19] C. M. Olson , B. Jiang , M. A. Erb , et al., “Pharmacological Perturbation of CDK9 Using Selective CDK9 Inhibition or Degradation,” Nature Chemical Biology 14 (2018): 163–170, 10.1038/nchembio.2538. 29251720 PMC5912898

[20] I. Tortorelli , A. Napolitano , Y. Zhou , P. Huang , and R. L. Jones , “MDM2 Inhibitors in Sarcomas: Results and Next Steps,” Current Opinion in Oncology 37 (2025): 324–330, 10.1097/CCO.0000000000001146. 40464483

[21] B. Stolte , A. B. Iniguez , N. V. Dharia , et al., “Genome‐Scale CRISPR‐Cas9 Screen Identifies Druggable Dependencies in TP53 Wild‐Type Ewing Sarcoma,” Journal of Experimental Medicine 215 (2018): 2137–2155, 10.1084/jem.20171066. 30045945 PMC6080915

[22] J. Hines , S. Lartigue , H. Dong , Y. Qian , and C. M. Crews , “MDM2‐Recruiting PROTAC Offers Superior, Synergistic Antiproliferative Activity via Simultaneous Degradation of BRD4 and Stabilization of p53,” Cancer Research 79 (2019): 251–262, 10.1158/0008-5472.CAN-18-2918. 30385614 PMC6318015

[23] X. Han , W. Wei , and Y. Sun , “PROTAC Degraders With Ligands Recruiting MDM2 E3 Ubiquitin Ligase: An Updated Perspective,” Acta Materia Medica 1 (2022): 244–259, 10.15212/AMM-2022-0010. 35734447 PMC9211018

[24] S. Su , Z. Yang , H. Gao , et al., “Potent and Preferential Degradation of CDK6 via Proteolysis Targeting Chimera Degraders,” Journal of Medicinal Chemistry 62 (2019): 7575–7582, 10.1021/acs.jmedchem.9b00871. 31330105 PMC6790125

[25] Z. Geng , E. Wafula , R. J. Corbett , et al., “The Open Pediatric Cancer Project,” Gigascience 14 (2025): giaf093, 10.1093/gigascience/giaf093. 40891528 PMC12402770

[26] H. de Traux de Wardin , J. K. Dermawan , M. S. Merlin , et al., “Sequential Genomic Analysis Using a Multisample/Multiplatform Approach to Better Define Rhabdomyosarcoma Progression and Relapse,” NPJ Precision Oncology 7 (2023): 96, 10.1038/s41698-023-00445-1. 37730754 PMC10511463

[27] A. Zehir , R. Benayed , R. H. Shah , et al., “Mutational Landscape of Metastatic Cancer Revealed From Prospective Clinical Sequencing of 10,000 Patients,” Nature Medicine 23 (2017): 703–713, 10.1038/nm.4333. PMC5461196 28481359

[28] S. M. Bevill , S. Casaní‐Galdón , C. A. El Farran , et al., “Impact of Supraphysiologic MDM2 Expression on Chromatin Networks and Therapeutic Responses in Sarcoma,” Cell Genomics 3 (2023): 100321, 10.1016/j.xgen.2023.100321. 37492096 PMC10363746

[29] V. Ellison , A. Polotskaia , G. Xiao , et al., “A Cancer Persistent DNA Repair Circuit Driven by MDM2, MDM4 (MDMX), and Mutant p53 for Recruitment of MDC1 and 53BP1 on Chromatin,” Nucleic Acids Research 53 (2025): gkaf627, 10.1093/nar/gkaf627. 40626562 PMC12235508

[30] Y. Chen , L. Xu , A. Mayakonda , et al., “Bromodomain and Extraterminal Proteins Foster the Core Transcriptional Regulatory Programs and Confer Vulnerability in Liposarcoma,” Nature Communications 10 (2019): 1353, 10.1038/s41467-019-09257-z. PMC6430783 30903020

[31] Y. Sui , T. Wang , Y. Mei , et al., “Targeting Super‐Enhancer–Driven Transcriptional Dependencies Suppresses Aberrant Hedgehog Pathway Activation and Overcomes Smoothened Inhibitor Resistance,” Cancer Research 84 (2024): 2690–2706, 10.1158/0008-5472.CAN-23-3306. 38775809

[32] B. Zheng , S. Gold , M. Iwanaszko , B. C. Howard , L. Wang , and A. Shilatifard , “Distinct Layers of BRD4‐PTEFb Reveal Bromodomain‐Independent Function in Transcriptional Regulation,” Molecular Cell 83 (2023): 2896–2910.e4, 10.1016/j.molcel.2023.06.032. 37442129 PMC10527981

[33] J. D. Weissman , A. K. Singh , B. N. Devaiah , P. Schuck , R. C. LaRue , and D. S. Singer , “The Intrinsic Kinase Activity of BRD4 Spans Its BD2‐B‐BID Domains,” Journal of Biological Chemistry 297 (2021): 101326, 10.1016/j.jbc.2021.101326. 34688663 PMC8591364

[34] R. P. Nowak , S. L. DeAngelo , D. Buckley , et al., “Plasticity in Binding Confers Selectivity in Ligand‐Induced Protein Degradation,” Nature Chemical Biology 14 (2018): 706–714, 10.1038/s41589-018-0055-y. 29892083 PMC6202246

[35] L. Planas‐Paz , A. Pliego‐Mendieta , C. Hagedorn , et al., “Unravelling Homologous Recombination Repair Deficiency and Therapeutic Opportunities in Soft Tissue and Bone Sarcoma,” EMBO Molecular Medicine 15 (2023): 16863, 10.15252/emmm.202216863. PMC10086583 36779660

[36] M. Wienken , A. Dickmanns , A. Nemajerova , et al., “MDM2 Associates With Polycomb Repressor Complex 2 and Enhances Stemness‐Promoting Chromatin Modifications Independent of p53,” Molecular Cell 61 (2016): 68–83, 10.1016/j.molcel.2015.12.008. 26748827 PMC6284523

[37] C. M. Adams , R. Mitra , Y. Xiao , et al., “Targeted MDM2 Degradation Reveals a New Vulnerability for p53‐Inactivated Triple‐Negative Breast Cancer,” Cancer Discovery 13 (2023): 1210–1229, 10.1158/2159-8290.CD-22-1131. 36734633 PMC10164114

[38] T. H. Cheng and S. N. Cohen , “Human MDM2 Isoforms Translated Differentially on Constitutive versus p53‐Regulated Transcripts Have Distinct Functions in the p53/MDM2 and TSG101/MDM2 Feedback Control Loops,” Molecular and Cellular Biology 27 (2007): 111–119, 10.1128/MCB.00235-06. 17060450 PMC1800643

[39] M. Saadatzadeh , A. Elmi , P. Pandya , et al., “The Role of MDM2 in Promoting Genome Stability versus Instability,” International Journal of Molecular Sciences 18 (2017): 2216, 10.3390/ijms18102216. 29065514 PMC5666895

[40] Y. Wang , M. Wang , L. Ma , et al., “Identification of a Potent and Selective CDK9 Degrader as a Targeted Therapeutic Option for the Treatment of Small‐Cell Lung Cancer,” Journal of Medicinal Chemistry 68 (2025): 2528–2550, 10.1021/acs.jmedchem.4c01621. 39895086

[41] Y. Zeng , J. Xiao , Y. Xu , et al., “Degradation of Cyclin‐Dependent Kinase 9/Cyclin T1 by Optimized Microtubule‐Associated Protein 1 Light Chain 3 Beta‐Recruiting Coumarin Analogs,” Journal of Medicinal Chemistry 66 (2023): 12877–12893, 10.1021/acs.jmedchem.3c00828. 37671907

[42] J. Li , T. Liu , Y. Song , et al., “Discovery of Small‐Molecule Degraders of the CDK9‐Cyclin T1 Complex for Targeting Transcriptional Addiction in Prostate Cancer,” Journal of Medicinal Chemistry 65 (2022): 11034–11057, 10.1021/acs.jmedchem.2c00257. 35925880

[43] R. Lin , J. Yang , T. Liu , et al., “Discovery of HyT‐Based Degraders of CDK9‐Cyclin T1 Complex,” Chemistry & Biodiversity 20 (2023): 202300769, 10.1002/cbdv.202300769. 37349855

[44] Y. Zhong , J. Xu , H. Cao , et al., “First ATG101‐Recruiting Small Molecule Degrader for Selective CDK9 Degradation via Autophagy–Lysosome Pathway,” Acta Pharmaceutica Sinica B 15 (2025): 2612–2624, 10.1016/j.apsb.2025.03.047. 40487652 PMC12145019

[45] L. Alfano , C. A. Iannuzzi , D. Barone , et al., “CDK9‐55 Guides the Anaphase‐Promoting Complex/Cyclosome (APC/C) in Choosing the DNA Repair Pathway Choice,” Oncogene 43 (2024): 1263–1273, 10.1038/s41388-024-02982-w. 38433256

[46] X. Peng , X. Huang , S. Zhang , et al., “Sequential Inhibition of PARP and BET as a Rational Therapeutic Strategy for Glioblastoma,” Advanced Science 11 (2024): 2307747, 10.1002/advs.202307747. 38896791 PMC11321613

[47] X. Fan , Y. Zhang , Z. Yang , et al., “XPO1 Inhibitor KPT‐330 Disrupts the Core Transcriptional Regulatory Circuitry of Dedifferentiated Liposarcoma by Modulating the Translation Process,” Oncogene 45 (2026): 2029–2045, 10.1038/s41388-026-03794-w. 41991999

[48] S. Zheng , W. Wang , J. Aldahdooh , et al., “SynergyFinder plus: Toward Better Interpretation and Annotation of Drug Combination Screening Datasets,” Genomics, Proteomics & Bioinformatics 20 (2022): 587–596, 10.1016/j.gpb.2022.01.004. PMC9801064 35085776

[49] Q. Ding , Z. Zhang , J.‐J. Liu , et al., “Discovery of RG7388, a Potent and Selective p53–MDM2 Inhibitor in Clinical Development,” Journal of Medicinal Chemistry 56 (2013): 5979–5983, 10.1021/jm400487c. 23808545

[50] D. B. Freeman , T. D. Hopkins , P. J. Mikochik , et al., “Discovery of KB‐0742, a Potent, Selective, Orally Bioavailable Small Molecule Inhibitor of CDK9 for MYC‐Dependent Cancers,” Journal of Medicinal Chemistry 66 (2023): 15629–15647, 10.1021/acs.jmedchem.3c01233. 37967851 PMC10726352

[51] S. Jiang , H. Li , L. Zhang , et al., “Generic Diagramming Platform (GDP): A Comprehensive Database of High‐Quality Biomedical Graphics,” Nucleic Acids Research 53 (2025): D1670–D1676, 10.1093/nar/gkae973. 39470721 PMC11701665

