# Supplementary material for: Harnessing MDM2‐Mediated Targeted Degradation of Transcriptional and Epigenetic Machinery to Disrupt Oncogenic Addictions in Pediatric Sarcoma
Source: Adv Sci (Weinh). 2026 Jun 19;13(45):e23088. doi: 10.1002/advs.202523088 (PMC13336031; doi:10.1002/advs.202523088)
Supplement: Supplementary file 2 [file ADVS-13-e23088-s003.docx]

**Supplementary Note**

**Chemistry**

**General Experiment and Information.**

Unless otherwise noted, all purchased reagents were used as received without further purification. ^1^H NMR and ^13^C NMR spectra were recorded on a Bruker Advance 400 MHz spectrometer. ^1^H NMR spectra are reported in parts per million (ppm) downfield from tetramethylsilane (TMS). All ^13^C NMR spectral peaks are reported in ppm and measured with ^1^H decoupling. In reported spectral data, the format (δ) chemical shift (multiplicity, J values in Hz, integration) was used with the following abbreviations: s = singlet, d = doublet, t = triplet, q = quartet, m = multiplet. Mass spectrometric (MS) analysis was carried out with a Waters UPLC mass spectrometer. The final compounds were all purified by C18 reverse phase preparative HPLC column with solvent A (0.1% HCOOH in H_2_O) and solvent B (0.1% HCOOH in MeOH) as eluents. The purity of all the final compounds was shown to be >95% by UPLC−MS or UPLC.

**Synthesis of N-(5-(((5-(tert-butyl)oxazol-2-yl)methyl)thio)thiazol-2-yl)piperidine-4-carboxamide (SNS032).**

Reaction conditions: (a) TEA, DCM, 0 ^o^C; (b) POCl_3_, reflux; (c) MeOH, r.t.; (d) **26**, NaBH_4_, EtOH, Acetone, r.t.~reflux; (e) HATU, DIPEA, DMF, r.t.; TFA, DCM, r.t..

Triethylamine (3.5 eq.) was added to a solution of compound **1** (1.51 g, 10 mmol, 1.0 eq.) in 35 mL DCM at -5°C. After 30 min, compound **2** (1.1 eq.) was slowly added dropwise at -5°C. After 1 h at rt, the reaction mixture was quenched with 1 N HCl and extracted with DCM three times. The organic layer was separated, washed with brine, dried and evaporated. The final compound **3** was obtained by flash column chromatography (PE/EA = 20:1) with 95% yield.

3 mL POCl_3_ was slowly added to a 10 mL round bottomed flask with compound **3** (958 mg, 5 mmol, 1.0 eq.). The reaction mixture was heated to 105°C and stirred for 1 h. The resulting mixture was cooled, concentrated in vacuo, quenched with water and extracted with EA three times. The organic phase was neutralized to pH 7-8 with saturated sodium bicarbonate, separated, washed with brine, dried, and evaporated. The pure product **4** was obtained by flash column chromatography (PE/EA = 9:1) with 76% yield.

A mixture of compound **5** (1.30 g, 5 mmol, 1.0 eq.) and potassium thiocyanate (4.0 eq.) in 34 mL MeOH was stirred at rt for 48 h. MeOH was evaporated. The residue was added 3 mL water and adjusted the pH of the solution to pH = 12 with 10% NaOH. The resulting solid was filtered to obtain the product **7** with 32% yield.

NaBH_4_ (2.0 eq.) was slowly added to a solution of compound **7** (314 mg, 2 mmol, 1.0 eq.) in 20 mL absolute EtOH at rt. After 1 h, 10 mL acetone was slowly introduced. After 1 h at rt, compound **4** (1.0 eq.) in 3 mL EtOH was added, and the reaction mixture was heated to reflux for 1 h. The resulting mixture was cooled, concentrated in vacuo, quenched with water and extracted with EA three times. The organic phase was separated, dried, and concentrated in vacuo. The product **8** was obtained by flash column chromatography (PE/EA = 4:1) with 60% yield.

DIPEA (5.0 eq.) and HATU (1.5 eq.) were added to a solution of compound **8** (539 mg, 2 mmol, 1.0 eq.) and compound **9** (1.0 eq.) in DMF. The resulting mixture was stirred at rt overnight. The mixture was quenched with water and extracted with EA three times. The organic phase was separated, dried, and concentrated in vacuo. Then the residue was purified by flash column chromatography (PE/EA = 2:1) to afford the white solid, which was dissolved in 1:3 TFA/DCM. After 30 min, the solvents were evaporated under reduced pressure to give the corresponding deprotected compound **10** (SNS032) (TFA salt) that was used in the following reactions without further purification (59% yield).

***Synthesis of N*-(5-(((5-(*tert*-butyl)oxazol-2-yl)methyl)thio)thiazol-2-yl)-1-(6-(4-((2*R*,3*S*,4*R*,5*S*)-3-(3-chloro-2-fluorophenyl)-4-(4-chloro-2-fluorophenyl)-4-cyano-5-neopentylpyrrolidine-2-carboxamido)-3-methoxybenzamido)hexanoyl)piperidine-4-carboxamide (dCDK9-001).**

Reaction conditions: (a) HATU, DIPEA, DMF, r.t.; (b) TFA, DCM, r.t.; (c) HATU, DIPEA, DMF, r.t..

DIPEA (5 eq.) and HATU (1.2 eq.) were added to a solution of compound **SNS032** (381 mg, 1 mmol) and a series of linear acids, referred to as compound **11** (1.1 eq.) in DMF (2 mL). After 30 min at rt, the mixture was subject to prep-HPLC to afford the intermediate. A solution of the intermediate in 1:1 TFA/DCM was stirred at rt for 30 min. The solvents were evaporated under reduced pressure to give the corresponding deprotected intermediates **12** (TFA salt) that were used in the following reactions without further puriﬁcation (55% yield with two steps).

DIPEA (5 eq.) and HATU (1.2 eq.) were added to a solution of compound **12** (49.2 mg, 0.1 mmol) and compound **Idasanutlin** (1.1 eq.) in DMF (2 mL). After 30 min at rt, the mixture was subject to prep-HPLC to afford final compound **dCDK9-001**.

White solid; Yield: 65%. ^1^H NMR (600 MHz, DMSO-*d*_6_) *δ* 12.31 (s, 1H), 10.41 (s, 1H), 8.41 (t, *J* = 5.7 Hz, 1H), 8.32 (d, *J* = 8.4 Hz, 1H), 7.74 (t, *J* = 7.2 Hz, 1H), 7.62 – 7.46 (m, 4H), 7.43 – 7.30 (m, 4H), 6.71 (s, 1H), 4.59 (d, *J* = 7.4 Hz, 2H), 4.39 (dd, *J* = 12.7, 6.9 Hz, 2H), 4.05 (s, 2H), 3.93 (s, 5H), 3.26 (q, *J* = 6.7 Hz, 2H), 3.03 (t, *J* = 11.6 Hz, 1H), 2.77 – 2.69 (m, 1H), 2.58 (t, *J* = 11.2 Hz, 1H), 2.32 (q, *J* = 7.5 Hz, 2H), 1.85 – 1.75 (m, 2H), 1.65 (dd, *J* = 14.3, 9.9 Hz, 1H), 1.54 (p, *J* = 7.5 Hz, 5H), 1.35 – 1.30 (m, 2H), 1.28 – 1.23 (m, 2H), 1.17 (s, 9H), 0.98 (s, 9H). ^13^C NMR (150 MHz, DMSO-*d*_6_) *δ* 172.61, 170.41, 169.81, 164.67, 160.59, 160.22, 159.74, 158.13, 158.08, 155.78, 154.14, 146.89, 144.54, 134.19, 134.11, 130.38, 129.41, 129.30, 128.56, 128.01, 125.49, 125.41, 125.04, 124.71, 124.68, 119.52, 119.34, 118.93, 118.80, 118.62, 118.55, 118.08, 117.06, 116.88, 116.72, 116.38, 108.96, 64.04, 62.86, 62.81, 62.65, 62.61, 55.19, 49.56, 43.64, 43.28, 40.66, 33.38, 31.68, 30.31, 29.50, 28.90, 28.48, 27.80, 27.69, 27.18, 25.69, 24.02. HRMS (ESI) calculated for C_54_H_63_Cl_2_F_2_N_8_O_6_S_2_ [M + H]^+^: 1091.3652, found: 1091.3627. UPLC-retention time: 6.718 min, purity >95%.

***Synthesis of N*-(5-(((5-(*tert*-butyl)oxazol-2-yl)methyl)thio)thiazol-2-yl)-1-(7-(4-((2*R*,3*S*,4*R*,5*S*)-3-(3-chloro-2-fluorophenyl)-4-(4-chloro-2-fluorophenyl)-4-cyano-5-neopentylpyrrolidine-2-carboxamido)-3-methoxybenzamido)heptanoyl)piperidine-4-carboxamide (dCDK9-002).**

Reaction conditions: (a) HATU, DIPEA, DMF, r.t.; (b) TFA, DCM, r.t.; (c) HATU, DIPEA, DMF, r.t..

DIPEA (5 eq.) and HATU (1.2 eq.) were added to a solution of compound **SNS032** (381 mg, 1 mmol) and a series of linear acids, referred to as compound **13** (1.1 eq.) in DMF (2 mL). After 30 min at rt, the mixture was subject to prep-HPLC to afford the intermediate. A solution of the intermediate in 1:1 TFA/DCM was stirred at rt for 30 min. The solvents were evaporated under reduced pressure to give the corresponding deprotected intermediates **14** (TFA salt) that were used in the following reactions without further puriﬁcation (55% yield with two steps).

DIPEA (5 eq.) and HATU (1.2 eq.) were added to a solution of compound **14** (50.6 mg, 0.1 mmol) and compound **Idasanutlin** (1.1 eq.) in DMF (2 mL). After 30 min at rt, the mixture was subject to prep-HPLC to afford final compound **dCDK9-002**.

White solid; Yield: 60%. ^1^H NMR (600 MHz, DMSO-*d*_6_) *δ* 12.31 (s, 1H), 10.41 (s, 1H), 8.40 (t, *J* = 5.7 Hz, 1H), 8.32 (d, *J* = 8.4 Hz, 1H), 7.74 (t, *J* = 7.2 Hz, 1H), 7.62 – 7.46 (m, 4H), 7.44 – 7.30 (m, 4H), 6.71 (s, 1H), 4.60 (d, *J* = 7.4 Hz, 2H), 4.39 (d, *J* = 13.1 Hz, 2H), 4.05 (s, 2H), 3.93 (s, 5H), 3.25 (q, *J* = 6.5 Hz, 2H), 3.02 (t, *J* = 11.7 Hz, 1H), 2.77 – 2.69 (m, 1H), 2.63 – 2.54 (m, 1H), 2.31 (q, *J* = 7.2 Hz, 2H), 1.87 – 1.76 (m, 2H), 1.65 (dd, *J* = 14.2, 9.8 Hz, 1H), 1.52 (dq, *J* = 14.7, 7.1 Hz, 5H), 1.33 – 1.23 (m, 6H), 1.17 (s, 9H), 0.98 (s, 9H). ^13^C NMR (150 MHz, DMSO-*d*_6_) *δ* 172.61, 170.41, 169.85, 164.69, 160.60, 160.21, 159.74, 158.13, 158.08, 155.79, 154.15, 146.89, 144.54, 134.19, 134.12, 130.39, 130.36, 129.41, 129.31, 128.55, 128.01, 125.49, 125.41, 125.03, 124.71, 124.68, 119.52, 119.34, 118.93, 118.80, 118.61, 118.55, 118.09, 117.06, 116.88, 116.73, 116.39, 108.96, 64.04, 62.65, 62.61, 55.19, 49.55, 43.65, 43.28, 33.38, 31.68, 30.32, 29.50, 28.90, 28.52, 28.43, 27.97, 27.79, 27.68, 27.20, 25.80, 24.23. HRMS (ESI) calculated for C_55_H_65_Cl_2_F_2_N_8_O_6_S_2_ [M + H]^+^: 1105.3808, found: 1105.3797. UPLC-retention time: 7.047 min, purity >95%.

***Synthesis of N*-(5-(((5-(*tert*-butyl)oxazol-2-yl)methyl)thio)thiazol-2-yl)-1-(8-(4-((2*R*,3*S*,4*R*,5*S*)-3-(3-chloro-2-fluorophenyl)-4-(4-chloro-2-fluorophenyl)-4-cyano-5-neopentylpyrrolidine-2-carboxamido)-3-methoxybenzamido)octanoyl)piperidine-4-carboxamide (dCDK9-003).**

Reaction conditions: (a) HATU, DIPEA, DMF, r.t.; (b) TFA, DCM, r.t.; (c) HATU, DIPEA, DMF, r.t..

DIPEA (5 eq.) and HATU (1.2 eq.) were added to a solution of compound **SNS032** (381 mg, 1 mmol) and a series of linear acids, referred to as compound **15** (1.1 eq.) in DMF (2 mL). After 30 min at rt, the mixture was subject to prep-HPLC to afford the intermediate. A solution of the intermediate in 1:1 TFA/DCM was stirred at rt for 30 min. The solvents were evaporated under reduced pressure to give the corresponding deprotected intermediates **16** (TFA salt) that were used in the following reactions without further puriﬁcation (65% yield with two steps).

DIPEA (5 eq.) and HATU (1.2 eq.) were added to a solution of compound **16** (52 mg, 0.1 mmol) and compound **Idasanutlin** (1.1 eq.) in DMF (2 mL). After 30 min at rt, the mixture was subject to prep-HPLC to afford final compound **dCDK9-003**.

White solid; Yield: 55%. ^1^H NMR (600 MHz, DMSO-*d*_6_) *δ* 12.31 (s, 1H), 10.41 (s, 1H), 8.40 (t, *J* = 5.7 Hz, 1H), 8.32 (d, *J* = 8.2 Hz, 1H), 7.74 (t, *J* = 7.4 Hz, 1H), 7.62 – 7.45 (m, 4H), 7.44 – 7.32 (m, 4H), 6.71 (s, 1H), 4.60 (d, *J* = 7.1 Hz, 2H), 4.42 – 4.34 (m, 2H), 4.05 (s, 2H), 3.98 – 3.88 (m, 5H), 3.25 (q, *J* = 6.8 Hz, 2H), 3.02 (t, *J* = 12.0 Hz, 1H), 2.78 – 2.69 (m, 1H), 2.63 – 2.55 (m, 1H), 2.30 (q, *J* = 7.1 Hz, 2H), 1.80 (t, *J* = 13.2 Hz, 2H), 1.65 (dd, *J* = 14.2, 9.9 Hz, 1H), 1.51 (dt, *J* = 21.5, 7.2 Hz, 5H), 1.31 – 1.23 (m, 8H), 1.17 (s, 9H), 0.98 (s, 9H). ^13^C NMR (150 MHz, DMSO-*d*_6_) *δ* 172.61, 170.41, 169.86, 164.68, 160.61, 160.21, 159.74, 158.13, 158.08, 155.78, 154.15, 146.89, 144.54, 134.19, 134.12, 130.38, 129.41, 129.31, 128.55, 128.01, 125.49, 125.41, 125.03, 124.71, 124.68, 119.52, 119.33, 118.92, 118.80, 118.62, 118.55, 118.08, 117.06, 116.88, 116.73, 116.39, 108.95, 64.04, 62.87, 62.82, 62.65, 62.61, 55.19, 49.56, 43.65, 43.28, 33.38, 31.69, 30.32, 29.50, 28.90, 28.58, 28.18, 28.16, 28.07, 27.80, 27.68, 27.20, 25.95, 25.86, 24.23. HRMS (ESI) calculated for C_56_H_67_Cl_2_F_2_N_8_O_6_S_2_ [M + H]^+^: 1119.3965, found: 1119.3949. UPLC-retention time: 6.451 min, purity >95%.

***Synthesis of N*-(5-(((5-(*tert*-butyl)oxazol-2-yl)methyl)thio)thiazol-2-yl)-1-(9-(4-((2*R*,3*S*,4*R*,5*S*)-3-(3-chloro-2-fluorophenyl)-4-(4-chloro-2-fluorophenyl)-4-cyano-5-neopentylpyrrolidine-2-carboxamido)-3-methoxybenzamido)nonanoyl)piperidine-4-carboxamide (dCDK9-004).**

Reaction conditions: (a) HATU, DIPEA, DMF, r.t.; (b) TFA, DCM, r.t.; (c) HATU, DIPEA, DMF, r.t..

DIPEA (5 eq.) and HATU (1.2 eq.) were added to a solution of compound **SNS032** (381 mg, 1 mmol) and a series of linear acids, referred to as compound **17** (1.1 eq.) in DMF (2 mL). After 30 min at rt, the mixture was subject to prep-HPLC to afford the intermediate. A solution of the intermediate in 1:1 TFA/DCM was stirred at rt for 30 min. The solvents were evaporated under reduced pressure to give the corresponding deprotected intermediates **18** (TFA salt) that were used in the following reactions without further puriﬁcation (60% yield with two steps).

DIPEA (5 eq.) and HATU (1.2 eq.) were added to a solution of compound **18** (53.4 mg, 0.1 mmol) and compound **Idasanutlin** (1.1 eq.) in DMF (2 mL). After 30 min at rt, the mixture was subject to prep-HPLC to afford final compound **dCDK9-004**.

White solid; Yield: 60%. ^1^H NMR (500 MHz, DMSO-*d*_6_) *δ* 12.30 (s, 1H), 10.41 (s, 1H), 8.39 (t, *J* = 5.7 Hz, 1H), 8.32 (d, *J* = 8.4 Hz, 1H), 7.74 (ddd, *J* = 8.2, 6.5, 1.6 Hz, 1H), 7.60 – 7.47 (m, 4H), 7.43 – 7.33 (m, 4H), 6.71 (s, 1H), 4.60 (d, *J* = 7.2 Hz, 2H), 4.39 (d, *J* = 12.8 Hz, 2H), 4.05 (s, 2H), 3.96 (t, *J* = 11.2 Hz, 2H), 3.93 (s, 3H), 3.25 (q, *J* = 6.6 Hz, 2H), 3.02 (t, *J* = 11.5 Hz, 1H), 2.72 (ddt, *J* = 11.4, 7.6, 4.0 Hz, 1H), 2.63 – 2.54 (m, 1H), 2.29 (q, *J* = 7.5 Hz, 2H), 1.85 – 1.76 (m, 2H), 1.65 (dd, *J* = 14.1, 9.8 Hz, 1H), 1.51 (dt, *J* = 20.9, 7.1 Hz, 5H), 1.28 (d, *J* = 14.6 Hz, 10H), 1.17 (s, 9H), 0.98 (s, 9H). ^13^C NMR (125 MHz, DMSO-*d*_6_) *δ* 173.67, 171.47, 170.93, 165.75, 161.67, 161.28, 159.20, 158.98, 157.03, 155.07, 147.97, 145.60, 135.27, 135.18, 131.45, 130.48, 130.39, 129.63, 129.09, 126.57, 126.47, 126.10, 125.77, 120.59, 120.40, 120.02, 119.87, 119.69, 119.62, 119.17, 118.15, 117.93, 117.79, 117.47, 110.03, 65.12, 63.95, 63.73, 63.68, 56.26, 50.63, 44.73, 44.36, 41.73, 40.77, 34.46, 32.78, 31.38, 30.56, 29.97, 29.66, 29.34, 29.26, 29.22, 28.87, 28.75, 28.27, 26.98, 25.36. HRMS (ESI) calculated for C_57_H_69_Cl_2_F_2_N_8_O_6_S_2_ [M + H]^+^: 1133.4121, found: 1133.4111. UPLC-retention time: 6.953 min, purity >95%.

***Synthesis of N*-(5-(((5-(*tert*-butyl)oxazol-2-yl)methyl)thio)thiazol-2-yl)-1-(10-(4-((2*R*,3*S*,4*R*,5*S*)-3-(3-chloro-2-fluorophenyl)-4-(4-chloro-2-fluorophenyl)-4-cyano-5-neopentylpyrrolidine-2-carboxamido)-3-methoxybenzamido)decanoyl)piperidine-4-carboxamide (dCDK9-005).**

Reaction conditions: (a) HATU, DIPEA, DMF, r.t.; (b) TFA, DCM, r.t.; (c) HATU, DIPEA, DMF, r.t..

DIPEA (5 eq.) and HATU (1.2 eq.) were added to a solution of compound **SNS032** (381 mg, 1 mmol) and a series of linear acids, referred to as compound **19** (1.1 eq.) in DMF (2 mL). After 30 min at rt, the mixture was subject to prep-HPLC to afford the intermediate. A solution of the intermediate in 1:1 TFA/DCM was stirred at rt for 30 min. The solvents were evaporated under reduced pressure to give the corresponding deprotected intermediates **20** (TFA salt) that were used in the following reactions without further puriﬁcation (70% yield with two steps).

DIPEA (5 eq.) and HATU (1.2 eq.) were added to a solution of compound **20** (54.8 mg, 0.1 mmol) and compound **Idasanutlin** (1.1 eq.) in DMF (2 mL). After 30 min at rt, the mixture was subject to prep-HPLC to afford final compound **dCDK9-005**.

White solid; Yield: 65%. ^1^H NMR (600 MHz, DMSO-*d*_6_) *δ* 12.31 (s, 1H), 10.41 (s, 1H), 8.40 (t, *J* = 5.7 Hz, 1H), 8.32 (d, *J* = 8.2 Hz, 1H), 7.74 (t, *J* = 7.2 Hz, 1H), 7.60 – 7.47 (m, 4H), 7.43 – 7.33 (m, 4H), 6.71 (s, 1H), 4.59 (d, *J* = 7.1 Hz, 2H), 4.39 (dd, *J* = 13.2, 4.5 Hz, 2H), 4.05 (s, 2H), 3.98 – 3.89 (m, 5H), 3.25 (q, *J* = 6.7 Hz, 2H), 3.02 (t, *J* = 11.6 Hz, 1H), 2.77 – 2.69 (m, 1H), 2.58 (t, *J* = 11.3 Hz, 1H), 2.29 (q, *J* = 7.6 Hz, 2H), 1.87 – 1.76 (m, 2H), 1.65 (dd, *J* = 14.3, 9.7 Hz, 1H), 1.55 – 1.45 (m, 5H), 1.32 – 1.25 (m, 12H), 1.17 (s, 9H), 0.98 (s, 9H). ^13^C NMR (150 MHz, DMSO-*d*_6_) *δ* 172.61, 170.41, 169.86, 164.67, 160.61, 160.21, 159.74, 158.13, 158.08, 155.78, 154.15, 146.88, 144.54, 134.19, 134.11, 130.38, 130.35, 129.41, 129.31, 129.04, 128.55, 128.02, 125.49, 125.41, 125.04, 124.71, 124.68, 119.52, 119.33, 118.92, 118.80, 118.62, 118.55, 118.07, 117.06, 116.88, 116.72, 116.38, 108.95, 64.04, 62.85, 62.65, 62.61, 55.19, 49.56, 43.65, 43.28, 33.38, 31.70, 30.32, 29.50, 28.90, 28.59, 28.49, 28.43, 28.34, 28.28, 28.20, 28.18, 28.15, 27.98, 27.80, 27.68, 27.20, 25.92, 24.29. HRMS (ESI) calculated for C_58_H_71_Cl_2_F_2_N_8_O_6_S_2_ [M + H]^+^: 1147.4278, found: 1147.4246. UPLC-retention time: 6.928 min, purity >95%.

***Synthesis of N*-(5-(((5-(*tert*-butyl)oxazol-2-yl)methyl)thio)thiazol-2-yl)-1-(11-(4-((2*R*,3*S*,4*R*,5*S*)-3-(3-chloro-2-fluorophenyl)-4-(4-chloro-2-fluorophenyl)-4-cyano-5-neopentylpyrrolidine-2-carboxamido)-3-methoxybenzamido)undecanoyl)piperidine-4-carboxamide (dCDK9-006).**

Reaction conditions: (a) HATU, DIPEA, DMF, r.t.; (b) TFA, DCM, r.t.; (c) HATU, DIPEA, DMF, r.t..

DIPEA (5 eq.) and HATU (1.2 eq.) were added to a solution of compound **SNS032** (381 mg, 1 mmol) and a series of linear acids, referred to as compound **21** (1.1 eq.) in DMF (2 mL). After 30 min at rt, the mixture was subject to prep-HPLC to afford the intermediate. A solution of the intermediate in 1:1 TFA/DCM was stirred at rt for 30 min. The solvents were evaporated under reduced pressure to give the corresponding deprotected intermediates **22** (TFA salt) that were used in the following reactions without further puriﬁcation (65% yield with two steps).

DIPEA (5 eq.) and HATU (1.2 eq.) were added to a solution of compound **22** (56.2 mg, 0.1 mmol) and compound **Idasanutlin** (1.1 eq.) in DMF (2 mL). After 30 min at rt, the mixture was subject to prep-HPLC to afford final compound **dCDK9-006**.

White solid; Yield: 60%. ^1^H NMR (600 MHz, DMSO-*d*_6_) *δ* 12.31 (s, 1H), 10.41 (s, 1H), 8.39 (t, *J* = 5.9 Hz, 1H), 8.32 (d, *J* = 8.4 Hz, 1H), 7.74 (t, *J* = 7.4 Hz, 1H), 7.61 – 7.52 (m, 3H), 7.48 (d, *J* = 8.4 Hz, 1H), 7.42 – 7.33 (m, 4H), 6.71 (s, 1H), 4.60 (d, *J* = 7.6 Hz, 2H), 4.43 – 4.35 (m, 2H), 4.05 (s, 2H), 3.96 (t, *J* = 11.0 Hz, 1H), 3.92 (s, 3H), 3.90 (d, *J* = 13.6 Hz, 1H), 3.25 (q, *J* = 6.4 Hz, 2H), 3.02 (t, *J* = 12.8 Hz, 1H), 2.77 – 2.69 (m, 1H), 2.58 (t, *J* = 12.5 Hz, 1H), 2.29 (q, *J* = 7.6 Hz, 2H), 1.81 (t, *J* = 14.0 Hz, 2H), 1.65 (dd, *J* = 14.4, 9.8 Hz, 1H), 1.55 – 1.49 (m, 3H), 1.47 (t, *J* = 7.1 Hz, 2H), 1.30 – 1.23 (m, 14H), 1.17 (d, *J* = 2.1 Hz, 9H), 0.98 (d, *J* = 1.9 Hz, 9H). ^13^C NMR (150 MHz, DMSO-*d*_6_) *δ* 170.42, 169.86, 164.67, 160.63, 160.22, 159.75, 158.14, 158.09, 155.80, 154.16, 146.89, 144.55, 134.20, 134.13, 130.39, 130.37, 129.42, 129.32, 129.05, 128.56, 128.02, 125.51, 125.42, 125.05, 124.72, 124.68, 119.53, 119.34, 118.94, 118.81, 118.62, 118.56, 118.08, 117.07, 116.89, 116.73, 116.39, 108.96, 64.06, 62.87, 62.66, 62.62, 55.19, 49.57, 43.66, 43.29, 40.67, 39.70, 33.39, 31.71, 30.70, 30.32, 29.50, 28.91, 28.60, 28.50, 28.45, 28.39, 28.37, 28.32, 28.25, 28.22, 28.16, 28.11, 28.00, 27.81, 27.69, 27.21, 25.94, 24.31. HRMS (ESI) calculated for C_59_H_73_Cl_2_F_2_N_8_O_6_S_2_ [M + H]^+^: 1161.4434, found: 1161.4417. UPLC-retention time: 7.370 min, purity >95%.

***Synthesis of N*-(5-(((5-(*tert*-butyl)oxazol-2-yl)methyl)thio)thiazol-2-yl)-1-(12-(4-((2*R*,3*S*,4*R*,5*S*)-3-(3-chloro-2-fluorophenyl)-4-(4-chloro-2-fluorophenyl)-4-cyano-5-neopentylpyrrolidine-2-carboxamido)-3-methoxybenzamido)dodecanoyl)piperidine-4-carboxamide (dCDK9-007).**

Reaction conditions: (a) HATU, DIPEA, DMF, r.t.; (b) TFA, DCM, r.t.; (c) HATU, DIPEA, DMF, r.t..

DIPEA (5 eq.) and HATU (1.2 eq.) were added to a solution of compound **SNS032** (381 mg, 1 mmol) and a series of linear acids, referred to as compound **23** (1.1 eq.) in DMF (2 mL). After 30 min at rt, the mixture was subject to prep-HPLC to afford the intermediate. A solution of the intermediate in 1:1 TFA/DCM was stirred at rt for 30 min. The solvents were evaporated under reduced pressure to give the corresponding deprotected intermediates **24** (TFA salt) that were used in the following reactions without further puriﬁcation (70% yield with two steps).

DIPEA (5 eq.) and HATU (1.2 eq.) were added to a solution of compound **24** (57.6 mg, 0.1 mmol) and compound **Idasanutlin** (1.1 eq.) in DMF (2 mL). After 30 min at rt, the mixture was subject to prep-HPLC to afford final compound **dCDK9-007**.

White solid; Yield: 55%. ^1^H NMR (600 MHz, DMSO-*d*_6_) *δ* 12.31 (s, 1H), 10.41 (s, 1H), 8.39 (t, *J* = 5.7 Hz, 1H), 8.32 (d, *J* = 8.4 Hz, 1H), 7.74 (t, *J* = 7.2 Hz, 1H), 7.61 – 7.45 (m, 4H), 7.42 – 7.32 (m, 4H), 6.71 (s, 1H), 4.60 (d, *J* = 7.4 Hz, 2H), 4.39 (dd, *J* = 12.8, 7.6 Hz, 2H), 4.05 (s, 2H), 3.98 – 3.88 (m, 5H), 3.25 (q, *J* = 6.7 Hz, 2H), 3.02 (t, *J* = 11.7 Hz, 1H), 2.77 – 2.69 (m, 1H), 2.58 (t, *J* = 11.3 Hz, 1H), 2.34 – 2.23 (m, 2H), 1.86 – 1.76 (m, 2H), 1.65 (dd, *J* = 14.3, 9.7 Hz, 1H), 1.50 (dt, *J* = 27.5, 7.2 Hz, 5H), 1.27 (d, *J* = 20.7 Hz, 16H), 1.17 (s, 9H), 0.98 (s, 9H). ^13^C NMR (150 MHz, DMSO-*d*_6_) δ 172.62, 170.42, 169.87, 164.67, 160.65, 160.22, 159.75, 158.14, 155.80, 154.16, 146.89, 144.54, 134.20, 134.13, 130.40, 129.42, 129.32, 128.56, 125.51, 125.42, 125.05, 124.72, 119.53, 119.34, 118.94, 118.81, 118.63, 118.56, 118.07, 117.07, 116.89, 116.73, 116.39, 108.95, 64.06, 62.89, 62.67, 55.19, 49.56, 43.67, 43.29, 40.67, 33.39, 31.71, 30.33, 29.50, 28.91, 28.59, 28.41, 28.22, 27.82, 27.69, 27.21, 25.93, 24.30. HRMS (ESI) calculated for C_60_H_75_Cl_2_F_2_N_8_O_6_S_2_ [M + H]^+^: 1175.4591, found: 1175.4566. UPLC-retention time: 7.551 min, purity >95%.

***Synthesis of N*-(5-(((5-(*tert*-butyl)oxazol-2-yl)methyl)thio)thiazol-2-yl)-1-(3-(2-(4-((2*R*,3*S*,4*R*,5*S*)-3-(3-chloro-2-fluorophenyl)-4-(4-chloro-2-fluorophenyl)-4-cyano-5-neopentylpyrrolidine-2-carboxamido)-3-methoxybenzamido)ethoxy)propanoyl)piperidine-4-carboxamide (dCDK9-008).**

Reaction conditions: (a) HATU, DIPEA, DMF, r.t.; (b) TFA, DCM, r.t.; (c) HATU, DIPEA, DMF, r.t..

DIPEA (5 eq.) and HATU (1.2 eq.) were added to a solution of compound **SNS032** (381 mg, 1 mmol) and a series of linear acids, referred to as compound **25** (1.1 eq.) in DMF (2 mL). After 30 min at rt, the mixture was subject to prep-HPLC to afford the intermediate. A solution of the intermediate in 1:1 TFA/DCM was stirred at rt for 30 min. The solvents were evaporated under reduced pressure to give the corresponding deprotected intermediates **26** (TFA salt) that were used in the following reactions without further puriﬁcation (65% yield with two steps).

DIPEA (5 eq.) and HATU (1.2 eq.) were added to a solution of compound **26** (49.4 mg, 0.1 mmol) and compound **Idasanutlin** (1.1 eq.) in DMF (2 mL). After 30 min at rt, the mixture was subject to prep-HPLC to afford final compound **dCDK9-008**.

White solid; Yield: 60%. ^1^H NMR (600 MHz, DMSO-*d*_6_) *δ* 12.31 (s, 1H), 10.42 (s, 1H), 8.46 (t, *J* = 6.1 Hz, 1H), 8.33 (d, *J* = 8.4 Hz, 1H), 7.74 (t, *J* = 7.5 Hz, 1H), 7.60 – 7.47 (m, 4H), 7.44 – 7.32 (m, 4H), 6.71 (s, 1H), 4.60 (s, 2H), 4.38 (d, *J* = 6.8 Hz, 2H), 4.05 (s, 2H), 3.99 – 3.93 (m, 2H), 3.93 (s, 3H), 3.67 (t, *J* = 6.7 Hz, 2H), 3.53 (t, *J* = 6.1 Hz, 2H), 3.43 (d, *J* = 6.5 Hz, 2H), 3.08 – 2.98 (m, 1H), 2.72 (t, *J* = 11.3 Hz, 1H), 2.61 (dd, *J* = 10.9, 5.2 Hz, 2H), 1.80 (t, *J* = 12.5 Hz, 2H), 1.66 (d, *J* = 9.8 Hz, 1H), 1.57 (t, *J* = 12.4 Hz, 1H), 1.41 (t, *J* = 12.5 Hz, 1H), 1.31 – 1.22 (m, 2H), 1.16 (s, 9H), 0.97 (s, 9H). ^13^C NMR (150 MHz, DMSO-*d*_6_) *δ* 172.60, 170.44, 168.09, 164.92, 160.63, 160.22, 159.74, 158.14, 158.08, 155.79, 154.15, 146.89, 144.54, 134.19, 134.12, 130.39, 130.36, 129.42, 129.00, 128.70, 128.01, 125.49, 125.40, 125.03, 124.71, 119.51, 119.45, 118.93, 118.81, 118.61, 118.55, 118.07, 117.06, 116.88, 116.73, 116.40, 109.02, 68.11, 65.81, 65.78, 64.04, 62.86, 62.65, 62.61, 55.20, 49.56, 43.68, 43.28, 40.59, 39.71, 39.44, 33.39, 32.12, 30.31, 29.49, 28.90, 27.67. HRMS (ESI) calculated for C_53_H_61_Cl_2_F_2_N_8_O_7_S_2_ [M + H]^+^: 1093.3444, found: 1093.3407. UPLC-retention time: 6.415 min, purity >95%.

***Synthesis of N*-(5-(((5-(*tert*-butyl)oxazol-2-yl)methyl)thio)thiazol-2-yl)-1-(3-(2-(2-(4-((2*R*,3*S*,4*R*,5*S*)-3-(3-chloro-2-fluorophenyl)-4-(4-chloro-2-fluorophenyl)-4-cyano-5-neopentylpyrrolidine-2-carboxamido)-3-methoxybenzamido)ethoxy)ethoxy)propanoyl)piperidine-4-carboxamide (dCDK9-009).**

Reaction conditions: (a) HATU, DIPEA, DMF, r.t.; (b) TFA, DCM, r.t.; (c) HATU, DIPEA, DMF, r.t..

DIPEA (5 eq.) and HATU (1.2 eq.) were added to a solution of compound **SNS032** (381 mg, 1 mmol) and a series of linear acids, referred to as compound **27** (1.1 eq.) in DMF (2 mL). After 30 min at rt, the mixture was subject to prep-HPLC to afford the intermediate. A solution of the intermediate in 1:1 TFA/DCM was stirred at rt for 30 min. The solvents were evaporated under reduced pressure to give the corresponding deprotected intermediates **28** (TFA salt) that were used in the following reactions without further puriﬁcation (70% yield with two steps).

DIPEA (5 eq.) and HATU (1.2 eq.) were added to a solution of compound **28** (54 mg, 0.1 mmol) and compound **Idasanutlin** (1.1 eq.) in DMF (2 mL). After 30 min at rt, the mixture was subject to prep-HPLC to afford final compound **dCDK9-009**.

White solid; Yield: 50%. ^1^H NMR (600 MHz, DMSO-*d*_6_) *δ* 12.31 (s, 1H), 10.42 (s, 1H), 8.50 (t, *J* = 5.7 Hz, 1H), 8.33 (d, *J* = 8.4 Hz, 1H), 7.74 (t, *J* = 7.2 Hz, 1H), 7.62 – 7.48 (m, 4H), 7.44 – 7.29 (m, 4H), 6.71 (s, 1H), 4.60 (d, *J* = 6.8 Hz, 2H), 4.38 (d, *J* = 12.5 Hz, 2H), 4.05 (s, 2H), 3.99 – 3.88 (m, 5H), 3.63 (t, *J* = 6.7 Hz, 2H), 3.56 – 3.50 (m, 6H), 3.42 (d, *J* = 5.7 Hz, 2H), 3.01 (t, *J* = 12.0 Hz, 1H), 2.75 – 2.69 (m, 1H), 2.61 – 2.53 (m, 2H), 1.80 (t, *J* = 11.4 Hz, 2H), 1.65 (dd, *J* = 14.4, 9.8 Hz, 1H), 1.62 – 1.52 (m, 1H), 1.45 – 1.36 (m, 1H), 1.29 – 1.23 (m, 2H), 1.17 (s, 9H), 0.98 (s, 9H). ^13^C NMR (150 MHz, DMSO-*d*_6_) *δ* 172.62, 170.43, 168.00, 164.95, 160.65, 159.75, 158.14, 158.08, 155.80, 154.16, 146.89, 144.54, 134.12, 130.36, 129.42, 129.00, 128.68, 128.01, 125.49, 125.05, 124.72, 124.68, 119.52, 119.48, 118.94, 118.81, 118.61, 118.55, 118.06, 117.06, 116.89, 116.73, 116.41, 109.00, 69.04, 68.41, 66.26, 64.04, 62.86, 62.65, 62.62, 55.20, 49.54, 43.72, 43.29, 40.61, 33.39, 32.16, 30.32, 29.50, 28.91, 28.11, 27.72, 27.56, 27.12. HRMS (ESI) calculated for C_55_H_65_Cl_2_F_2_N_8_O_8_S_2_ [M + H]^+^: 1137.3706, found: 1137.3675. UPLC-retention time: 6.189 min, purity >95%.

***Synthesis of N*-(5-(((5-(*tert*-butyl)oxazol-2-yl)methyl)thio)thiazol-2-yl)-1-(1-(4-((2*R*,3*S*,4*R*,5*S*)-3-(3-chloro-2-fluorophenyl)-4-(4-chloro-2-fluorophenyl)-4-cyano-5-neopentylpyrrolidine-2-carboxamido)-3-methoxyphenyl)-1-oxo-5,8,11-trioxa-2-azatetradecan-14-oyl)piperidine-4-carboxamide (dCDK9-010).**

Reaction conditions: (a) HATU, DIPEA, DMF, r.t.; (b) TFA, DCM, r.t.; (c) HATU, DIPEA, DMF, r.t..

DIPEA (5 eq.) and HATU (1.2 eq.) were added to a solution of compound **SNS032** (381 mg, 1 mmol) and a series of linear acids, referred to as compound **29** (1.1 eq.) in DMF (2 mL). After 30 min at rt, the mixture was subject to prep-HPLC to afford the intermediate. A solution of the intermediate in 1:1 TFA/DCM was stirred at rt for 30 min. The solvents were evaporated under reduced pressure to give the corresponding deprotected intermediates **30** (TFA salt) that were used in the following reactions without further puriﬁcation (78% yield with two steps).

DIPEA (5 eq.) and HATU (1.2 eq.) were added to a solution of compound **30** (58.2 mg, 0.1 mmol) and compound **Idasanutlin** (1.1 eq.) in DMF (2 mL). After 30 min at rt, the mixture was subject to prep-HPLC to afford final compound **dCDK9-010**.

White solid; Yield: 55%. ^1^H NMR (600 MHz, DMSO-*d*_6_) *δ* 12.29 (s, 1H), 10.41 (s, 1H), 8.49 (s, 1H), 8.32 (s, 1H), 7.73 (s, 1H), 7.60 – 7.47 (m, 4H), 7.37 (d, *J* = 23.1 Hz, 4H), 6.71 (s, 1H), 4.59 (s, 2H), 4.38 (s, 2H), 4.04 (s, 2H), 3.95 (s, 2H), 3.92 (s, 3H), 3.60 (s, 2H), 3.51 (d, *J* = 16.9 Hz, 10H), 3.41 (s, 2H), 3.01 (t, *J* = 14.8 Hz, 1H), 2.71 (s, 1H), 2.58 (s, 2H), 1.79 (s, 2H), 1.65 (d, *J* = 16.0 Hz, 1H), 1.55 (d, *J* = 12.5 Hz, 1H), 1.39 (d, *J* = 12.7 Hz, 1H), 1.29 – 1.21 (m, 2H), 1.17 (s, 9H), 0.98 (s, 9H). ^13^C NMR (150 MHz, DMSO-*d*_6_) *δ* 173.66, 171.50, 169.06, 166.01, 161.68, 161.29, 160.81, 159.20, 159.15, 156.86, 155.22, 147.95, 145.61, 135.26, 135.18, 131.42, 130.48, 130.06, 129.76, 129.08, 126.56, 126.46, 126.10, 125.78, 120.58, 120.53, 120.00, 119.87, 119.68, 119.62, 119.15, 118.13, 117.95, 117.79, 117.47, 110.06, 70.24, 70.20, 70.14, 69.46, 67.32, 65.11, 63.86, 63.73, 63.68, 56.27, 50.62, 44.79, 44.35, 41.67, 40.75, 34.45, 33.23, 31.39, 30.56, 29.97, 28.79, 28.75, 28.17. HRMS (ESI) calculated for C_57_H_69_Cl_2_F_2_N_8_O_9_S_2_ [M + H]^+^: 1181.3969, found: 1181.3917. UPLC-retention time: 6.147 min, purity >95%.
