# Supplementary material for: Harnessing MDM2‐Mediated Targeted Degradation of Transcriptional and Epigenetic Machinery to Disrupt Oncogenic Addictions in Pediatric Sarcoma
Source: Adv Sci (Weinh). 2026 Jun 19:e23088. Online ahead of print. doi: 10.1002/advs.202523088 (PMC13336031; doi:10.1002/advs.202523088)
Supplement: Supplementary file 1 — Supporting File 1: advs75854‐sup‐0001‐SuppMat.docx. [file ADVS-9999-e23088-s002.docx]

Supporting Information

**Harnessing MDM2-mediated targeted degradation of transcriptional and epigenetic machinery to disrupt oncogenic addictions in pediatric sarcoma**

*Jiawei Zhou*^1,2,3^, *Xian Guan*^4^, *Nan Li*^1,3^, *Ying Zhang*^2^, *Long Xie*^2^, *Xingze Huang*^2^, *Zhipeng Zhu*^2^, *Zhuolin Ren*^1,2^, *Xiaoyan Yu*^1,2^, *Hanjun Guo*^1,2,3^, *Yuanfang Wu*^1,2,3^, *Lin Ma*^4^, *Suya Zheng*^1,3^, *Jingyao Zhang*^3,5^, *Jiyang Liu*^2^, *Victor Kuanmin Lee*^6,7^, *Wenhao Chen*^3,5^, *H. Phillip Koeffler*^8^, *Jinhu Wang*^1,3,9,^*, *Xin Han*^4,9,^*, *Ye Chen*^1,3,9,^*, *Liang Xu*^2,9,10,^*

* Correspondence: Jinhu Wang (wjh@zju.edu.cn), Xin Han (xinhan@zju.edu.cn), Ye Chen (chenyephd@zju.edu.cn), Liang Xu (xuliang.phd@zju.edu.cn)

**Supplementary Figures**

**
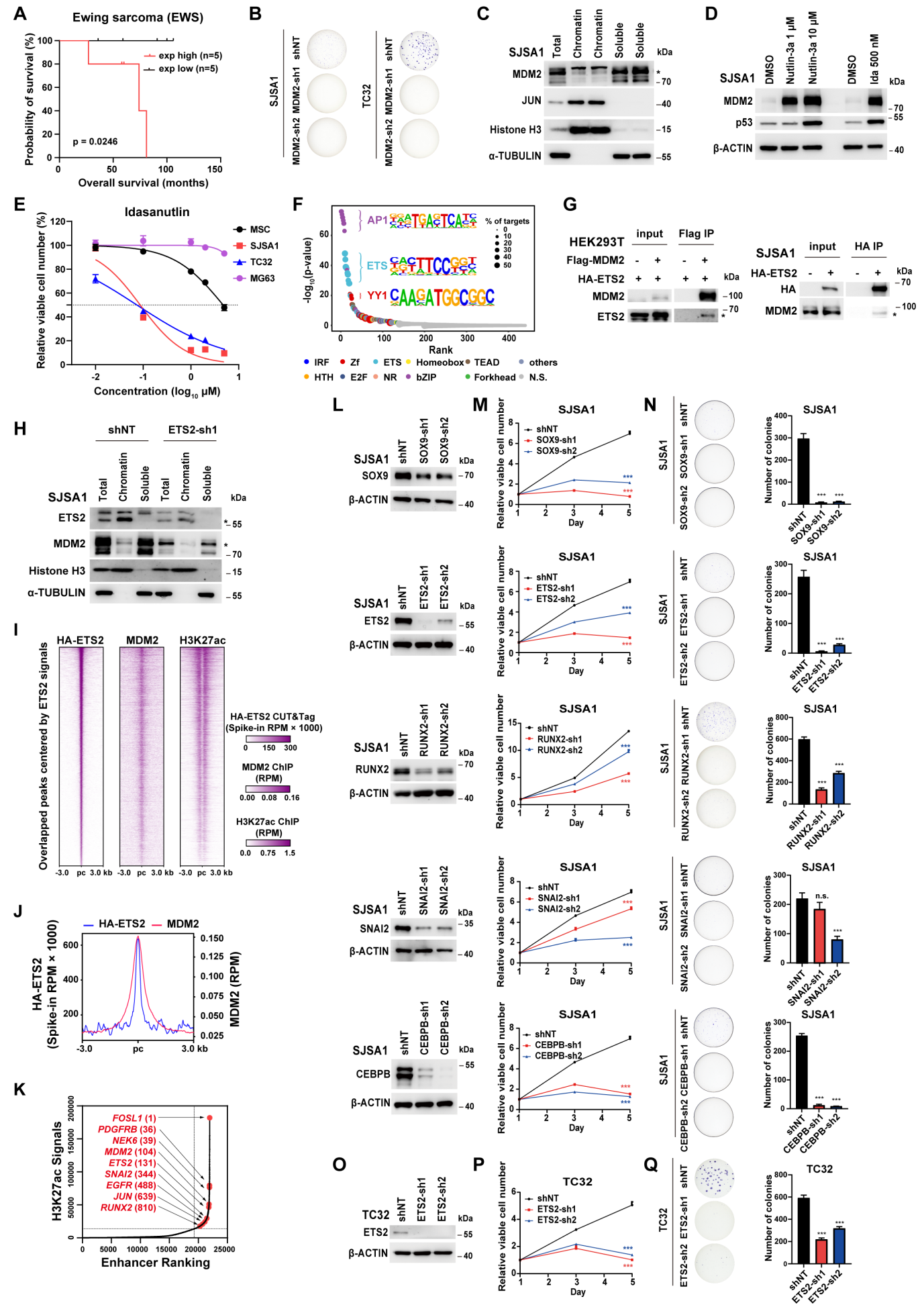
**

**Figure S1. MDM2 and MDM2^+^ promoter targets** **promote** **pediatric sarcomas.**

**(A)** Survival stratification of EWS (OpenPedCan Project v15 cohort, n=10) patients based on *MDM2* mRNA levels in sarcoma samples. Log-rank test was applied.

**(B)** Effect of MDM2 knockdown on the soft-agar colony-formation capability of SJSA1 cells.

**(C)** Chromatin loading of MDM2 in SJSA1 cells.

**(D)** Effect of MDM2 inhibitor treatment (24 h) on p53 expression in SJSA1 cells. Ida, Idasanutlin.

**(E)** Effect of MDM2 inhibitor treatment on the viability of sarcoma cells. Data are presented as mean±SEM; n=3.

**(F)** Known TF motifs enriched in the MDM2 binding sites.

**(G)** Co-immunoprecipitation assay between MDM2 (Flag-tagged or endogenous) and HA-ETS2.

**(H)** Effect of ETS2 knockdown on intracellular levels of MDM2 in SJSA1 cells.

**(I,J)** Heatmap and line plot showing the 1323 overlapped genomic binding peaks between MDM2 and HA-ETS2 in SJSA1 cells.

**(K)** Enhancer landscape in SJSA1 cells. Super-enhancers (SEs) and typical enhancers were identified using the ROSE algorithm, with representative SE targets and their enhancer signal rankings highlighted in red.

**(L-N)** Effects of shRNA-mediated silencing of MDM2^+^ promoter targets in SJSA1 cells on (**L**) the intracellular target proteins, (**M**) cell viability, and (**N**) cell colony-formation capability in soft agar. Data in (**M,N**) are presented as mean±SEM; n=3. One-way ANOVA was applied; ***, p<0.001. Mean relative viability and clonogenicity at the experimental endpoint are visualized in the heapmaps in Figure 1R and Figure 1S, respectively.

**(O-Q)** Effects of ETS2 knockdown in TC32 cells on (**O**) the intracellular target proteins, (**P**) cell viability, and (**Q**) cell colony-formation capability in soft agar. Data in (**P,Q**) are presented as mean±SEM; n=3. One-way ANOVA was applied; ***, p<0.001.

**
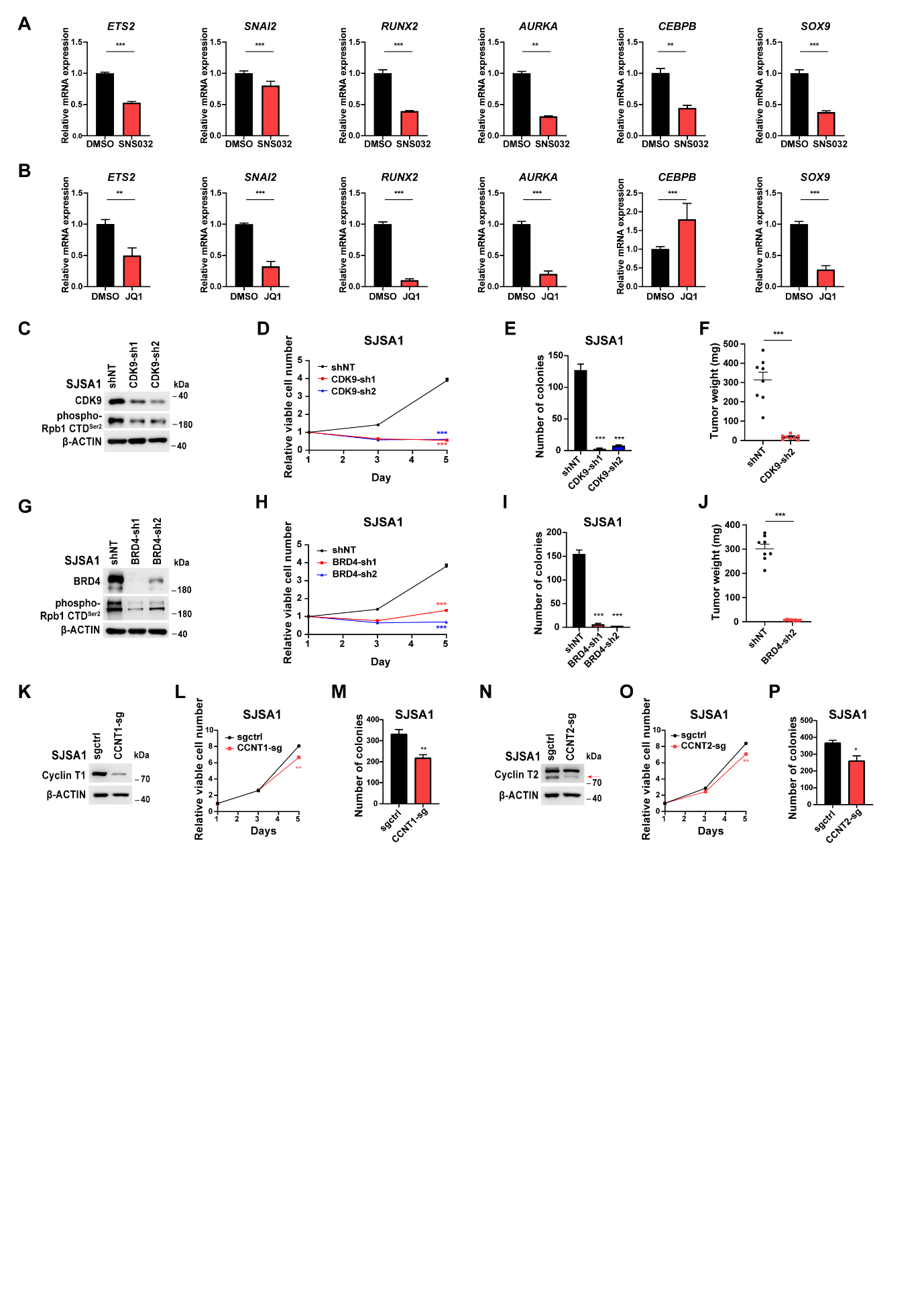
**

**Figure S2. P-TEFb and BRD4 maintain p53-independent MDM2 cistrome.**

**(A,B)** Transcriptional responses of MDM2 target genes to (**A**) CDK9 inhibitor (SNS032, 500 nM, 8 h) and (**B**) BET inhibitor (JQ1, 500 nM, 8 h) in SJSA1 cells.

**(C-F)** Effects of CDK9 knockdown in SJSA1 cells on (**C**) the intracellular target proteins, (**D**) cell viability, (**E**) soft-agar colony-formation capability, and (**F**) subcutaneous tumorigenicity in immunocompromised mice.

**(G-J)** Effects of BRD4 knockdown in SJSA1 cells on (**G**) the intracellular target proteins, (**H**) cell viability, (**I**) soft-agar colony-formation capability, and (**J**) subcutaneous tumorigenicity in immunocompromised mice.

**(K-M)** Effects of CRISPR/Cas9-mediated *CCNT1* editing in SJSA1 cells on (**K**) the intracellular target proteins, (**L**) cell viability, and (**M**) soft-agar colony-formation capability.

**(N-P)** Effects of CRISPR/Cas9-mediated *CCNT2* editing in SJSA1 cells on (**N**) the intracellular target proteins, (**O**) cell viability, and (**P**) soft-agar colony-formation capability. The red arrow indicates the responsive Cyclin T2 isoform in (**N**).

Data in (**A,B,D,E,H,I,L,M,O,P**) are presented as mean±SEM; n=3. Data in (**F,J**) are presented as mean±SEM; n=8. Statistics: (**A,B,F,J,L,M,O,P**) Student’s t-test (two-tailed), (**D,E,H,I**) one-way ANOVA. *, p<0.05; **, p<0.01; ***, p<0.001.

**
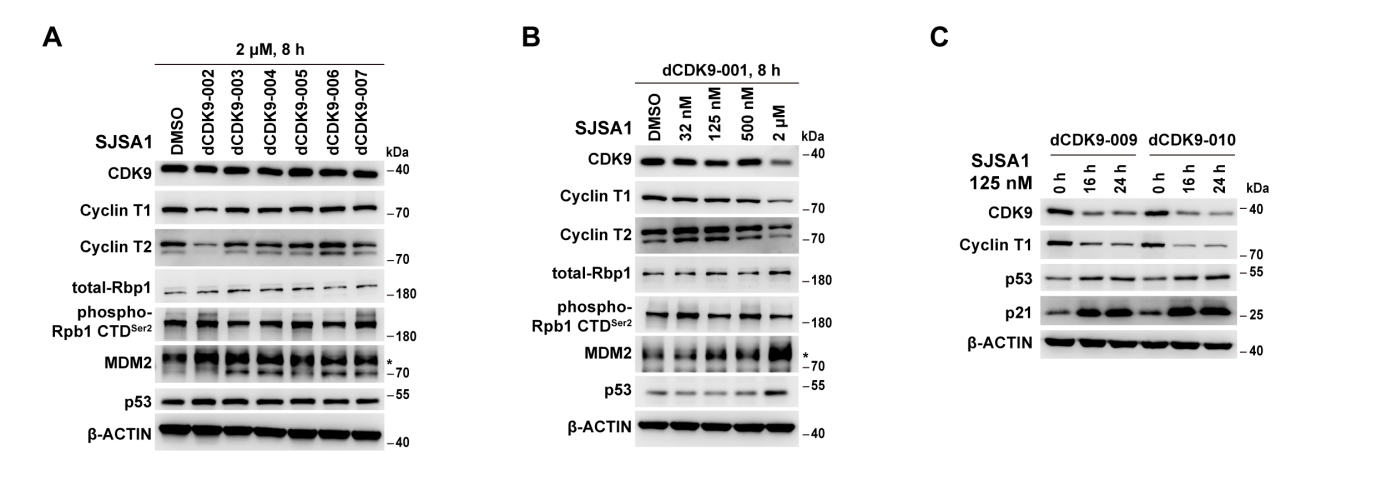
**

**Figure S3. Evaluation of the efficacy of novel MDM2-recruiting P-TEFb degraders.**

**(A)** Effects of dCDK9-002~007 on target protein expression in SJSA1 cells. * indicates the primary band at the expected molecular weight.

**(B)** Effects of dCDK9-001 on target protein expression in SJSA1 cells. * indicates the primary band at the expected molecular weight.

**(C)** Temporal effects of dCDK9-009 and dCDK9-010 on indicated proteins in SJSA cells.

**
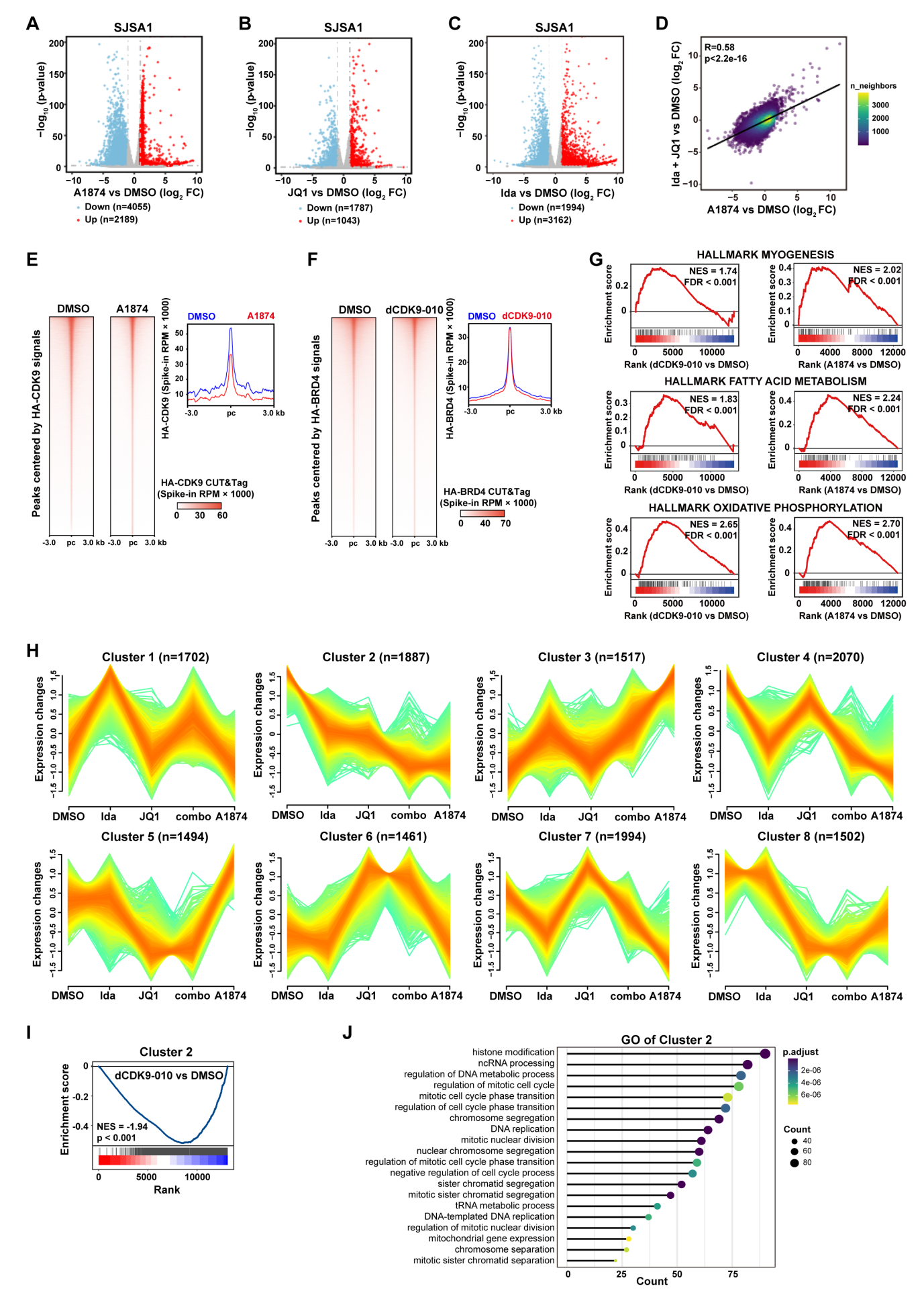
**

**Figure S4. Transcriptomic response to MDM2-TEMADs in pediatric sarcoma cells.**

**(A-C)** Volcano plots showing the differentially expressed genes in SJSA1 cells receiving indicated treatments (500 nM, 8 h). FC, fold change.

**(D)** Correlation analysis of transcriptomic profiles from SJSA1 cells treated with either A1874 or JQ1/Idasanutlin combo. Spearman test was applied.

**(E)** Effect of A1874-mediated BET degradation (500 nM, 8 h) on genome-wide chromatin binding signals of HA-CDK9 (CUT&Tag-seq) in SJSA1 cells.

**(F)** Effect of dCDK9-010-mediated P-TEFb degradation (500 nM, 8 h) on genome-wide chromatin binding signals of HA-BRD4 (CUT&Tag-seq) in SJSA1 cells.

**(G)** Common positively enriched gene sets from GSEA in sarcoma cells treated with dCDK9-010 and A1874.

**(H)** Gene expression clustering across treatment conditions using Mfuzz, revealing eight distinct co-expression patterns.

**(I)** Enrichment analyses of the gene set of Cluster 2 in SJSA1 cells receiving dCDK9-010 versus DMSO treatment.

**(J)** Gene ontology (GO) analysis of Cluster 2 genes.

**
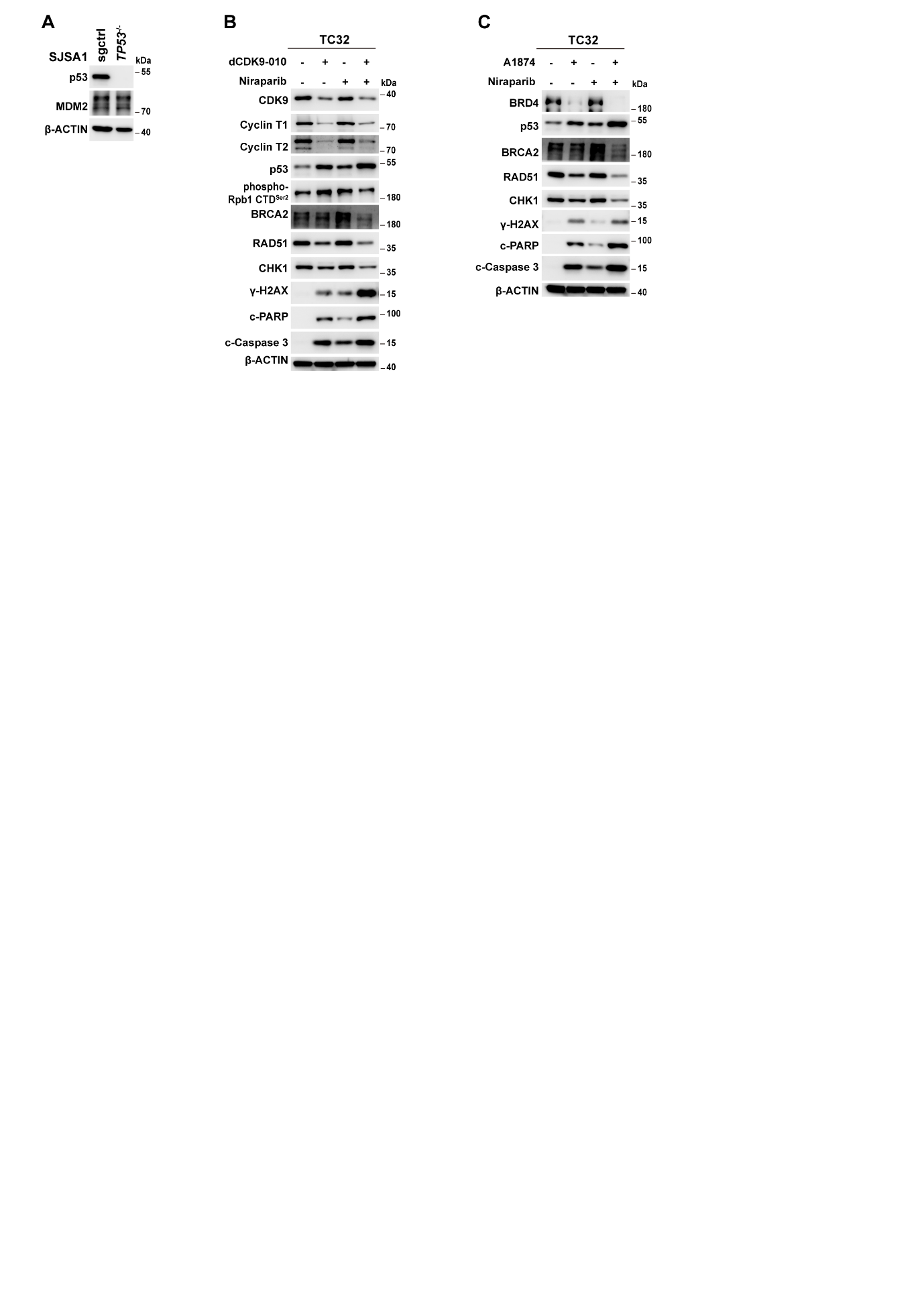
**

**Figure S5. MDM2-TEMADs sensitize sarcoma cells to Niraparib.**

**(A)** Effect of CRISPR/Cas9-mediated *TP53* knockout in SJSA1 cells.

**(B,C)** Effects of MDM2-TEMADs (dCDK9-010, 500 nM, 24 h; A1874, 250 nM, 24 h), Niraparib (500 nM, 24 h), and their combination on downstream protein expression in TC32 cells.

**
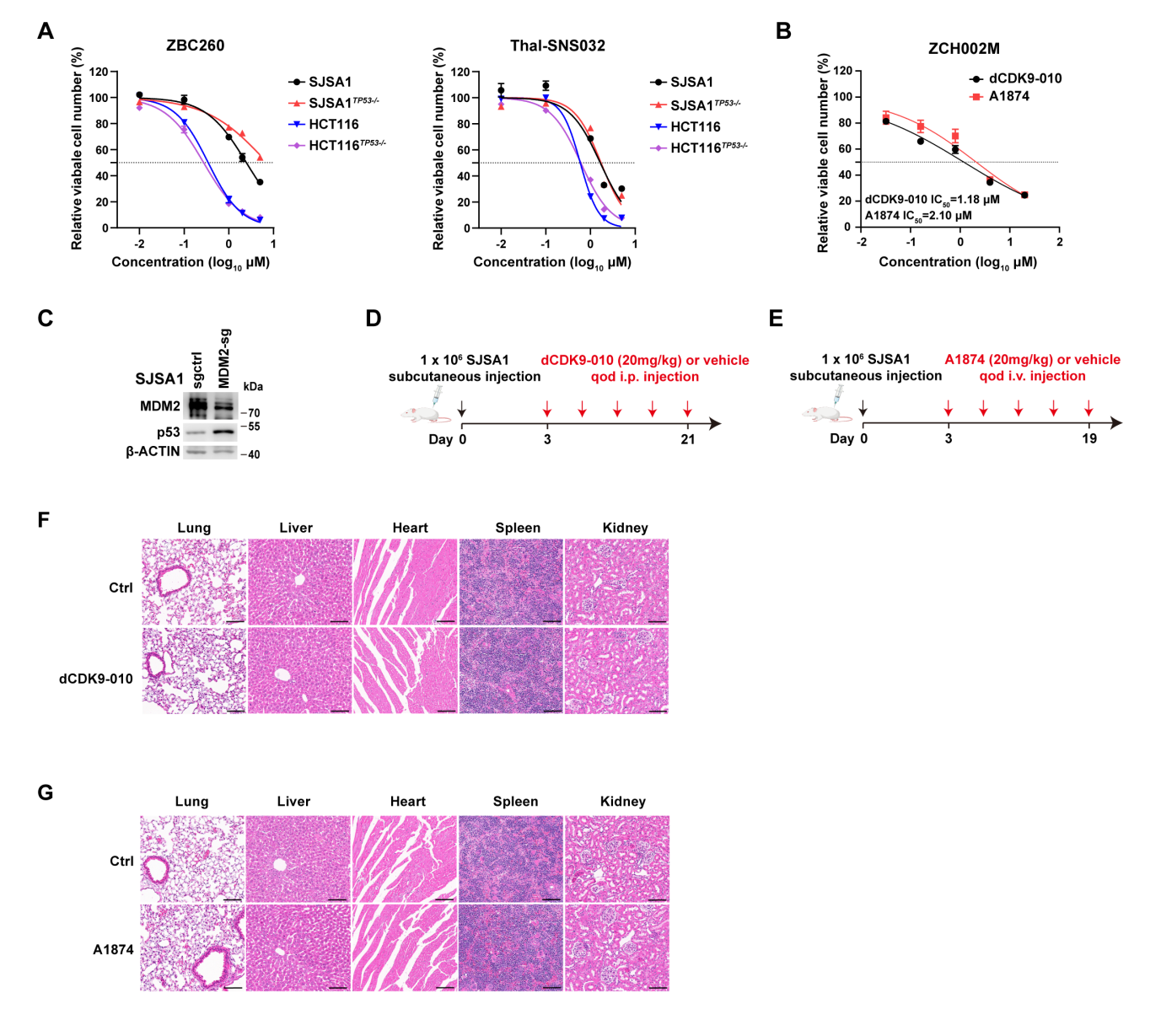
**

**Figure S6. MDM2-TEMADs exhibit encouraging anti-sarcoma efficacy.**

**(A)** Cellular responsiveness to CRBN-recruiting BET or CDK9 PROTACs. Data are presented as mean±SEM; n=3.

**(B)** Dose-response curves showing the effects of dCDK9-010 and A1874 on viability of an MDM2-overexpressing primary OS explant line ZCH002M. Data are presented as mean±SEM; n=3.

**(C)** Effect of CRISPR/Cas9-mediated *MDM2* editing in SJSA1 cells.

**(D,E)** Schematic of the MDM2-TEMAD treatment regimen in SJSA1 xenograft models.

**(F,G)** Histological examination of vital organs at the endpoint of *in vivo* efficacy experiments for (**F**) dCDK9-010 and (**G**) A1874. Scale bar, 100 μm.

**
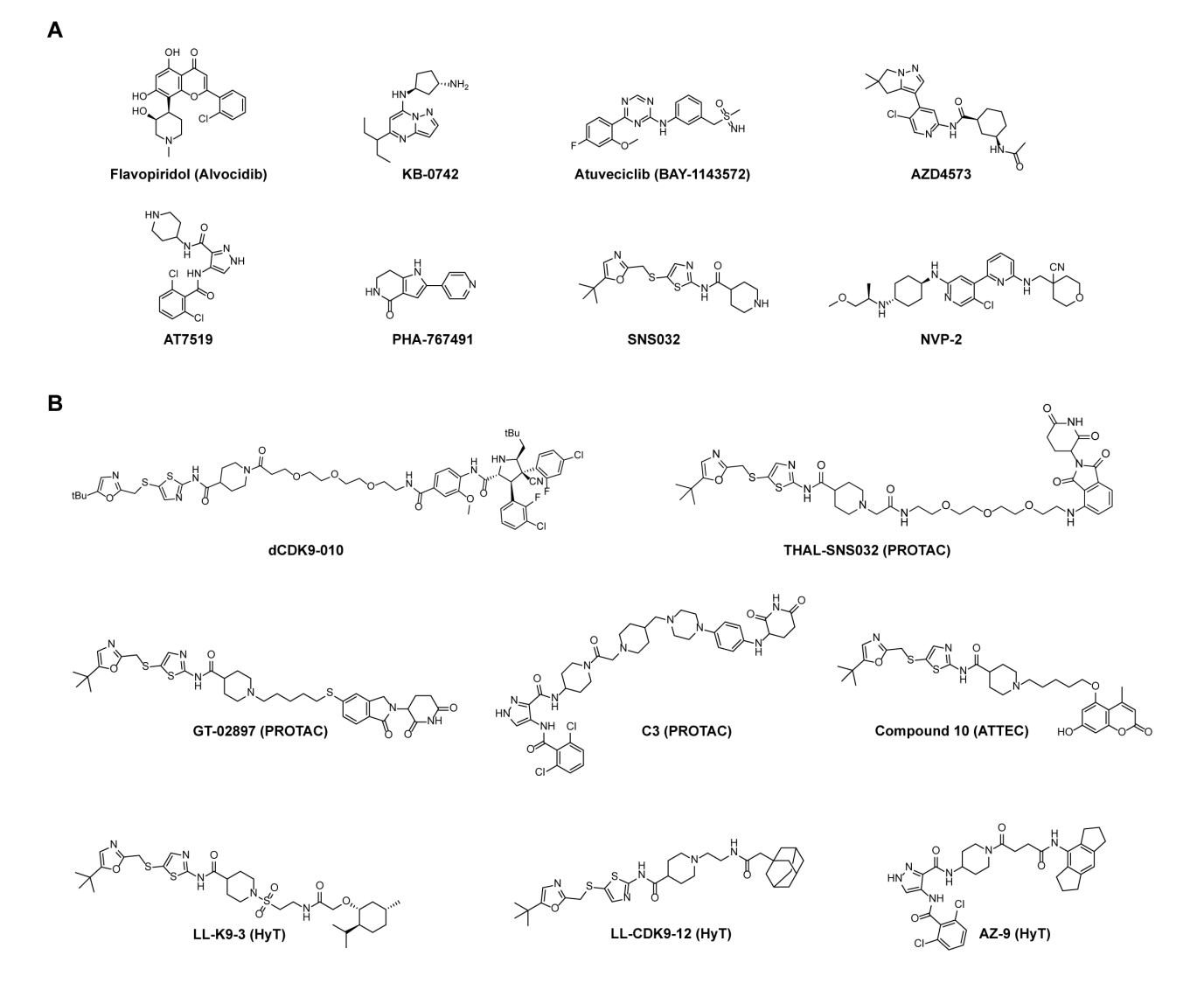
**

**Figure S7. Representative CDK9-targeting agents.**

**(A)** Representative CDK9 inhibitors.

**(B)** Structures of dCDK9-010 and representative CDK9 degraders. ATTEC, autophagy-tethering compound; HyT, hydrophobic tag.


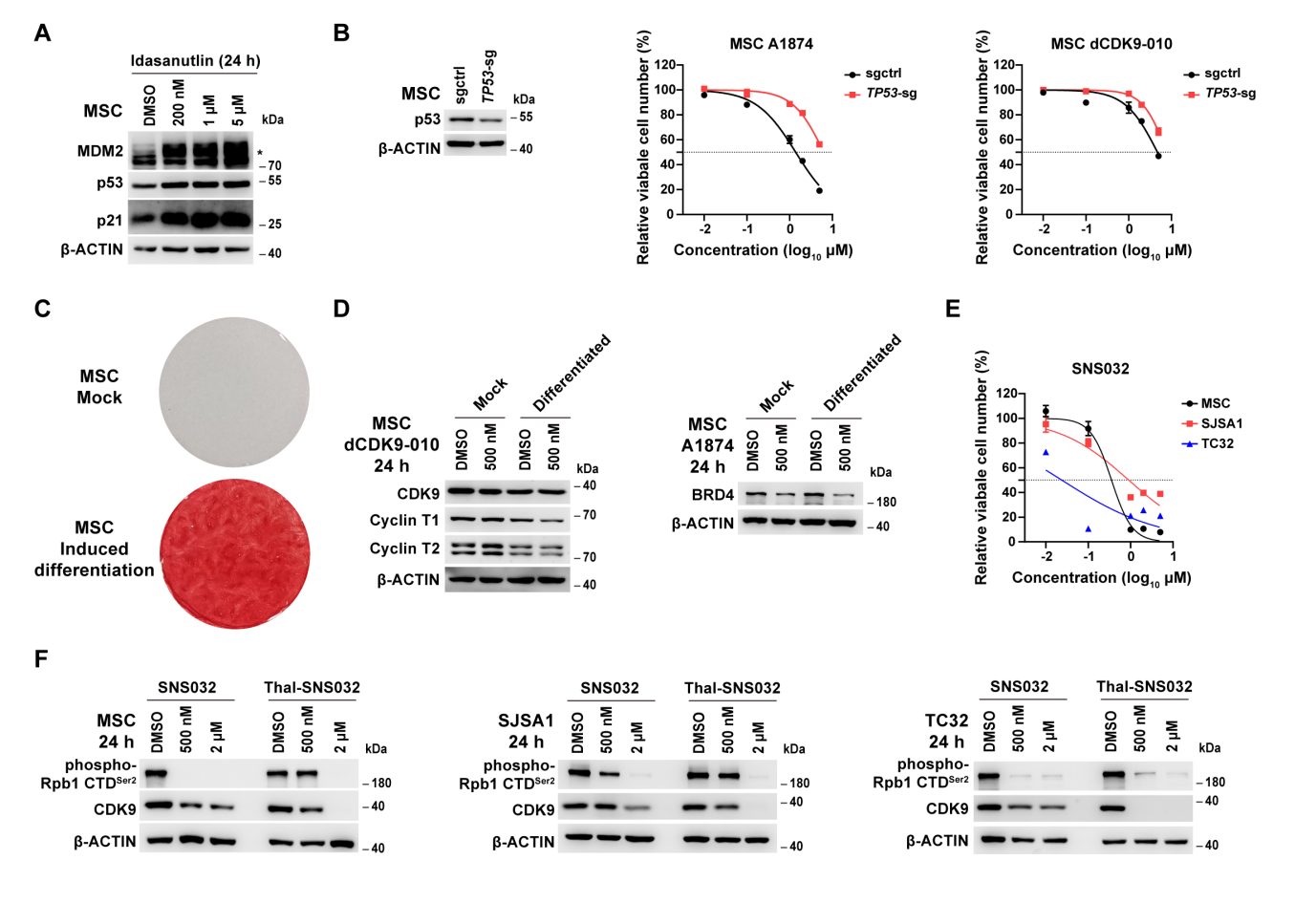


**Figure S8. MSCs exhibit reduced sensitivity to MDM2-TEMADs.**

**(A)** Dose-dependent induction of p53 and p21 by Idasanutlin in MSCs, indicative of a functional MDM2-p53 axis.

**(B)** Effect of CRISPR/Cas9-mediated *TP53* silencing on MSC viability in response to MDM2-TEMADs. Data are presented as mean±SEM; n=3.

**(C)** Alizarin Red S staining of MSCs cultured under osteogenic differentiation condition.

**(D)** Efficacy of MDM2-TEMADs in degrading their target proteins or inhibiting phospho-Rpb1 CTD^Ser2^ in MSCs versus osteogenically differentiated counterparts.

**(E)** Dose-response curve of cells treated with the CDK9 inhibitor SNS032. Data are presented as mean±SEM; n=3.

**(F)** Responsiveness of phospho-Rpb1 CTD^Ser2^ to SNS032 and CDK9 degrader Thal-SNS032 in MSCs and sarcoma cells.

**Supplementary Table S6. Structure information of dCDK9-001~dCDK9-010**

| **Compound** | **Structure** |
| --- | --- |
| **dCDK9-001** |  |
| **dCDK9-002** |  |
| **dCDK9-003** |  |
| **dCDK9-004** |  |
| **dCDK9-005** |  |
| **dCDK9-006** |  |
| **dCDK9-007** |  |
| **dCDK9-008** |  |
| **dCDK9-009** |  |
| **dCDK9-010** |  |

**Supplementary Table S9. List of cell lines**

| **Cell lines** | **RRID or Provider** |
| --- | --- |
| A673 | RRID:CVCL_0080 |
| CADO-ES1 | RRID:CVCL_1103 |
| HOS | RRID:CVCL_0312 |
| MG63 | RRID:CVCL_0426 |
| SAOS2 | RRID:CVCL_0548 |
| SJSA1 | RRID:CVCL_1697 |
| SKNMC | RRID:CVCL_0530 |
| TC32 | RRID:CVCL_7151 |
| TC71 | RRID:CVCL_2213 |
| U2OS | RRID:CVCL_0042 |
| ES2 | RRID:CVCL_AX39  (Kindly provided by Dr Peter Houghton) |
| ES6 | RRID:CVCL_1202  (Kindly provided by Dr Peter Houghton) |
| H161 | RRID:CVCL_C834  Kindly provided by Dr Emil Bogenmann |
| HCT116 | RRID:CVCL_0291 |
| HCT116*^TP53-/-^* | Kindly provided by Dr Phillip Koeffler |
| MSC | Kindly provided by Dr Phillip Koeffler |
| HEK293T | RRID:CVCL_0063 |
| ZCH002M | This study |

Footnote: the exact dates of cell line purchase or establishment are not available.

**Supplementary Table S10. List of cloning vectors**

| **Plasmids** | **RRID or Provider** |
| --- | --- |
| pCDH-GFP-BSD | This study |
| pLV3-BRD4(FL)-3×HA | This study |
| pLV3-EGFP-BRD4(Cs)-3×FLAG | This study |
| pLV3-ETS2-3×HA | This study |
| pCDH-HA-CDK9 | This study |
| pCDH-MDM2-3×FLAG | This study |
| lentiCRISPR v2 | RRID:Addgene_52961 |
| pLKO.1 puro | RRID:Addgene_8453 |

**Supplementary Table S11. Sequences of shRNAs and sgRNAs**

| **shRNA** | **Sequence (5’ → 3’)** |
| --- | --- |
| MDM2-sh1 | CCGGTAGTATAATTGACCTACTTTGCTCGAGCAAAGTAGGTCAATTATACTATTTTTG |
| MDM2-sh2 | CCGGCTCAGCCATCAACTTCTAGTACTCGAGTACTAGAAGTTGATGGCTGAGTTTTTG |
| BRD4-sh1 | CCGGGCCAAATGTCTACACAGTATACTCGAGTATACTGTGTAGACATTTGGCTTTTTG |
| BRD4-sh2 | CCGGTGAACCTCCCTGATTACTATACTCGAGTATAGTAATCAGGGAGGTTCATTTTTG |
| CDK9-sh1 | CCGGAGGGACATGAAGGCTGCTAATCTCGAGATTAGCAGCCTTCATGTCCCTTTTTTG |
| CDK9-sh2 | CCGGGTTCGACTTCTGCGAGCATGACTCGAGTCATGCTCGCAGAAGTCGAACTTTTTG |
| ETS2-sh1 | CCGGGCCGACTAAGAGAAGTTGTAACTCGAGTTACAACTTCTCTTAGTCGGCTTTTTG |
| ETS2-sh2 | CCGGCGCCAACTGTGAATTGCCTTTCTCGAGAAAGGCAATTCACAGTTGGCGTTTTTG |
| CEBPB-sh1 | CCGGCCCGTGGTGTTATTTAAAGAACTCGAGTTCTTTAAATAACACCACGGGTTTTTG |
| CEBPB-sh2 | CCGGGCACAGCGACGAGTACAAGATCTCGAGATCTTGTACTCGTCGCTGTGCTTTTTG |
| SNAI2-sh1 | CCGGGAGTGACGCAATCAATGTTTACTCGAGTAAACATTGATTGCGTCACTCTTTTTG |
| SNAI2-sh2 | CCGGCCGAAGCCAAATGACAAATAACTCGAGTTATTTGTCATTTGGCTTCGGTTTTTG |
| SOX9-sh1 | CCGGGCATCCTTCAATTTCTGTATACTCGAGTATACAGAAATTGAAGGATGCTTTTTG |
| SOX9-sh2 | CCGGACCTTCGATGTCAACGAGTTTCTCGAGAAACTCGTTGACATCGAAGGTTTTTTG |
| RUNX2-sh1 | CCGGGCTACCTATCACAGAGCAATTCTCGAGAATTGCTCTGTGATAGGTAGCTTTTTG |
| RUNX2-sh2 | CCGGAGGTTCAACGATCTGAGATTTCTCGAGAAATCTCAGATCGTTGAACCTTTTTTG |
|  |  |
| **sgRNA** | **Sequence (5’ → 3’)** |
| sgRNA(ko)-CCNT1 | CACCGTGTGTACTGCAGGCTAAATG |
| sgRNA(ko)-CCNT2 | CACCGAAATGTACCCAGTTAGTAAG |
| sgRNA(ko)-MDM2 | CACCGAACAGGTGTCACCTTGAAGG |
| sgRNA(ko)-p53 | CACCGATGTGTAACAGTTCCTGCAT |

**Supplementary Table S12. List of chemicals**

| **Chemical** | **Brand** | **Cat #** |
| --- | --- | --- |
| DMSO | Sigma-Aldrich | D4540 |
| Nutlin-3a | MedChemExpress | HY-10029 |
| Idasanutlin | TargetMol Chemicals Inc. | T6254 |
| JQ1 | Tocris | 4499 |
| SNS032 | TargetMol Chemicals Inc. | T6049 |
| (R)-MG132 | TargetMol Chemicals Inc. | T12628 |
| A1874 | DC Chemicals | DC12702 |
| ARV-771 | MedChemExpress | HY-100972 |
| ARV-825 | TargetMol Chemicals Inc. | T5434 |
| dBET6 | MedChemExpress | HY-112588 |
| MZ1 | Tocris | 6154 |
| ZBC260 | MedChemExpress | HY-101519 |
| ZXH-3-26 | MedChemExpress | HY-122826 |
| Niraparib | MedChemExpress | HY-10619 |
| Niraparib | TargetMol Chemicals Inc. | T3231 |
| Puromycin | Beyotime | ST551 |
| Blasticidin S | Beyotime | ST018 |
| Formaldehyde | Sigma-Aldrich | F8775 |
| Disuccinimidyl glutarate | Santa Cruz Biotechnology | sc-285455 |
| Protease inhibitor cocktail | TargetMol Chemicals Inc. | C0001 |
| Phosphatase inhibitor cocktail | TargetMol Chemicals Inc. | C0004 |
| Solutol HS-15 | Sigma-Aldrich | 42966 |

**Supplementary Table S13. List of antibodies**

| **Antibody** | **Brand** | **Cat #** | **RRID** |
| --- | --- | --- | --- |
| MDM2 | GeneTex | GTX100531 | RRID: AB_10624270 |
| p53 | Santa Cruz Biotechnology | sc-126 (DO-1) | RRID: AB_628082 |
| CDK9 | Santa Cruz Biotechnology | sc-13130 (D-7) | RRID: AB_627245 |
| BRD2 | Cell Signaling Technology | 5848 | RRID: AB_10835146 |
| BRD3 | Proteintech | 11859-1-AP | RRID: AB_2065902 |
| BRD4 | Cell Signaling Technology | 13440 | RRID: AB_2687578 |
| Cyclin T1 | Cell Signaling Technology | 81464 | RRID: AB_2799973 |
| Cyclin T2 | Santa Cruz Biotechnology | sc-81243 (2128C1a) | RRID: AB_1122005 |
| β-ACTIN | Servicebio | GB15003-100 | RRID: AB_3716407 |
| β-ACTIN | Santa Cruz Biotechnology | sc-47778 (C4) | RRID: AB_626632 |
| GAPDH | Cell Signaling Technology | 2118 | RRID:AB_561053 |
| JUN | Santa Cruz Biotechnology | sc-398615 (G-7) | RRID: AB_3717591 |
| Histone H3 | Cell Signaling Technology | 4499 | RRID: AB_10544537 |
| HA | Cell Signaling Technology | 3724 | RRID:AB_1549585 |
| α-Tubulin | Cell Signaling Technology | 2144 | RRID: AB_2210548 |
| Total-Rbp1 | Diagbio | db15872 | RRID: AB_3717592 |
| Total-Rbp1 | Cell Signaling Technology | 2629 | RRID:AB_2167468 |
| phospho-Rpb1 CTD^Ser2^ | Cell Signaling Technology | 13499 | RRID: AB_2798238 |
| RUNX2 | Santa Cruz Biotechnology | sc-390351 (F-2) | RRID: AB_2892645 |
| SNAI2 | Cell Signaling Technology | 9585 | RRID: AB_2239535 |
| CDK1/CDK2 | Santa Cruz Biotechnology | sc-53219 (AN21.2) | RRID: AB_2120095 |
| CDK4 | Cell Signaling Technology | 12790 | RRID: AB_2631166 |
| CDK5 | Santa Cruz Biotechnology | sc-173 (C-8) | RRID: AB_631224 |
| CDK6 | Santa Cruz Biotechnology | sc-53638 (DCS-83) | RRID: AB_1121424 |
| CDK7 | Santa Cruz Biotechnology | sc-7344 (C-4) | RRID: AB_627243 |
| CDK8 | Santa Cruz Biotechnology | sc-13155 (D-9) | RRID: AB_627244 |
| CDK11B | ABclonal | A12830 | RRID: AB_2759670 |
| PALB2 | Sangon Biotech | D261065 | RRID: AB_3717595 |
| p21 Waf1/Cip1 | Cell Signaling Technology | 2947 | RRID:AB_823586 |
| BRCA2 | HUABIO | HA722850 | RRID: AB_3717597 |
| RAD51 | GeneTex | GTX100469 | RRID: AB_1951602 |
| Cleaved-Caspase 3 | Diagbio | db15982 | RRID: AB_3717593 |
| Cleaved-PARP | Cell Signaling Technology | 5625 | RRID: AB_10699459 |
| phospho-Histone H3 ^Ser10^ | Cell Signaling Technology | 9701 | RRID: AB_331535 |
| CHK1 | HUABIO | HA722855 | RRID: AB_3717598 |
| ETS2 | Santa Cruz Biotechnology | sc-365666 (E-5) | RRID: AB_10841726 |
| CEBPB | Santa Cruz Biotechnology | sc-7962 (H-7) | RRID: AB_626772 |
| γ-H2AX | Diagbio | db13191 | RRID: AB_3717594 |
| H3K27ac | Active motif | 39133 | RRID: AB_2561016 |
| Ki67 | DAKO | M7240 | RRID: AB_2142367 |
| SOX9 | HUABIO | ET1611-56 | RRID: AB_2924312 |
| Peroxidase AffiniPure® Goat Anti-Mouse IgG (H+L) | Jackson Immuno Research | 115-035-003 | RRID: AB_10015289 |
| Peroxidase AffiniPure® Goat Anti-Rabbit IgG (H+L) | Jackson Immuno Research | 111-035-003 | RRID: AB_2313567 |

**Supplementary Table S14. Sequences of qPCR primers**

| **qPCR primer** | **Sequence (5’ → 3’)** |
| --- | --- |
| PALB2-F | ATCCTGCTAGATCACCAGTAACT |
| PALB2-R | TCAAGGTGCTGACTACTACCG |
| RFWD3-F | CCACCGGCTCTCAGCATTAC |
| RFWD3-R | ACTGGGGACATTTTCGTACTTG |
| BRCA1-F | TTGTTACAAATCACCCCTCAAGG |
| BRCA1-R | CCCTGATACTTTTCTGGATGCC |
| BRCA2-F | ACAAGCAACCCAAGTGTCAAT |
| BRCA2-R | TGAAGCTACCTCCAAAACTGTG |
| β-ACTIN-F | GACGACATGGAGAAAATCTG |
| β-ACTIN-R | ATGATCTGGGTCATCTTCTC |
| AURKA-F | CCTACAAAAGAATATCACGGG |
| AURKA-R | CAAGTACTTCTCTGAGCATTG |
| RUNX2-F | GATGGGACTGTGGTTACTGTCAT |
| RUNX2-R | TCTCATACTGGGATGAGGAATGC |
| SNAI2-F | TGCATATTCGGACCCACACATTA |
| SNAI2-R | ATTTGACCTGTCTGCAAATGCTC |
| ETS2-F | CCCCTGTGGCTAACAGTTACA |
| ETS2-R | AGGTAGCTTTTAAGGCTTGACTC |
| CEBPB-F | ATAAACTCTCTGCTTCTCCC |
| CEBPB-R | CCGTAGGAACATCTTTAAGC |
| SOX9-F | AAGTCGGTGAAGAACGGGCAG |
| SOX9-R | GAAGGTCTCGATGTTGGAGATGACG |
